# Structure-Based and Molecular Modeling Studies for the Discovery of Cyclic Imides as Reversible Cruzain Inhibitors With Potent Anti-*Trypanosoma cruzi* Activity

**DOI:** 10.3389/fchem.2019.00798

**Published:** 2019-11-25

**Authors:** Rafael A. A. Ferreira, Ivani Pauli, Thiago S. Sampaio, Mariana L. de Souza, Leonardo L. G. Ferreira, Luma G. Magalhães, Celso de O. Rezende, Rafaela S. Ferreira, Renata Krogh, Luiz C. Dias, Adriano D. Andricopulo

**Affiliations:** ^1^Instituto de Química, Universidade Estadual de Campinas, Campinas, Brazil; ^2^Laboratório de Química Medicinal e Computacional, Centro de Pesquisa e Inovação em Biodiversidade e Fármacos, Instituto de Física de São Carlos, Universidade de São Paulo, São Carlos, Brazil; ^3^Departamento de Bioquímica e Imunologia, Universidade Federal de Minas Gerais, Belo Horizonte, Brazil

**Keywords:** Chagas disease, *Trypanosoma cruzi*, cruzain, SAR, medicinal chemistry, synthesis, inhibitors, molecular docking

## Abstract

Chagas disease causes ~10,000 deaths each year, mainly in Latin America, where it is endemic. The currently available chemotherapeutic agents are ineffective in the chronic stage of the disease, and the lack of pharmaceutical innovation for Chagas disease highlights the urgent need for the development of new drugs. The enzyme cruzain, the main cysteine protease of *Trypanosoma cruzi*, has been explored as a validated molecular target for drug discovery. Herein, the design, molecular modeling studies, synthesis, and biological evaluation of cyclic imides as cruzain inhibitors are described. Starting with a micromolar-range cruzain inhibitor (**3a**, IC_50_ = 2.2 μM), this molecular optimization strategy resulted in the nanomolar-range inhibitor **10j** (IC_50_ = 0.6 μM), which is highly active against *T. cruzi* intracellular amastigotes (IC_50_ = 1.0 μM). Moreover, most compounds were selective toward *T. cruzi* over human fibroblasts, which were used as host cells, and are less toxic to hepatic cells than the marketed drug benznidazole. This study enabled the discovery of novel chemical diversity and established robust structure-activity relationships to guide the design of optimized cruzain inhibitors as new trypanocidal agents.

## Introduction

Caused by the protozoan *Trypanosoma cruzi* and endemic in 21 countries in Latin America, Chagas disease kills ~10,000 people each year^1^. This neglected tropical disease has reached non-endemic regions, affecting 8 million people worldwide and putting another 25 million at risk of infection^1^. The USA shows the greatest burden among non-endemic countries, with ~300,000 people estimated to be infected with *T. cruzi* (Pérez-Molina and Molina, 2018). In addition, Chagas disease is a major cause of infectious cardiomyopathy worldwide, contributing substantially to the global burden of cardiovascular disease (Bern, 2015; Cucunubá et al., 2016). Mortality and a reduction in productivity of the affected populations significantly impact the economies of the endemic regions. These economic and social burdens can be translated in numbers that estimate losses of more than US $7.2 billion per year and ~243,600 disability-adjusted life years (DALYs) due to Chagas disease (GBD DALYs and HALE Collaborators, 2016; Arnal et al., 2019).

Even more than a century after the discovery of Chagas disease by Brazilian physician Carlos Chagas in 1909, current chemotherapy for this condition relies on two drugs only—benznidazole (**BZ**) and nifurtimox (Dias et al., 2014). These nitroheterocyclic compounds, which were identified between the mid-1960s and 1970s, are effective only when administered during the acute stage of the disease, a limitation that leaves millions of chronic chagasic patients without appropriate treatment (Molina et al., 2015). Moreover, benznidazole and nifurtimox cause severe adverse effects in up to 40% of patients, leading to poor adherence to treatment (Pérez-Molina and Molina, 2018). These drawbacks highlight the urgent need for the development of effective and safe drugs for the therapy of Chagas disease (Olivera et al., 2015; Ferreira and Andricopulo, 2019).

The enzyme cruzain (EC 3.4.22.51) is the main cysteine protease of *T. cruzi* and has been explored as a validated molecular target in Chagas disease drug discovery (McKerrow, 1999; Jose Cazzulo et al., 2001). It is expressed throughout the life cycle of *T. cruzi* and is involved in critical biological processes such as the interaction with host cells, parasite reproduction and evasion from the host immunologic system (Engel et al., 1998; Ferreira and Andricopulo, 2017). Cruzain has been validated as a molecular target for Chagas disease drug discovery based on genetic studies of *T. cruzi* and the ability of cruzain inhibitors to decrease parasite burden *in vivo* (Doyle et al., 2011; Ndao et al., 2014). These studies have recently supported the design and identification of several classes of cruzain inhibitors, including vinyl sulfones, triazoles, pyrimidines, thiosemicarbazones, chalcones, nitroalkenes, and benzimidazoles (Rogers et al., 2012; Ferreira et al., 2014; Avelar et al., 2015; Espíndola et al., 2015; Neitz et al., 2015; Latorre et al., 2016). The vinyl sulfone K777, which is a covalent cruzain inhibitor, showed promising results in preclinical efficacy tests; however, toxicity-related drawbacks prevented the compound from progressing into advanced clinical development (Ndao et al., 2014). The poor safety profile of K777 was associated with the irreversible mode of action of the compound. Following the failure of K777, the pursuit of novel cruzain inhibitors has recently focused on the design of reversible ligands. These investigations, along with the available structural data of cruzain bound with small molecule ligands, have been key to promoting the discovery of novel classes of inhibitors with improved safety profiles. Moreover, these data have enabled the integration of experimental and computational approaches into robust structure-based drug design (SBDD) campaigns that have been key to identifying novel chemical diversity to be explored in Chagas disease drug discovery.

## Materials and Methods

### Molecular Docking

The three-dimensional structures of the designed cruzain inhibitors were constructed using the standard geometric parameters of SYBYL-X 2.1 (Certara, Princeton, NJ). Each compound was energetically minimized using the Tripos force field (Clark et al., 1989) and Powell conjugate gradient algorithm (Powell, 1977) with a convergence criterion of 0.05 kcal/mol.Å and Gasteiger-Hückel charges (Gasteiger and Marsili, 1980). The designed imide derivatives were docked into the cruzain catalytic site using GOLD 5.3 (Cambridge Crystallographic Data Centre, Cambridge, UK) (Jones et al., 1997). The X-ray structure of cruzain (PDB 3KKU, 1.28 Å) (Ferreira et al., 2010) was prepared by removing the water molecules and adding hydrogen atoms. The active site residues Cys25 and His162 were maintained as negatively charged and protonated, respectively. A sphere with a 10 Å radius centered on the sulfur atom of Cys25 was settled as the binding site. Compounds were docked by applying the GoldScore scoring function with a search efficiency of 200%. Visual analysis of the molecular docking-derived binding conformations was carried out with PyMOL 1.3 (Schrödinger, New York, NY) (Lill and Danielson, 2011).

### Pro-Cruzain Expression, Activation, and Purification

Cruzain was expressed and purified using a modified version of a previously published protocol (Ferreira et al., 2014). *Escherichia coli* (M15) was preinoculated in Luria Bertani (LB) medium with ampicillin (100 μg/mL) and kanamycin (50 μg/mL) and kept overnight (37°C, 200 rpm). The preinoculum was diluted 10-fold in fresh LB medium (1 L) supplemented with 0.5 M NaCl, 0.2% glucose, 1 mM betaine, 0.5 M sorbitol, 100 μg/mL ampicillin and 50 μg/mL kanamycin, and incubated (37°C, 200 rpm). When the optical density (OD_600_) reached 0.9, the culture was incubated at 47°C for 20 min to induce the expression of chaperones. Next, cruzain expression was induced by adding 0.2 mM isopropyl β-D-thiogalactopyranoside (IPTG), and the culture was kept overnight at 20°C. The cells were harvested by centrifugation (5,000 rpm, 30 min, 4°C) and suspended in 50 mL of lysis buffer (300 mM NaCl, 50 mM Tris-HCl, 1.6 mg/mL lysozyme, pH 8.0). The cells were then lysed by sonication (12 cycles of 30 s), and the suspension was centrifuged (9,000 rpm, 30 min, 4°C). The supernatant was collected, cruzain was precipitated by the addition of 35% ammonium sulfate (2 h), and this suspension was centrifuged (9,000 rpm, 30 min, 4°C). The precipitate was resuspended in lysis buffer and dialyzed to remove the ammonium sulfate. The soluble fraction of cruzain was purified by metal affinity chromatography using a Ni–NTA resin (Qiagen, Hilden, Germany). Contaminant proteins were removed using a washing buffer (300 mM NaCl, 50 mM Tris-HCl, 10 mM imidazole, pH 8.0). Next, cruzain was eluted with an increasing gradient of imidazole (25, 50, 75, 100, and 250 mM). The fractions containing cruzain were dialyzed in 0.1 M acetate buffer (1.5 L, pH 5.5) and concentrated to 0.5 mg/mL for subsequent activation.

Pro-cruzain was incubated in activation buffer (100 mM sodium acetate, 10 mM EDTA, 5 mM DTT, and 1 M NaCl, pH 5.5) at 37°C (Mott et al., 2010). The activation process was monitored at 30 min intervals by the cleavage of the substrate Z-Phe-Arg-AMC and confirmed by SDS-PAGE. Next, the mature enzyme was diluted 20-fold in binding buffer (20 mM sodium phosphate, 150 mM NaCl, pH 7.2) and incubated overnight with thiopropyl Sepharose 6B resin (GE Healthcare Life Sciences, Pittsburgh, PA) at 4°C. Cruzain was eluted using binding buffer supplemented with 20 mM DTT. Fractions containing the enzyme were stored at −80°C in 0.1 M sodium acetate (pH 5.5).

### Cruzain Inhibition Assays

The catalytic activity of cruzain was monitored by cleavage of the fluorogenic substrate Z-Phe-Arg-aminomethyl coumarin (Z-Phe-Arg-AMC) as previously described (Ferreira et al., 2014). The enzyme kinetics assays were performed in 0.1 M sodium acetate buffer (pH 5.5) with 5 mM dithiothreitol (DTT) and 0.01% Triton X-100. The final concentrations of cruzain and substrate (*K*_m_ = 1.6 μM) were 1.5 nM and 5.0 μM, respectively, except in the tests for the mechanism of inhibition, in which different substrate concentrations were used. The enzyme reaction was monitored for 5 min at 30°C in 96-well black flat bottom plates, and the activity was calculated based on the initial rates compared with a control (DMSO). Wavelengths of 355 nm for excitation and 460 nm for emission were used. IC_50_ values were independently determined by determining rate measurements for at least six inhibitor concentrations, each evaluated in triplicate. To determine the mechanism of inhibition, eight substrate concentrations and four inhibitor concentrations were used, each in triplicate. The mechanism of inhibition was determined by Lineweaver-Burk plots. SigmaPlot 10.0 (Systat Software Inc., Erkrath, Germany) was used to determine the IC_50_ values.

### *T. cruzi* Intracellular Amastigote Assays

*In vitro* assays against *T. cruzi* were performed as described previously (Ferreira et al., 2014). The *T. cruzi* Tulahuen strain, which expresses the *E. coli* β-galactosidase gene *lacZ* (Buckner et al., 1996), was provided by Frederick S. Buckner (University of Washington, Seattle, WA). Stock solutions of the synthesized compounds were prepared in 100% DMSO. Epimastigotes were grown in liver infusion tryptone (LIT) supplemented with 10% fetal calf serum (FCS), penicillin and streptomycin (28°C). Metacyclogenesis from epimastigotes to trypomastigotes was induced by incubation of the epimastigotes in Grace's insect medium (Sigma-Aldrich, St. Louis, MO) supplemented with 10% FCS (28°C). HFF-1 human fibroblasts were seeded at 2 × 10^3^/well (80 μL) in 96-well tissue culture plates in RPMI 1640 without phenol red supplemented with 10% FCS and incubated overnight (37°C, 5% CO_2_). Next, trypomastigotes were added at 1.0 × 10^4^/well (20 μL), and the plates were incubated (37°C, 5% CO_2_). After 24 h, 3-fold serial dilutions (50 μL) of the synthesized compounds were added at concentrations ranging from 0.1 to 100 μM, and the plates were incubated (37°C, 5% CO_2_). Each compound concentration was evaluated in triplicate. All plates included **BZ** (Sigma-Aldrich) as a positive control and untreated wells (100% growth) as a negative control. After 120 h, 50 μL of chlorophenol red β-D-galactopyranoside (CPRG, Sigma-Aldrich) and IGEPAL CA-630 (Sigma-Aldrich) (0.1%) was added to each well. The absorbance was measured at 570 nm in an automated microplate reader, and the data were transferred to SigmaPlot 10.0 (Systat Software Inc.) for IC_50_ value calculation.

### Cytotoxicity in HFF-1 and HepG2 Cell Lines

The cytotoxicities of the compounds against HFF-1 fibroblasts and HepG2 hepatocytes were evaluated using the MTS tetrazolium assay (Promega, Madison, WI) (Barltrop et al., 1991). HFF-1 fibroblasts were seeded at 2 × 10^3^/well in 96-well culture plates in RPMI 1640 without phenol red supplemented with 10% FCS and incubated overnight (37°C, 5% CO_2_). HepG2 hepatocytes were seeded at 6 × 10^3^/well in 96-well culture plates in DMEM (Cultilab, Campinas SP) supplemented with 10% FCS and incubated overnight (37°C, 5% CO_2_). The compounds were added in 3-fold serial dilutions, and the plates were incubated at 37°C with 5% CO_2_. Each compound concentration was evaluated in triplicate. All plates included doxorubicin (Sigma-Aldrich, St. Louis, MO) as a positive control and untreated wells (100% growth) as a negative control. After 72 h, 20 μL of MTS was added, and the plates were incubated for 4 h. The absorbance was measured at 490 nm in an automated microplate reader, and the data were transferred to SigmaPlot 10.0 (Systat Software Inc., Erkrath, Germany) for IC_50_ value calculation. The percent of nonviable cells was determined and compared to the negative control wells (100% growth).

### Chemistry

All reactions were performed under an argon atmosphere with dry solvents and magnetic stirring unless stated otherwise. Dichloromethane (DCM) and triethylamine (Et_3_N) were distilled from CaH_2_. Tetrahydrofuran (THF) was distilled from sodium/benzophenone. Dimethyl formamide (DMF) was purchased from Sigma-Aldrich (anhydrous) and used without further purification. Yields refer to homogeneous materials obtained after purification of the reaction products by flash column chromatography using silica gel (200–400 mesh) or recrystallization. Analytical thin-layer chromatography was performed on silica-gel 60 and GF (5–40 μm thickness) plates, and visualization was accomplished using UV light, basic potassium permanganate staining or ninhydrin solution followed by heating. ^1^H and proton-decoupled ^13^C NMR spectra were acquired in CDCl_3_, CD_3_OD, or *d*_6_-DMSO at 250 MHz (^1^H) and 62.5 MHz (^13^C) (Bruker DPX250), at 400 MHz (^1^H) and 100 MHz (^13^C) (Bruker Avance 400), at 500 MHz (^1^H) and 125 MHz (^13^C) (Varian Inova 500), or at 600 MHz (^1^H) and 150 MHz (^13^C) (Bruker Avance 600). Chemical shifts (δ) are reported in ppm using residual undeuterated solvent as an internal standard (CDCl_3_ at 7.26 ppm, CD_3_OD at 3.31 ppm, *d*_6_-DMSO at 2.50 ppm, and TMS at 0.00 ppm for ^1^H NMR spectra and CDCl_3_ at 77.16 ppm, CD_3_OD at 49.0 ppm, *d*_6_-DMSO at 39.52 ppm for ^13^C NMR spectra). Multiplicity data are reported as follows: s = singlet, d = doublet, t = triplet, q = quartet, br s = broad singlet, dd = doublet of doublets, dt = doublet of triplets, ddd = doublet of doublet of doublets and m = multiplet. The multiplicity is followed by the coupling constant(s) in Hz and integration. High-resolution mass spectrometry (HRMS) was measured using electrospray ionization (ESI) (Waters Xevo Q-TOF, Thermo LTQ-FT Ultra, or Thermo Q-Exactive) or electron ionization (EI) (GCT Premier, Waters).

### General Procedures for the Preparation of Alcohols 2a-m

Method A: The aniline derivative containing the appropriate substituents was dissolved in dry dichloromethane (DCM) under an argon atmosphere with magnetic stirring. The solution was cooled to 0°C followed by the slow addition of acetoxyacetyl chloride (1.1 equivalents). The mixture was stirred for 5 min before the addition of zinc mesh 20 (0.1 equivalents) and removal of the ice bath, allowing the reaction to reach room temperature. The reaction mixture was monitored by thin layer chromatography (TLC), and after 30 min, total conversion was observed. Half of the initial volume of DCM was added to dilute the reaction mixture, and the zinc was removed by filtration. The supernatant was washed with an aqueous sodium bicarbonate solution. The organic phase was dried over magnesium sulfate (MgSO_4_) and concentrated to dryness under vacuum. The product was purified by column chromatography on silica gel using a mixture of ethyl acetate/hexanes (50%) as the eluent. The obtained ester was dissolved in methanol at room temperature followed by the addition of solid potassium carbonate (K_2_CO_3_). The reaction mixture was stirred for 1 h and then quenched by the addition of ethyl acetate. The mixture was washed with aqueous saturated ammonium chloride (NH_4_Cl) solution and brine. The resulting organic phase was dried over MgSO_4_ and concentrated under reduced pressure. Purification was performed by column chromatography on silica gel using a mixture of ethyl acetate/hexanes (50%) as the eluent.

Method B: The aniline derivative containing the appropriate substituents was dissolved in dry DCM under an argon atmosphere with magnetic stirring. To the resulting solution, dry triethylamine (1.1 equivalents) and acetoxyacetyl chloride (1.1 equivalents) were added and stirring was maintained for 2 h to monitor the conversion by TLC. The mixture was then washed with saturated aqueous NH_4_Cl solution and brine solution. The organic phase was dried over MgSO_4_ and concentrated to dryness under vacuum. The obtained ester was dissolved in methanol at room temperature followed by the addition of solid potassium carbonate (K_2_CO_3_). The reaction mixture was stirred for 1 h and then quenched by the addition of ethyl acetate. The mixture was washed with aqueous saturated ammonium chloride (NH_4_Cl) solution and brine. The resulting organic phase was dried over MgSO_4_ and concentrated under reduced pressure. Purification was performed by column chromatography on silica gel using a mixture of ethyl acetate/hexanes (50%) as the eluent.

### General Procedures for Production of Carboxylic Acids 1 and 9a-n

Method C: The appropriate cyclic anhydride and amino acid (1.1 equivalents) were dissolved in glacial acetic acid under an argon atmosphere. The mixture was magnetically stirred overnight followed by 4 h of reflux periodically monitored by TLC. After consumption of the cyclic anhydride, the reaction pot was cooled to room temperature. Acetic acid was removed in a rotary evaporator, and ice-cold water was added to the resulting slurry to generate a precipitate. Concentrated hydrochloric acid (HCl) was added, and the solid was collected by filtration, followed by high vacuum drying.

Method D: The appropriate cyclic anhydride and amino acid (1.1 equivalents) were added to a reaction flask containing toluene. To this suspension, TEA (0.1 equivalents) was added and a Dean-Stark apparatus was coupled to the system. The mixture refluxed for 4 h and cooled to room temperature. Toluene was removed in a rotary evaporator, and the residue was dissolved in ethyl acetate and washed twice with 1 M HCl. The organic phase was extracted with saturated aqueous sodium bicarbonate solution. The aqueous phase was acidified with HCl solution to pH 5.0 and extracted with ethyl acetate. The combined organic phases were dried over MgSO_4_, concentrated under vacuum and used in the following step without further purification.

Method E: Leucine was dissolved in a 1:1 mixture of hydrobromic acid and water in an ice bath. Sodium nitrite (6.5 M aqueous solution) was added dropwise. The ice bath was removed, and the solution stirred at room temperature for 2.5 h. The acidic residues were removed under reduced pressure, and the remaining solution was extracted with diethyl ether 3 times. The combined organic phases were dried over MgSO_4_ and concentrated under vacuum.

The residue was dissolved in DCM under an argon atmosphere. The solution was cooled in an ice bath and benzyl alcohol (1.1 equivalents), 1-ethyl-3-(3-dimethylaminopropyl) carbodiimide (EDC) (1.1 equivalents) and 4-dimethylaminopyridine (DMAP) (0.1 equivalents) were added. The ice bath was removed, and the mixture stirred for 5 h. Then, the reaction mixture was washed with saturated aqueous NH_4_Cl solution and brine. The organic phase was dried over MgSO_4_ and concentrated under vacuum. The product was purified by column chromatography on silica gel using a mixture of ethyl acetate/hexanes (50%) as the eluent.

A solution of the pure ester was prepared in acetonitrile under an argon atmosphere, and the appropriate cyclic amine was added (2.0 equivalents). To the resulting solution, cesium carbonate (1.1 equivalents) was added followed by stirring for 2 h. The solids were removed by filtration, and the solvent was removed under reduced pressure followed by redissolution in DCM and washing with water. The organic phase was dried over MgSO_4_ and concentrated to dryness under vacuum. The product was purified by column chromatography on silica gel using a gradient from 0 to 4% methanol in DCM as the eluent.

The ester was dissolved in methanol, and 10% Pd/C was added (0.1 equivalents). The mixture stirred at room temperature under a hydrogen atmosphere (1 atm) for 30 min. The catalyst was removed by filtration over Celite®, and the solvent removed under reduced pressure to generate the pure carboxylic acid.

### General Procedure for the Preparation of Compounds 3a-m and 10a-n

Method F: A solution of carboxylic acid in dry DCM was prepared, and the alcohol was added (1.1 equivalents). The solution was cooled in an ice bath followed by the addition of EDC (1.2 equivalents) and DMAP (0.1 equivalents). The solution was allowed to warm to room temperature and stirred for 4 h while monitoring periodically by TLC. The reaction mixture was washed with saturated aqueous NH_4_Cl solution and brine. The organic phase was dried over MgSO_4_ and concentrated under reduced pressure. The product was purified by column chromatography on silica gel using a mixture of ethyl acetate/hexanes (50%) as the eluent.

#### 2-((3-chloro-4-methoxyphenyl)amino)-2-oxoethyl (2S)-2-(1,3-dioxo-1,3,3a,4,7,7a-hexahydro-2H-isoindol-2-yl)-4-methylpentanoate (3a)

Methods A and C followed by F

Very viscous liquid, 90% ^1^H NMR (500 MHz, CDCl_3_) δ 8.26 (sl, 1H); 7.83 (d, *J* = 2.5 Hz, 1H); 7.68 (dd, *J* = 9.0, 2.5 Hz, 1H); 6.90 (d, *J* = 9.0 Hz, 1H); 5.90 (m, 2H); 5.04 (d, *J* = 15.6 Hz, 1H); 4.88 (dd, *J* = 11.0, 4.4 Hz, 1H); 4.40 (d, *J* = 15.6 Hz, 1H); 3.89 (s, 3H); 3.23 (m, 2H); 2.65 (m, 2H); 2.27 (m, 2H); 2.08 (m, 1H); 1.94 (m, 1H); 1.41 (m, 1H); 0.93 (d, *J* = 6.6 Hz, 6H), ^13^C NMR (125 MHz, CDCl_3_) δ 180.4; 180.3; 168.1; 164.7; 151.9; 131.1; 127.8; 127.7; 122.3; 122.2; 119.4; 112.1; 63.4; 56.3; 51.0; 39.3; 39.2; 37.2; 24.7; 23.5; 23.4; 23.0; 20.9.

HRMS (ESI-Orbitrap): [M+H]^+^ Calculated for C_23_H_27_O_6_N_2_Cl 463.1617; found 463.1636.

#### 2-((4-methoxyphenyl)amino)-2-oxoethyl (2S)-2-(1,3-dioxo-1,3,3a,4,7,7a-hexahydro-2H-isoindol-2-yl)-4-methylpentanoate (3b)

Methods B and C followed by F

Very viscous liquid, 88% ^1^H NMR (250 MHz, DMSO) δ 9.78 (sl, 1H); 7.46 (d, *J* = 9.0 Hz, 1H); 6.89 (d, *J* = 9.0 Hz, 1H); 5.85 (m, 1H); 4.79 (dd, *J* = 11.1, 4.4 Hz, 1H); 4.63 (m, 2H); 3.72 (s, 3H); 3.24 (m, 2H); 2.39 (m, 2H); 2.20 (m, 2H); 1.99 (m, 1H); 1.77 (m, 1H); 1.34 (m, 1H); 0.84 (m, 6H), ^13^C NMR (62.5 MHz, DMSO) δ 179.6; 179.5; 168.5; 164.3; 155.5; 131.3; 127.4; 120.4; 113.9; 63.4; 55.2; 50.2; 36.0; 24.1; 23.2; 23.0; 20.7.

HRMS (ESI-Orbitrap): [M+H]^+^ Calculated for C_23_H_28_O_6_N_2_ 429.2026; found 429.2039.

#### 2-((3-chlorophenyl)amino)-2-oxoethyl (2S)-2-(1,3-dioxo-1,3,3a,4,7,7a-hexahydro-2H-isoindol-2-yl)-4-methylpentanoate (3c)

Methods B and C followed by F

Very viscous liquid, 91% ^1^H NMR (250 MHz, DMSO) δ 10.15 (m, 1H); 7.77 (m, 1H); 7.44 (m, 1H); 7.33 (m, 1H); 7.11 (m, 1H); 5.84 (m, 2H); 5.03 (m, 1H); 4.67 (m, 2H); 3,59 (m, 2H); 3.23 (m, 1H); 2.43 (m, 3H); 2.19 (m, 2H); 1.97 (m, 1H); 1.75 (m, 1H); 1.34 (m, 1H); 0.83 (m, 6H). ^13^C NMR (62.5 MHz, DMSO) δ 179.6; 179.5; 179.4; 173.3; 173.2; 172.4; 169.2; 168.6; 165.7; 165.2; 139.8; 139.7; 133.2; 130.5; 127.5; 127.4; 125.0; 124.9; 123.4; 123.3; 118.8; 117.7; 63.4; 62.7; 52.3; 51.6; 51.5; 50.3; 50.2; 38.4; 36.1; 36.0; 25.3; 24.2; 24.1; 23.2; 23.1; 23.0; 20.7.

HRMS (ESI-Orbitrap): [M+Na]^+^ Calculated for C_22_H_25_O_5_N_2_ClNa 455.1350; found 455.1362.

#### 2-oxo-2-(phenylamino)ethyl (2S)-2-(1,3-dioxo-1,3,3a,4,7,7a-hexahydro-2H-isoindol-2-yl)-4-methylpentanoate (3d)

Methods B and C followed by F

Very viscous liquid, 78% ^1^H NMR (400 MHz, DMSO) δ 9.97 (s, 1H); 7.55 (m, 2H); 7.32 (m, 2H); 7.07 (m, 1H); 5.85 (m,2H); 4.79 (dd, *J* = 11.2, 4.4 Hz, 1H); 4.67 (m, 2H); 3.25 (m, 2H); 2.39 (m, 2H); 2.20 (dd, *J* = 14.9, 7.2 Hz, 2H); 2.03 (m, 1H); 1.77 (ddd, *J* = 14.2, 10.1, 4.5 Hz, 1H); 1.35 (m, 1H); 0.84 (dd, *J* = 7.6, 6.6 Hz, 6H). ^13^C NMR (100 MHz, DMSO) δ 179.6; 168.5; 164.8; 138.2; 128.8; 127.4; 123.7; 119.3; 63.4; 50.2; 38.4; 36.0; 24.1; 23.2; 23.0; 20.7.

#### 2-((4-fluorophenyl)amino)-2-oxoethyl (2S)-2-(1,3-dioxo-1,3,3a,4,7,7a-hexahydro-2H-isoindol-2-yl)-4-methylpentanoate (3e)

Methods B and C followed by F

Very viscous liquid, 82% ^1^H NMR (250 MHz, DMSO) δ 10.02 (s, 1H); 7.56 (dd, *J* = 8.6, 5.0 Hz, 2H); 7.15 (t, *J* = 8.6 Hz, 2H); 5.84 (m, 2H); 4.79 (dd, *J* = 10.9, 4.3 Hz, 1H); 4.65 (m, 2H); 3.24 (m, 2H); 2.43 (m, 2H); 2.20 (m, 2H); 2.02 (m, 1H); 1.76 (m, 1H); 1.35 (m, 1H); 0.84 (m, 6H).

HRMS (ESI-Orbitrap): [M+H]^+^ Calculated for C_22_H_25_O_5_N_2_F 417.1826; found 417.1817.

#### 2-((4-bromophenyl)amino)-2-oxoethyl (2S)-2-(1,3-dioxo-1,3,3a,4,7,7a-hexahydro-2H-isoindol-2-yl)-4-methylpentanoate (3f)

Methods B and C followed by F

Very viscous liquid, 84% ^1^H NMR (250 MHz, DMSO) δ 10.13 (s, 1H); 7.53 (m, 4H); 5.84 (m, 2H); 4.78 (dd, *J* = 11.1, 4.5 Hz, 1H); 4.66 (m, 2H); 3.27 (m, 2H); 2.39 (m, 2H); 2.19 (m, 2H); 2.02 (m, 1H); 1.76 (m, 1H); 1.33 (m, 1H); 0.83 (m, 6H). ^13^C NMR (62.5 MHz, DMSO) δ 179.7; 179.6; 168.6; 165.0; 137.7; 131.7; 127.6; 127.5; 121.3; 115.4; 63.4; 50.3; 36.0; 24.1; 23.2; 23.1; 20.8.

HRMS (ESI-Orbitrap): [M+K]^+^ Calculated for C_22_H_25_O_5_N_2_BrK 515.0577; found 515.0584.

#### 2-((4-chlorophenyl)amino)-2-oxoethyl (2S)-2-(1,3-dioxo-1,3,3a,4,7,7a-hexahydro-2H-isoindol-2-yl)-4-methylpentanoate (3g)

Methods B and C followed by F

Very viscous liquid, 75% ^1^H NMR (500 MHz, DMSO) δ 10.13 (s, 1H); 7.59 (m, 2H); 7.37 (m, 2H); 5.84 (m, 2H); 4.79 (dd, *J* = 11.2, 4.5 Hz, 1H); 4.67 (m, 2H); 3.24 (m, 2H); 2.39 (m, 2H); 2.20 (dd, *J* = 14.9, 7.4 Hz, 2H); 2.02 (m, 1H); 1.76 (ddd, *J* = 14.2, 10.1, 4.5 Hz, 1H); 1.35 (m, 1H); 0.84 (dd, *J* = 9.8, 6.7 Hz, 6H). ^13^C NMR (125 MHz, DMSO) δ 179.6; 179.5; 168.5; 165.0; 137.2; 128.8; 127.5; 127.4; 127.3; 120.9; 63.4; 50.2; 38.5; 36.0; 24.1; 23.2; 23.0; 20.7.

HRMS (ESI-Orbitrap): [M+H]^+^ Calculated for C_22_H_26_O_5_N_2_Cl 433.1530; found 433.1507.

#### 2-((4-hydroxyphenyl)amino)-2-oxoethyl (2S)-2-(1,3-dioxo-1,3,3a,4,7,7a-hexahydro-2H-isoindol-2-yl)-4-methylpentanoate (3h)

Methods B and C followed by F

Very viscous liquid, 84% ^1^H NMR (250 MHz, DMSO) δ 10.05 (s, 1H); 7.57 (d, *J* = 8.8 Hz, 1H); 6.98 (d, *J* = 8.8 Hz, 1H); 5.80 (m, 2H); 4.88 (m, 1H); 4.66 (m, 1H); 3.24 (m, 1H); 2.40 (m, 2H); 2.19 (m, 2H); 1.98 (m, 1H); 1.77 (m, 1H); 1.35 (m, 1H); 0.85 (m, 6H).

#### 2-(naphthalen-2-ylamino)-2-oxoethyl (2S)-2-(1,3-dioxo-1,3,3a,4,7,7a-hexahydro-2H-isoindol-2-yl)-4-methylpentanoate (3i)

Methods B and C followed by F

Very viscous liquid, 68% ^1^H NMR (250 MHz, DMSO) δ 8.43 (s, 2H); 7.80 (m, 4H); 7.45 (m, 2H); 5.91 (m, 2H); 5.13 (d, *J* = 15.5 Hz, 1H); 4.92 (dd, *J* = 10.8, 4.5 Hz, 1H); 4.47 (d, *J* = 15.5 Hz, 1H); 3.23 (m, 2H); 2.68 (m, 2H); 2.22 (m, 3H); 1.97 (m, 1H); 0.95 (d, *J* = 6.5 Hz, 6H).

HRMS (ESI-Orbitrap): [M+Na]^+^ Calculated for C_26_H_28_O_5_N_2_Na 471.18959; found 471.18888.

#### 2-((4-fluoro-3-nitrophenyl)amino)-2-oxoethyl (2S)-2-(1,3-dioxo-1,3,3a,4,7,7a-hexahydro-2H-isoindol-2-yl)-4-methylpentanoate (3j)

Methods A and C followed by F

Very viscous liquid, 84% ^1^H NMR (400 MHz, CDCl_3_) δ 8.72 (sl, 1H); 8.56 (dd, *J* = 6.6, 2.7 Hz, 1H); 8.20 (m, 1H); 7.27 (m, 1H); 5.92 (m, 2H); 5.07 (d, *J* = 15.8 Hz, 1H); 4.90 (dd, *J* = 10.7, 4.6 Hz, 1H); 4.46 (d, *J* = 15.7 Hz, 1H); 3.26 (m, 2H); 2.67 (m, 2H); 2.31 (m, 2H); 1.99 (m, 2H); 1.41 (m, 1H); 0.93 (d, *J* = 6.4 Hz, 6H), ^13^C NMR (100 MHz, CDCl_3_) δ 180.7; 180.5; 153.1; 150.5; 137.1; 137.0; 134.3; 134.2; 127.8; 127.6; 126.7; 126.6; 118.7; 118.5; 117.00; 116.97; 63.3; 51.1; 39.4; 39.3; 37.4; 29.6; 24.7; 23.6; 23.5; 23.0; 21.0.

HRMS (ESI-Orbitrap): [M+Na]^+^ Calculated for C_22_H_24_O_7_N_3_FNa 484.14095; found 484.14483.

#### 2-((4-((tert-butoxycarbonyl)(methyl)amino)phenyl)amino)-2-oxoethyl (2S)-2-(1,3-dioxo-1,3,3a,4,7,7a-hexahydro-2H-isoindol-2-yl)-4-methylpentanoate (3k)

Methods A and C followed by F

Very viscous liquid, 90% ^1^H NMR (500 MHz, CDCl_3_) δ 7.74 (d, *J* = 8.5 Hz, 2H); 7.21 (d, *J* = 8.5 Hz); 5.90 (m, 2H); 5.05 (d, *J* = 15.9 Hz, 1H); 4.88 (dd, *J* = 11.0, 4.3 Hz, 1H); 4.41 (d, *J* = 15.3 Hz, 1H); 3.24 (s, 3H); 2.65 (m, 2H); 2.27 (m, 2H); 2.10 (m, 1H); 1.95 (m, 1H); 1.45 (s, 9H); 0.92 (d, *J* = 6.71 Hz, 6H), ^13^C NMR (125 MHz, CDCl_3_) δ 180.4; 180.3; 168.1; 164.8; 154.8; 140.2; 134.7; 127.72; 127.68; 125.9; 120.1; 80.2; 63.4; 51.0; 39.3; 39.2; 37.3; 37.2; 28.3; 24.7; 23.6; 23.5; 23.0; 20.9.

HRMS (ESI-Orbitrap): [M+Na]^+^ Calculated for C_28_H_37_O_7_N_3_Na 550.25237; found 550.25177.

#### 2-((4-nitrophenyl)amino)-2-oxoethyl (2S)-2-(1,3-dioxo-1,3,3a,4,7,7a-hexahydro-2H-isoindol-2-yl)-4-methylpentanoate (3l)

Methods A and C followed by F

Very viscous liquid, 89% ^1^H NMR (600 MHz, CDCl_3_) δ 8.72 (sl, 1H); 8.24 (m, 2H); 8.03 (m, 2H); 5.91 (m, 2H); 5.10 (d, *J* = 15.8 Hz, 1H); 4.90 (dd, *J* = 11.0, 4.4Hz, 1H); 3.25 (m, 2H); 2.66 (m, 2H); 2.29 (m, 2H); 2.06 (m, 1H); 1.96 (m, 1H); 1.41 (m, 1H); 0.93 (d, *J* = 6.6 Hz, 6H), ^13^C NMR (150 MHz, CDCl_3_) δ 180.6; 180.5; 168.1; 165.7; 143.9; 143.4; 127.8; 127.7; 124.9; 119.6; 63.4; 51.1; 39.37; 39.35; 37.4; 24.7; 23.6; 23.5; 23.0; 21.0.

HRMS (ESI-Orbitrap): [M+Na]^+^ Calculated for C_22_H_25_O_7_N_3_Na 466.15902; found 466.15749.

#### 2-((4-methoxy-2-nitrophenyl)amino)-2-oxoethyl (2S)-2-(1,3-dioxo-1,3,3a,4,7,7a-hexahydro-2H-isoindol-2-yl)-4-methylpentanoate (3m)

Methods A and C followed by F

Very viscous liquid, 98% ^1^H NMR (500 MHz, CDCl_3_) δ 10.51 (sl, 1H); 8.57 (d, *J* = 9.15 Hz, 1H); 7.66 (d, *J* = 2.9 Hz, 1H); 7.24 (dd, *J* = 9.3, 3.0 Hz, 1H); 5.92 (m, 2H); 4.95 (dd, *J* = 11.3, 4.4 Hz, 1H); 4.77 (m, 2H); 3.87 (s, 3H); 3.31 (m, 1H); 3.18 (m, 1H); 2.62 (m, 2H); 2.26 (m, 2H); 2.16 (m, 1H); 1.97 (m, 1H); 1.39 (m, 1H); 0.93 (dd, *J* = 6.6, 4.5 Hz, 6H), ^13^C NMR (125 MHz, CDCl_3_) δ 179.5; 179.43; 168.0; 165.0; 155.5; 137.7; 127.7; 127.6; 126.6; 124.0; 122.8; 108.8; 64.0; 55.8; 50.9; 39.2; 38.9; 36.7; 24.6; 23.6; 23.4; 23.0; 20.77.

HRMS (ESI-Orbitrap): [M+Na]^+^ Calculated for C_23_H_28_O_8_N_3_ 474.19045; found 474.19131.

#### 2-((3-chloro-4-methoxyphenyl)amino)-2-oxoethyl (S)-2-(1,3-dioxoisoindolin-2-yl)-4-methylpentanoate (10a)

Methods A and C followed by F

Very viscous liquid, 82% ^1^H NMR (250 MHz, DMSO) δ 10.04 (sl, 1H); 7.92 (m, 4H); 7.69 (d, *J* = 2.4 Hz, 1H); 7.37 (dd, *J* = 8.9, 2.4 Hz, 1H); 7.10 (d, *J* = 9.0 Hz, 1H); 5.04 (dd, *J* = 11.2, 4.4 Hz, 1H); 4.72 (m, 2H); 3.81 (s, 3H); 2.24 (m, 1H); 1.93 (ddd, *J* = 14.0, 9.9, 4.5 Hz, 1H); 1.49 (m, 1H); 0.89 (m, 6H). ^13^C NMR (62.5 MHz, DMSO) δ 169.1; 167.2; 164.6; 150.7; 135.0; 131.9; 131.0; 123.5; 121.0; 120.6; 119.2; 112.9; 63.4; 56.1; 49.9; 36.6; 24.5; 22.9; 20.8.

HRMS (ESI-Orbitrap): [M+H]^+^ Calculated for C_23_H_24_O_6_N_2_Cl 459.1323; found 459.1355.

#### 2-((3-chloro-4-methoxyphenyl)amino)-2-oxoethyl (2S)-2-(1,3-dioxooctahydro-2H-isoindol-2-yl)-4-methylpentanoate (10b)

Methods A and C followed by F

Very viscous liquid, 91% ^1^H NMR (500 MHz, CDCl_3_) δ 8.32 (sl, 1H); 7.83 (d, *J* = 2.5 Hz, 1H); 7.68 (dd, *J* = 8.9, 2.6 Hz, 1H); 6.90 (d, *J* = 8.9 Hz, 1H); 5.08 (d, *J* = 15.6 Hz, 1H); 4.90 (dd, *J* = 10.9, 4.3 Hz, 1H); 4.45 (d, *J* = 15.6 Hz, 1H); 3.89 (s, 3H); 2.12 (ddd, *J* = 14.5, 10.7, 4.3 Hz, 1H); 1.98 (m, 2H); 1.85 (m, 2H); 1.77 (m, 1H); 1.47 (m, 4H); 1.36 (m, 1H); 0.96 (m, 6H). ^13^C NMR (100 MHz, CDCl_3_) δ 180.3; 179.4; 168.4; 164.7; 152.0; 131.1; 122.3; 119.5; 112.1; 63.5; 56.3; 50.5; 40.0; 39.8; 37.4; 25.0; 24.0; 23.3; 23.1; 21.6; 21.1.

HRMS (ESI-Orbitrap): [M+Na]^+^ Calculated for C_23_H_29_O_6_N_2_ClNa 487.19118; found 487.16025.

#### 2-((3-chloro-4-methoxyphenyl)amino)-2-oxoethyl (S)-2-(2,5-dioxo-2,5-dihydro-1H-pyrrol-1-yl)-4-methylpentanoate (10c)

Methods A and D followed by F

Very viscous liquid, 46% ^1^H NMR (400 MHz, CDCl_3_) δ 8.12 (sl, 1H); 7.76 (d, *J* = 2.6 Hz, 1H); 7.62 (dd, *J* = 8.9, 2.6 Hz, 1H); 6.90 (d, *J* = 8.9 Hz, 1H); 6.84 (s, 2H); 5.04 (d, *J* = 15.5 Hz, 1H); 4.91 (dd, *J* = 11.4, 4.4 Hz, 1H); 4.47 (d, *J* = 15.5 Hz, 1H); 3.89 (s, 3H); 2.18 (ddd, *J* = 14.3, 11.2, 4.3 Hz, 1H); 1.98 (ddd, *J* = 14.3, 10.0, 4.3 Hz, 1H); 1.47 (m, 1H); 0.96 (dd, *J* = 6.6, 1.2 Hz, 6H). ^13^C NMR (100 MHz, CDCl_3_) δ 170.4; 168.4; 164.6; 152.1; 134.6; 130.9; 122.5; 122.4; 63.6; 56.3; 50.4; 38.0; 24.9; 23.0; 21.0.

#### 2-((3-chloro-4-methoxyphenyl)amino)-2-oxoethyl (S)-2-(2,5-dioxopyrrolidin-1-yl)-4-methylpentanoate (10d)

Methods A and D followed by F

Very viscous liquid, 72% ^1^H NMR (400 MHz, CDCl_3_) δ 8.30 (sl, 1H); 7.79 (d, *J* = 2.6 Hz, 1H); 7.62 (dd, *J* = 8.9, 2.7 Hz, 1H); 6.89 (d, *J* = 8.9 Hz, 1H); 5.01 (d, *J* = 15.4 Hz, 1H); 4.94 (dd, *J* = 10.5, 4.8 Hz, 1H); 4.49 (d, *J* = 15.5 Hz, 1H); 3.88 (s, 3H); 2.86 (m, 4H); 2.05 (m, 2H); 1.45 (m, 1H); 0.96 (dd, *J* = 6.7, 4.6 Hz, 6H), ^13^C NMR (100 MHz, CDCl_3_) δ 177.2; 168.2; 164.7; 152.0; 131.0; 122.3; 119.6; 112.1; 63.4; 56.3; 50.9; 37.3; 28.2; 24.9; 22.9; 21.1.

HRMS (ESI-Orbitrap): [M+Na]^+^ Calculated for C_19_H_23_O_6_N_2_ClNa 433.11423; found 433.11424.

#### 2-((3-chloro-4-methoxyphenyl)amino)-2-oxoethyl (S)-4-methyl-2-(3-methyl-2,5-dioxo-2,5-dihydro-1H-pyrrol-1-yl)pentanoate (10e)

Methods A and D followed by F

Very viscous liquid, 88% ^1^H NMR (500 MHz, CDCl_3_) δ 8.19 (sl, 1H); 7.78 (d, *J* = 2.5 Hz, 1H); 7.65 (dd, *J* = 8.9, 2.6 Hz, 1H); 6.90 (d, *J* = 9.0 Hz, 1H); 6.46 (d, *J* = 1.9 Hz, 1H); 5.04 (d, *J* = 15.6 Hz, 1H); 4.89 (dd, *J* = 11.4, 4.3 Hz, 1H); 4.45 (d, *J* = 15.6 Hz, 1H); 3.89 (s, 3H); 2.17 (m, 4H); 1.96 (m, 1H); 1.47 (m, 1H); 0.95 (dd, *J* = 6.7, 1.7 Hz, 6H), ^13^C NMR (125 MHz, CDCl_3_) δ 171.6; 170.5; 168.6; 164.6; 152.0; 146.3; 130.9; 127.8; 122.4; 122.3; 119.5; 112.1; 63.5; 56.3; 50.5; 38.1; 24.9; 23.0; 20.9; 11.2.

HRMS (ESI-Orbitrap): [M+Na]^+^ Calculated for C_20_H_23_O_6_N_2_ClNa 445.11369; found 445.11261.

#### 2-((3-chloro-4-methoxyphenyl)amino)-2-oxoethyl (S)-2-(3,4-dichloro-2,5-dioxo-2,5-dihydro-1H-pyrrol-1-yl)-4-methylpentanoate (10f)

Methods A and D followed by F

Very viscous liquid, 27% ^1^H NMR (500 MHz, CDCl_3_) δ 7.85 (sl, 1H); 7.74 (d, *J* = 2.5 Hz, 1H); 7.59 (dd, *J* = 9.0, 2.5 Hz, 1H); 6.91 (d, *J* = 9.0 Hz, 1H); 5.02 (d, *J* = 15.4, 1H); 4.98 (dd, *J* = 11.4, 4.5 Hz, 1H); 4.52 (d, *J* = 15.4 Hz, 1H); 3.90 (s, 3H); 2.21 (m, 1H); 2.00 (m, 1H); 1.51 (m, 1H); 0.97 (dd, *J* = 6.5, 4.3 Hz, 6H), ^13^C NMR (125 MHz, CDCl_3_) δ 167.6; 164.1; 163.0; 152.3; 133.9; 130.6; 122.6; 122.4; 119.6; 112.2; 63.9; 56.4; 51.9; 37.9; 25.0; 23.0; 20.9.

HRMS (ESI-Orbitrap): [M+Na]^+^ Calculated for C_19_H_19_O_6_N_2_Cl_3_Na 499.02009; found 499.02014.

#### 2-((3-chloro-4-methoxyphenyl)amino)-2-oxoethyl 2-(1,3-dioxo-1,3,3a,4,7,7a-hexahydro-2H-isoindol-2-yl)acetate (10g)

Methods A and C followed by F

Very viscous liquid, 80% ^1^H NMR (500 MHz, DMSO) δ 12.40 (sl, 1H); 9.89 (s, 1H); 7.75 (d, *J* = 2.6 Hz, 1H); 7.43 (dd, *J* = 8.9, 2.6 Hz, 1H); 7.11 (d, *J* = 9.0 Hz, 1H); 5.64 (m, 2H); 4.62 (m, 2H); 3.82 (s, 3H); 3.07 (m, 2H); 2.46 (m, 2H); 2.33 (m, 2H), ^13^C NMR (125 MHz, DMSO) δ 174.6; 172.6; 165.3; 150.7; 132.0; 125.3; 124.9; 121.1; 120.6; 119.3; 112.9; 62.5; 56.2; 38.8; 38.6; 25.6; 25.2.

#### 2-((3-chloro-4-methoxyphenyl)amino)-2-oxoethyl (2S)-2-(1,3-dioxo-1,3,3a,4,7,7a-hexahydro-2H-isoindol-2-yl)-3-phenylpropanoate (10h)

Methods A and D followed by F

Very viscous liquid, 87% ^1^H NMR (250 MHz, CDCl_3_) δ 8.26 (sl, 1H); 7.83 (d, *J* = 2.7 Hz, 1H); 7.71 (dd, *J* = 8.9, 2.6 Hz, 1H); 7.22 (m, 5H); 6.92 (d, *J* = 8.8 Hz, 1H); 5.70 (m, 2H); 5.12 (m, 2H); 4.42 (d, *J* = 15.5 Hz, 1H); 3.90 (s, 3H); 3.56 (m, 1H); 3.36 (dd, *J* = 14.3, 11.3 Hz, 1H); 2.99 (m, 2H); 2.48 (ddd, *J* = 15.8, 6.0, 2.9 Hz, 1H); 2.22 (m, 3H).

HRMS (ESI-Orbitrap): [M+Na]^+^ Calculated for C_26_H_25_O_6_N_2_ClNa 519.12988; found 519.12918.

${\left[\alpha \right]}_{\text{D}}^{20}-$24,0 (1, MeOH).

HPLC flow 3.5 mL/min, polarity hexane:ethyl acetate (60:40). Rt 16.76 min, area 97.10%; Rt 20.09 min, area 2.90%.

#### 2-((3-chloro-4-methoxyphenyl)amino)-2-oxoethyl 2-(1,3-dioxo-1,3,3a,4,7,7a-hexahydro-2H-isoindol-2-yl)-3-phenylpropanoate (10h_rac_)

Methods A and D followed by F

Very viscous liquid, 81% ^1^H NMR (500 MHz, CDCl_3_) δ 8.29 (sl, 1H); 7.84 (d, *J* = 2.2 Hz, 1H); 7.69 (dd, *J* = 8.8, 2.4 Hz, 1H); 7.24 (m, 3H); 7.13 (d, *J* = 7.1 Hz, 2H); 6.92 (d, *J* = 9.0 Hz, 1H); 5.70 (m, 2H); 5.12 (m, 2H); 4.43 (d, *J* = 15.6 Hz, 1H); 3.90 (s, 3H); 3.56 (dd, *J* = 14.3, 5.2 Hz, 1H); 3.35 (dd, *J* = 14.2, 11.5 Hz, 1H); 3.04 (m, 1H); 2.93 (m, 1H); 2.48 (ddd, *J* = 15.7, 6.05, 2.6 Hz, 1H); 2.29 (ddd, *J* = 15.8, 5.9, 3.0 Hz, 1H); 2.17 (m, 2H), ^13^C NMR (125 MHz, CDCl_3_) δ 180.2; 179.8; 167.3; 164.6; 152.0; 135.3; 131.0; 129.0; 128.6; 127.4; 127.21; 127.17; 122.33; 122.28; 119.5; 112.1; 63.4; 56.3; 53.2; 39.0; 38.9; 34.3; 23.11; 23.07.

HRMS (ESI-Orbitrap): [M+Na]^+^ Calculated for C_26_H_25_O_6_N_2_ClNa 519.12988; found 519.12965.

${\left[\alpha \right]}_{\text{D}}^{20}$ 0 (1, MeOH).

HPLC flow 3.5 mL/min, polarity hexane:ethyl acetate (60:40). Rt 17.16 min, area 50.19%; Rt 20.49 min, area 49.81%.

#### 2-((3-chloro-4-methoxyphenyl)amino)-2-oxoethyl (2R)-2-(1,3-dioxo-1,3,3a,4,7,7a-hexahydro-2H-isoindol-2-yl)-3-(naphthalen-2-yl)propanoate (10i)

Methods A and D followed by F

Very viscous liquid, 43% (for 2 steps) ^1^H NMR (400 MHz, CDCl_3_) δ 8.29 (sl, 1H); 7.85 (d, *J* = 2.7 Hz, 1H); 7.77 (m, 3H); 7.71 (dd, *J* = 8.9, 2.6 Hz, 1H); 7.54 (m, 1H); 7.46 (m, 2H); 7.29 (dd, *J* = 8.4, 1.8 Hz, 1H); 6.93 (d, *J* = 8.9 Hz, 1H); 5.48 (m, 2H); 5.26 (dd, *J* = 11.4, 5.4 Hz, 1H); 5.14 (d, *J* = 15.4 Hz, 1H); 4.44 (d, *J* = 15.4 Hz, 1H); 3.72 (dd, *J* = 14.3, 5.3 Hz, 1H); 3.54 (dd, *J* = 14.4, 11.4 Hz, 1H); 3.00 (ddd, *J* = 9.0, 7.6, 3.0 Hz, 1H); 2.87 (ddd, *J* = 9.0, 7.8; 2.7 Hz, 1H); 2.46 (ddd, *J* = 15.8, 6.4, 2.6 Hz, 1H); 2.22 (ddd, *J* = 15.8, 6.4, 3.0 Hz, 1H); 2.08 (m, 2H), ^13^C NMR (100 MHz, CDCl_3_) δ 180.2; 180.0; 167.4; 164.7; 152.1; 133.3; 132.8; 132.5; 131.1; 128.5; 128.1; 127.7; 127.4; 127.2; 127.0; 126.6; 126.3; 125.9; 122.5; 122.4; 119.6; 112.2; 63.5; 56.4; 53.2; 39.0; 38.9; 34.6; 23.1.

HRMS (ESI-Orbitrap): [M+Na]^+^ Calculated for C_30_H_27_O_6_N_2_ClNa 569.14553; found 569.14428.

#### 2-((3-chloro-4-methoxyphenyl)amino)-2-oxoethyl (S)-2-(2,5-dioxo-2,5-dihydro-1H-pyrrol-1-yl)-3-phenylpropanoate (10j)

Methods A and D followed by F

Very viscous liquid, 23% ^1^H NMR (500 MHz, CDCl_3_) δ 8.17 (sl, 1H); 7.76 (d, *J* = 2.6 Hz, 1H); 7.66 (dd, *J* = 8.9, 2.6 Hz, 1H); 7.25 (m, 3H); 7.13 (m, 2H); 6.92 (d, *J* = 8.9 Hz, 1H); 6.69 (s, 2H); 5.10 (m, 2H); 4.50 (d, *J* = 15.4 Hz, 1H); 3.90 (s, 3H); 3.57 (dd, *J* = 14.2, 5.2 Hz, 1H); 3.38 (dd, *J* = 14.2, 11.2 Hz, 1H), ^13^C NMR (125 MHz, CDCl_3_) δ 170.1; 167.6; 164.5; 152.2; 135.5; 134.3; 130.9; 128.9; 128.8; 127.4; 122.5; 119.7; 112.2; 63.7; 56.4; 53.2; 35.4.

HRMS (ESI-Orbitrap): [M+Na]^+^ Calculated for C_22_H_19_O_6_N_2_ClNa 465.08239; found 465.08244.

#### 2-((3-chloro-4-methoxyphenyl)amino)-2-oxoethyl (S)-4-methyl-2-(pyrrolidin-1-yl)pentanoate (10k)

Methods A and E followed by F

Very viscous liquid, 93% ^1^H NMR (500 MHz, MeOD) δ 8.66 (sl, 1H); 7.57 (d, *J* = 2.7 Hz, 1H); 7.46 (dd, *J* = 8.8, 2.5 Hz, 1H); 6.88 (d, *J* = 9.0 Hz, 1H); 4.73 (m, 2H); 3.88 (s, 1H); 3.35 (dd, *J* = 9.9, 4.6 Hz, 1H); 2.73 (m, 2H); 2.63 (m, 2H); 1.80 (m, 5H); 1.58 (m, 2H); 0.96 (dd, *J* = 8.5, 6.1 Hz, 6H), ^13^C NMR (125 MHz, MeOD) δ 171.3; 165.3; 152.2; 130.6; 122.7; 122.4; 120.0; 112.1; 65.1; 62.7; 56.3; 50.6; 39.0; 25.4; 23.4; 23.3; 21.9.

#### 2-((3-chloro-4-methoxyphenyl)amino)-2-oxoethyl (S)-4-methyl-2-(piperidin-1-yl)pentanoate (10l)

Methods A and E followed by F

Very viscous liquid, 87% ^1^H NMR (500 MHz, CDCl_3_) δ 8.05 (sl, 1H); 7.56 (d, *J* = 2.7 Hz, 1H); 7.42 (dd, *J* = 8.9, 2.6 Hz, 1H); 6.90 (d, *J* = 8.8 Hz, 1H); 4.83 (d, *J* = 15.6 Hz, 1H); 4.63 (d, *J* = 15.6 Hz, 1H); 3.82 (s, 3H); 3.37 (dd, *J* = 8.8, 5.7 Hz, 1H); 2.59 (m, 4H); 1.60 (m, 7H); 1.44 (m, 2H); 0.94 (dd, *J* = 9.1, 6.3 Hz, 6H), ^13^C NMR (125 MHz, CDCl_3_) δ 170.8; 165.3; 152.4; 130.2; 123.0; 122.6; 120.1; 112.2; 66.5; 62.4; 56.4; 50.9; 36.6; 26.4; 25.3; 24.4; 22.8; 22.4.

HRMS (ESI-Orbitrap): [M+Na]^+^ Calculated for C_20_H_29_O_4_N_2_ClNa 419.17081; found 419.17050.

#### 2-((3-chloro-4-methoxyphenyl)amino)-2-oxoethyl (S)-4-methyl-2-morpholinopentanoate (10m)

Methods A and E followed by F

Very viscous liquid, 75% ^1^H NMR (500 MHz, CDCl_3_) δ 7.96 (sl, 1H); 7.56 (d, *J* = 2.6 Hz, 1H); 7.38 (dd, *J* = 8.8, 2.6 Hz, 1H); 6.89 (d, *J* = 8.9 Hz, 1H); 4.76 (d, *J* = 15.3 Hz, 1H); 4.68 (d, *J* = 15.3 Hz, 1H); 3.88 (s, 3H); 3.68 (m, 4H); 3.40 (t, *J* = 7.3 Hz, 1H); 2.67 (m, 4H); 1.65 (m, 3H); 0.96 (dd, *J* = 8.9, 6.1 Hz, 6H), ^13^C NMR (125 MHz, CDCl_3_) δ 170.8; 165.0; 152.4; 130.1; 122.8; 122.6; 120.0; 112.2; 67.2; 65.8; 62.6; 56.3; 49.8; 36.8; 25.0; 22.6; 22.4.

HRMS (ESI-Orbitrap): [M+Na]^+^ Calculated for C_19_H_27_O_5_N_2_ClNa 421.15007; found 421.14966.

#### tert-butyl (S)-4-(1-(2-((3-chloro-4-methoxyphenyl)amino)-2-oxoethoxy)-4-methyl-1-oxopentan-2-yl)piperazine-1-carboxylate (10n)

Methods A and E followed by F

Very viscous liquid, 69% ^1^H NMR (500 MHz, CDCl_3_) δ 7.86 (sl, 1H); 7.56 (d, *J* = 2.4 Hz, 1H); 7.37 (dd, *J* = 8.9, 2.4 Hz, 1H); 6.89 (d, *J* = 9.0 Hz, 1H); 4.74 (d, *J* = 15.3 Hz, 1H); 4.67 (d, *J* = 15.3 Hz, 1H); 3.89 (s, 3H); 3.43 (m, 5H); 2.62 (m, 4H); 1.65 (m, 3H); 1.44 (s, 9H); 0.95 (dd, *J* = 12.3, 5.9 Hz, 6H), ^13^C NMR (125 MHz, CDCl_3_) δ 170.7; 164.9; 154.3; 152.4; 130.1; 122.8; 122.7; 120.0; 112.3; 79.8; 65.5; 62.6; 56.4; 49.3; 37.1; 28.4; 25.0; 22.5.

HRMS (ESI-Orbitrap): [M+Na]^+^ Calculated for C_24_H_36_O_6_N_3_ClNa 520.21848; found 520.21832.

### General Procedure for the Preparation of Compounds 3k' and 10o

The esters, obtained through methods A and D or E followed by F, were dissolved in dry DCM, and a large excess of HCl (4 M solution in dioxane) was added. The resulting solution was stirred at room temperature for 4 h. The solvent mixture was removed under reduced pressure, and the residue was dissolved in DCM followed by crystallization from hexane.

#### 2-((4-(methylamino)phenyl)amino)-2-oxoethyl 2-(1,3-dioxo-1,3,3a,4,7,7a-hexahydro-2H-isoindol-2-yl)-4-methylpentanoate (3k')

Very viscous liquid, 75% ^1^H NMR (500 MHz, CDCl_3_) δ 8.04 (sl, 1H); 7.53 (d, *J* = 8.8 Hz, 2H); 6.60 (d, *J* = 8.8 Hz, 2H); 5.89 (m, 2H); 5.02 (d, *J* = 15.3 Hz, 1H); 4.87 (dd, *J* = 11.1, 4.3 Hz, 1H); 4.40 (d, *J* = 15.4 Hz, 1H); 3.72 (sl, 1H); 3.20 (m, 2H); 2.83 (s, 3H); 2.64 (m, 2H); 2.25 (m, 2H); 2.11 (m, 1H); 1.94 (m, 1H); 1.40 (m, 1H); 0.92 (d, *J* = 6.6 Hz, 6H), ^13^C NMR (125 MHz, CDCl_3_) δ 180.3; 180.1; 168.0; 164.3; 146.6; 127.7; 121.7; 112.5; 63.5; 51.1; 39.2; 37.1; 31.0; 24.8; 23.55; 23.48; 23.0; 20.9.

HRMS (ESI-Orbitrap): [M+Na]^+^ Calculated for C_23_H_29_O_5_N_3_FNa 450.19994; found 450.19913.

#### 2-((3-chloro-4-methoxyphenyl)amino)-2-oxoethyl (S)-4-methyl-2-(piperazin-1-yl)pentanoate hydrochloride (10o)

Amorphous solid, 96% ^1^H NMR (500 MHz, MeOD) δ 7.67 (m, 1H); 7.38 (m, 1H); 7.02 (m, 1H); 4.83 (m, 2H); 3.98 (m, 1H); 3.85 (s, 3H); 3.78 (m, 1H); 3.46 (m, 6H); 3.27 (m, 1H); 1.82 (m, 3H); 1.00 (dd, *J* = 11.1, 6.2 Hz, 6H), ^13^C NMR (125 MHz, CDCl_3_) δ 172.3; 170.5; 167.3; 167.2; 156.2; 153.9; 132.7; 123.9; 123.4; 121.4; 114.1; 74.4; 68.3; 66.9; 64.5; 64.4; 57.2; 47.8; 44.5; 44.0; 41.0; 38.2; 26.4; 26.1; 23.4; 23.1; 22.4; 22.2.

HRMS (ESI-Orbitrap): [M+H]^+^ Calculated for C_19_H_28_O_4_N_3_Cl 398.18466; found 398.18384.

### General Procedure for the Preparation of Compounds 3l' and 3m'

The esters, obtained through methods A and D followed by F, were dissolved in methanol, followed by the addition of the catalyst Pd/C (10 wt%, 0.1 equivalents). The mixture stirred at room temperature under a hydrogen atmosphere (1 atm) for 4 h and then filtered through Celite®. The filtrate was concentrated in vacuum. The product was purified by column chromatography on silica gel using a mixture of ethyl acetate/hexanes (50%) as the eluent.

#### 2-((4-aminophenyl)amino)-2-oxoethyl 2-(1,3-dioxo-1,3,3a,4,7,7a-hexahydro-2H-isoindol-2-yl)-4-methylpentanoate (3l')

Pale amorphous solid, 92% ^1^H NMR (500 MHz, CDCl_3_) δ 8.06 (sl, 1H); 7.51 (d, *J* = 8.7 Hz, 2H); 6.67 (d, *J* = 8.7 Hz, 2H); 5.89 (m, 2H); 5.03 (d, *J* = 15.4 Hz, 1H) 4.87 (dd, *J* = 11.0, 4.3 Hz, 1H); 4.40 (d, *J* = 15.4 Hz, 1H); 3.65 (sl, 2H); 3.20 (m, 2H); 2.64 (m, 2H); 2.26 (m, 2H); 2.10 (m, 1H); 1.94 (m, 1H); 1.40 (m, 1H); 0.92 (d, *J* = 6.6 Hz, 6H). ^13^C NMR (125 MHz, CDCl_3_) δ 180.4; 180.2; 168.1; 164.4; 143.4; 128.9; 127.7; 121.7; 115.3; 63.5; 51.1; 39.3; 37.1; 24.8; 23.58; 23.50; 23.1; 21.0.

HRMS (ESI-Orbitrap): [M+Na]^+^ Calculated for C_22_H_27_O_5_N_3_Na 436.18484; found 436.18421.

#### 2-((2-amino-4-methoxyphenyl)amino)-2-oxoethyl 2-(1,3-dioxo-1,3,3a,4,7,7a-hexahydro-2H-isoindol-2-yl)-4-methylpentanoate (3m')

Light brown amorphous solid, 94% ^1^H NMR (500 MHz, CDCl_3_) δ 7.90 (sl, 1H); 7.08 (d, *J* = 8.5 Hz, 1H); 6.34 (m, 2H); 5.90 (m, 2H); 4.98 (d, *J* = 15.6 Hz, 1H); 4.87 (dd, *J* = 11.0, 4.3 Hz, 1H); 4.54 (d *J* = 15.4 Hz, 1H); 3.75 (s, 3H); 3.18 (m, 2H); 2.61 (m, 2H); 2.23 (m, 2H); 2.07 (m, 1H); 1.93 (m, 1H); 1.37 (m, 1H); 0.91 (dd, *J* = 6.6, 3.4 Hz, 6H). ^13^C NMR (125 MHz, CDCl_3_) δ 180.12; 180.09; 168.5; 165.6; 159.3; 142.8; 127.71; 127.71; 127.26; 115.5; 104.5; 102.6; 63.8; 55.3; 51.0; 39.21; 39.15; 37.1; 24.7; 23.50; 23.48; 23.0; 20.9.

HRMS (ESI-Orbitrap): [M+Na]^+^ Calculated for C_23_H_30_O_6_N_3_ 444.21627; found 444.21738.

### Procedure for the Preparation of N-(2-((3-chloro-4-methoxyphenyl)amino)-2-oxoethyl)-2-(1,3-dioxo-1,3,3a,4,7,7a-hexahydro-2H-isoindol-2-yl)-4-methylpentanamide (5)

2-(1,3-Dioxo-1,3,3a,4,7,7a-hexahydro-2*H*-isoindol-2-yl)-4-methylpentanoic acid **(1)** was dissolved in dichloromethane at room temperature. Oxalyl chloride (1.5 equivalents) and 0.1 mL of dimethyl formamide (DMF) were added to this solution as the catalyst. After 2 h, the solvent was removed under vacuum. The residue was dissolved in dichloromethane, and glycine (1.0 equivalents) was added, followed by the addition of triethylamine (2.2 equivalents).

The isolated product from the previous step was dissolved in dichloromethane at room temperature. Oxalyl chloride (1.5 equivalents) and 0.1 mL dimethyl formamide (DMF) were added to this solution as the catalyst. After 2 h, the solvent was removed under vacuum. The residue was dissolved in dichloromethane, and 3-chloro-4-methoxyaniline (1.0 equivalents) was added, followed by the addition of triethylamine (2.2 equivalents). The reaction stirred for an additional 2 h. The reaction mixture was washed with water and saturated aqueous NH_4_Cl solution. The organic phase was dried over MgSO_4_ and the solvent was removed under reduced pressure. The product was purified by column chromatography on silica gel using a mixture of ethyl acetate/hexanes (50%) as the eluent.

Amorphous solid, 78% ^1^H NMR (250 MHz, DMSO) δ 9.68 (sl, 1H); 7.64 (d, *J* = 2.4 Hz, 1H); 7.39 (dd, *J* = 8.9, 2.4 Hz, 1H); 7.09 (d, *J* = 9.0 Hz, 1H); 5.86 (m, 2H) 4.65 (dd, *J* = 11.3, 4.3 Hz, 1H); 3.81 (sl, 3H); 3.18 (m, 2H); 2.39 (m, 2H); 2.11 (m, 3H); 1.79 (m, 1H); 1.29 (m, 1H); 0.83 (m, 6H). ^13^C NMR (62.5 MHz, DMSO) δ 180.1; 179.8; 166.8; 150.8; 132.1; 127.7; 127.6; 122.0; 120.4; 120.2; 112.7; 56.2; 52.4; 36.2; 24.3; 23.4; 23.3; 23.1; 20.6.

### Procedure for the Preparation of 2-((3-chloro-4-methoxyphenyl)amino)ethyl 2-(1,3-dioxo-1,3,3a,4,7,7a-hexahydro-2H-isoindol-2-yl)-4-methylpentanoate (7)

A solution of compound **1** in dry DCM was prepared, and alcohol **6** was added (1.1 equivalents). The solution was cooled in an ice bath followed by the addition of EDC (1.2 equivalents) and DMAP (0.1 equivalents). The solution was allowed to warm to room temperature and stirred for 4 h while monitoring periodically by TLC. The reaction mixture was washed with saturated aqueous NH_4_Cl solution and brine. The organic phase was dried over MgSO_4_ and concentrated under reduced pressure. The product was purified by column chromatography on silica gel using a mixture of ethyl acetate/hexanes (50%) as the eluent.

Pale oil, 62% ^1^H NMR (500 MHz, CDCl_3_) δ 6.82 (d, *J* = 8.7 Hz, 1H); 6.70 (d, *J* = 2.8 Hz, 1H); 6.51 (dd, *J* = 8.8, 2.8 Hz, 1H); 5.89 (m, 1H); 5.82 (m, 1H); 4.76 (dd, *J* = 11.3, 4.3 Hz, 1H); 4.43 (ddd, *J* = 11.1, 7.1, 3.9 Hz, 1H); 4.18 (m, 1H); 3.82 (m, 3H); 3.30 (m, 2H); 3.10 (m, 2H); 2.60 (m, 2H); 2.23 (m, 2H); 2.09 (m, 1H); 1.84 (m, 1H); 1.35 (m, 1H); 0.89 (dd, *J* = 8.8, 6.5 Hz, 6H). ^13^C NMR (125 MHz, CDCl_3_) δ 179.64; 179.61; 169.1; 147.6; 142.5; 127.7; 127.6; 123.4; 115.2; 114.2; 112.1; 64.0; 57.0; 51.2; 43.1; 39.0; 38.9; 36.7; 24.8; 23.43; 23.42; 23.1; 21.0. HRMS (ESI-Orbitrap) m/z [M + Na] calculated for C_23_H_29_O_5_N_2_ClNa 471.16572; found 471.16522.

## Results and Discussion

**Figure 1:**
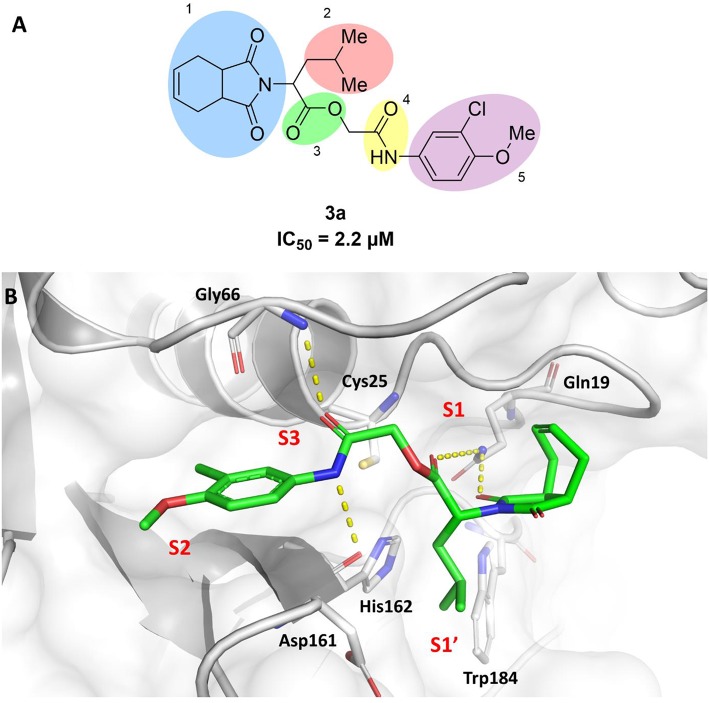
**(A)** Compound **3a** was used as the initial hit for the design of imide derivatives as novel cruzain inhibitors with anti-T. cruzi activity. **(B)** Molecular docking-predicted binding conformation of compound **3a** within the active site of cruzain. Cruzain (PDB 3KKU, 1.28 Å) is depicted in cartoon and surface representations. Binding site residues (carbon in gray) and compound **3a** (carbon in green) are shown as sticks. Hydrogen bonds are shown as dashed lines. Cruzain subsites are labeled as S1, S1', S2, and S3.

In this work, we report the discovery of a series of reversible cruzain inhibitors showing promising trypanocidal activity and low toxicity. The imide derivative **3a** (Figure 1A), a reversible cruzain inhibitor that was previously identified from a virtual high-throughput screening (HTS) combined approach (Ferreira et al., 2010), was taken as the initial hit for molecular optimization. The SBDD strategy relied on the molecular docking-predicted binding mode of **3a** within the catalytic site of cruzain (Figure 1B). The enzyme-inhibitor interactions rely mainly on a hydrogen bonding network. The secondary amide oxygen fills the S3 subsite and interacts with the main chain nitrogen of Gly66. The secondary amide nitrogen in turn forms a hydrogen bond with the main chain carbonyl of Asp161, which lies at the interface between the S2 and S1' subsites. The ester carbonyl projects into the S1 subsite where it engages in a hydrogen bond with the side chain nitrogen of Gln19. One of the imide carbonyl oxygens forms a hydrogen bond with the side chain nitrogen of Gln19 at the S1 subsite. The 3-chloro-4-methoxyphenyl ring projects into the S2 subsite and the isobutyl fragment lies in S1'. Based on these findings, compound **3a** was divided into five fragments that underwent the molecular modifications that eventually led to the series of cruzain inhibitors described herein. The data gathered from the designed analogs were used to further improve the potency against both cruzain and *T. cruzi* and disclose the structure-activity relationships (SAR) enclosed in this dataset.

### Synthesis of Imide Derivatives

**Scheme 1:**
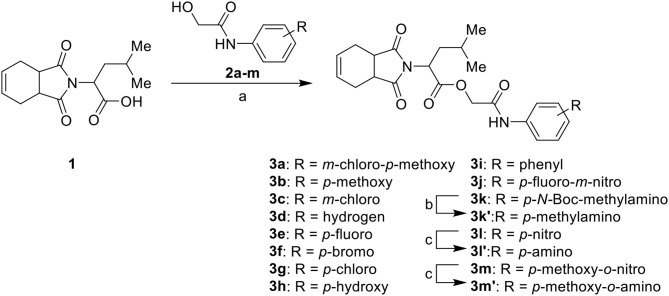
Synthesis of initial hit **3a** and analogs with modifications to the aromatic ring. Reagents and conditions: (a) CH_2_Cl_2_, **1**, EDC, DMAP (cat), rt, 4 h; (b) CH_2_Cl_2_, HCl (4 M in 1,4-dioxane), rt, 4 h; (c) MeOH, H_2_ 1 atm, 10% Pd/C, rt, 30 min.

The synthesis of the initial hit (**3a**) and analogs with modifications to the aromatic moiety is outlined in Scheme 1. The aromatic ring was manipulated by introducing electron withdrawing and donating groups and hydrogen bond donors and acceptors. Carboxylic acid **1** was coupled under Steglich-like conditions (Pande et al., 2014) with alcohols **2a-m** furnishing the intended esters **3a-m**. Ester **3k'** was obtained through deprotection of **3k** under acidic conditions, and compounds **3l'** and **3m'** were generated from the reduction of **3l** and **3m**, respectively.

**Scheme 2:**
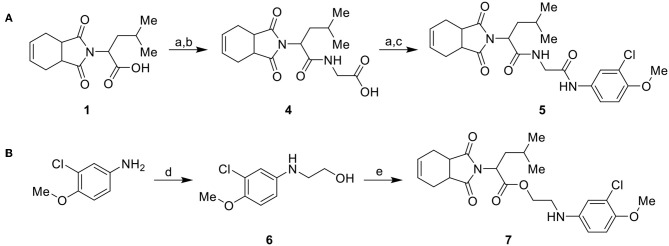
Synthesis of analogs of the initial hit **3a** with modifications to the ester and amide functional groups. **(A)** Synthesis of compound **5** by coupling intermediate **4** with 3-chloro-4-methoxyaniline. **(B)** Synthesis of compound **7** by coupling compoud **6** with **1**. Reagents and conditions: (a) CH_2_Cl_2_, oxalyl chloride, DMF (cat), rt, 2 h; (b) CH_2_Cl_2_, glycine, Et_3_N, rt, 2 h; (c) CH_2_Cl_2_, 3-chloro-4-methoxyaniline, Et_3_N, rt, 2 h; (d) DMF, ethylene carbonate, molecular sieve 4 Å (cat), 150°C, 4 d; (e) CH_2_Cl_2_, **1**, EDC, DMAP (cat), rt, 4 h.

Considering the low stability of the ester group in acidic and basic media, we synthesized a more stable analog with an amide group, as illustrated in Scheme 2. Initially, carboxylic acid intermediate **1** was transformed into an acyl chloride via reaction with oxalyl chloride. The acyl chloride was further used in a coupling reaction with glycine, producing intermediate **4**. Intermediate **4** was converted into an acyl chloride, using the same conditions previously mentioned, and coupled with 3-chloro-4-methoxyaniline to generate the target compound **5** (Scheme 2A). Compound **7** (Scheme 2B) was synthesized from 3-chloro-4-methoxyaniline with ethyl carbonate catalyzed by molecular sieves (Kinage et al., 2011) to produce **6**, followed by coupling with **1** using Steglich conditions.

**Scheme 3:**
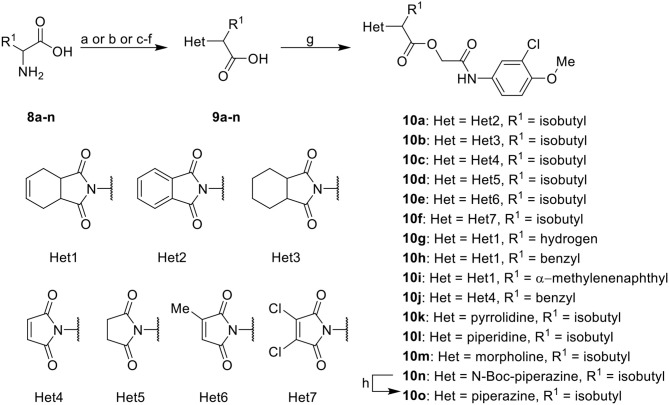
Synthesis of analogs of compound **3a** with changes to the imide and isobutyl moieties. Reagents and conditions: (a) (for compounds **9a-c** and **9g**) AcOH, cyclic anhydride (correspondent to Het), rt, o.n., then reflux, 4 h; (b) (for compounds **9d-f**, **9h-j**) toluene, cyclic anhydride (corresponding to Het), Et_3_N (cat), reflux, 3 h; (c) (for compounds **9k-n**) H_2_O, HBr (48% v/v in H_2_O), NaNO_2_, 0°C to rt, 3 h; (d) CH_2_Cl_2_, BnOH, EDC, DMAP (cat), rt, 4 h; (e) MeCN, amine (corresponding to Het), Cs_2_CO_3_, rt – 60°C, 30 min – o.n.; (f) MeOH, H_2_ 1 atm, 10% Pd/C, rt, 30 min; (g) CH_2_Cl_2_, **2a**, EDC, DMAP (cat), rt, 4 h; (h) CH_2_Cl_2_, HCl (4 M in 1,4-dioxane), rt, 4 h.

Further analogs of compound **3a** were designed to explore modifications to the imide nucleus and the hydrophobic isobutyl region (R^**1**^). The synthesis was performed according to Scheme 3 to evaluate the effects of changing the stereochemical and electronic features on the biological activity of the target compounds. Intermediates **9a-c** and **9g** were prepared according to a previously described procedure (Faghihi, 2008). A mixture of readily available *L*-amino acids and the corresponding cyclic anhydride in acetic acid stirred overnight, followed by 4 h of reflux to obtain carboxylic acids **9a-c** and **9g**. The synthesis of compounds **9d-f** and **9h-j** was carried out by refluxing in toluene with triethylamine as the catalyst. The stereocenter of the carboxylic acids was observed to have the absolute configuration of *R* (Pande et al., 2014). The amino acid side chains gave rise to hydrophobic fragments. Diazotization of *L*-leucine (Badiola et al., 2014) followed by bromoacid formation and subsequent nucleophilic substitution with the appropriate amines generated the amine nucleus in intermediates **9k-n**, presumably with an inverted configuration of the stereogenic center (*S*) due to inversion during the nucleophilic substitution reaction. Intermediates **9a-n** were coupled under Steglich conditions (Neises and Steglich, 1978) to produce the intended esters **10a-n**. Compound **10o** was produced by deprotection of the N-Boc analog (**10n**) under acidic conditions.

### Identification of Imide Derivatives as Cruzain Inhibitors

**Figure 2:**
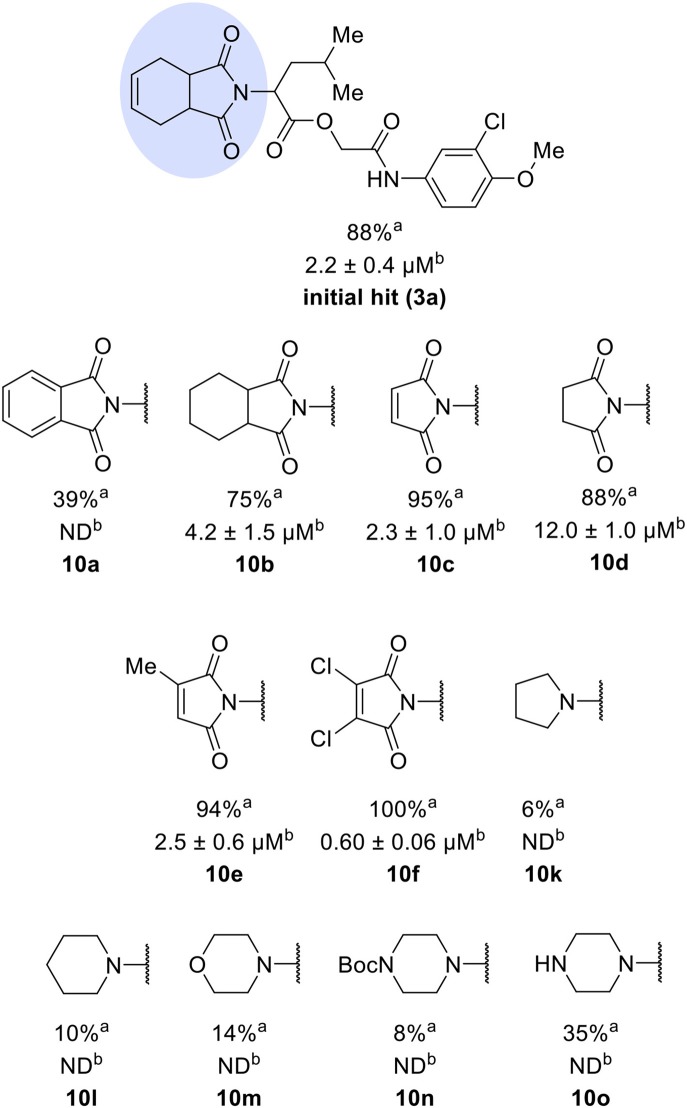
Activity of the series of analogs featuring modifications in the imide nucleus against cruzain. ^a^Percent inhibition against cruzain at 100 μM corresponding to the mean of three measurements. ^b^IC_50_ values against cruzain, which were independently determined by obtaining rate measurements in triplicate for at least six inhibitor concentrations. The IC_50_ values represent the mean ± SD of three individual experiments. ND, IC_50_ not determined.

The activities of inhibitor **3a** (IC_50_ = 2.2 μM) and its analogs having modifications to the imide moiety against cruzain are shown in Figure 2. The incorporation of an aromatic ring into the imide ring to form an isoindoline-1,3-dione core in compound **10a** reduced the percent inhibition from 88 to 39% at an inhibitor concentration of 100 μM. In fact, compound **10a** assumes a completely different binding conformation, with the imide projecting into the S2 subsite. This causes the loss of key interactions with the enzyme as predicted by the molecular docking simulations (Figure S1A). The coupling of a saturated ring to the imide resulted in a 2-fold decrease in the potency of compound **10b** (IC_50_ = 4.2 μM) over **3a**. The addition of unsaturation to the unsubstituted imide yielded compound **10c** (IC_50_ = 2.3 μM), which was equipotent with respect to inhibitor **3a**. In fact, compound **10c** preserves the enzyme-inhibitor interactions observed for **3a**, however, the imide carbonyl oxygen is predicted to interact with the side chain nitrogen of Trp184 instead of Gln19. The predicted binding modes of analogs **10b** and **10c** are shown in Figures S1B,C, respectively). Withdrawing the unsaturation of the imide produced compound **10d** (IC_50_ = 12.0 μM), which was 6-fold less active than **3a**.

Following the positive result observed for compound **10c**, we designed compounds **10e** and **10f**, which have the imide nucleus incorporating an unsaturation but with two different substitution patterns. Compound **10e**, with a methyl substituent, was equipotent (IC_50_ = 2.5 μM) relative to **10c**, whereas compound **10f**, featuring two chlorine atoms as substituents, resulted in a 6-fold increase in potency (IC_50_ = 0.60 μM). Replacing the imide core with an amine (**10k**, **10l**, **10m**, **10n**, and **10o**) led to a significant drop in the activity of the compounds, highlighting the essentiality of the carbonyl groups. In fact, Figure 1B shows a hydrogen bond between one of the carbonyl groups and the side chain nitrogen of Gln19 at the S1 subsite. Replacing the imide group with other ring systems, such as pyrrolidine, piperidine, and morpholine, abrogated the biological activity of this series. The predicted binding conformations of **10k** and **10l** (pyrrolidine and piperidine derivatives, respectively) show that the lack of the imide carbonyl groups changes the binding pattern of these compounds compared to that observed for the active analogs. As shown in Figure S2, the isobutyl and cyclic amines do not interact with the S1' and S1 subsites as expected, and become exposed to the solvent.

**Figure 3:**
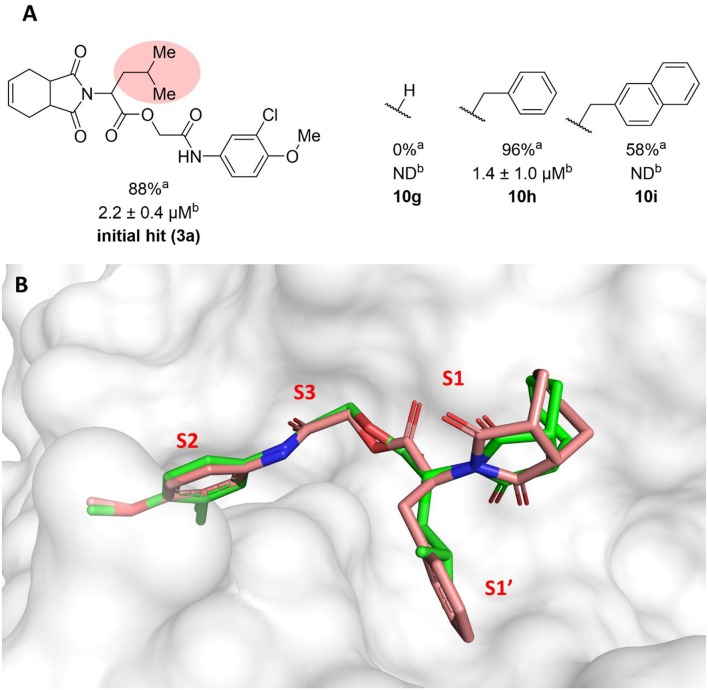
**(A)** Results for modifications of the isobutyl fragment. ^a^Percent inhibition against cruzain at 100 μM corresponding to the mean of three measurements. ^b^IC_50_ values against cruzain, which were independently determined by obtaining rate measurements in triplicate for at least six inhibitor concentrations. The IC_50_ values correspond to the mean ± SD of three individual experiments. ND, IC_50_ not determined. **(B)** Superposition of the molecular docking-predicted binding conformation of compounds **3a** (carbon in green) and compound **10 h** (carbon in salmon). Cruzain (PDB 3KKU, 1.28 Å) is depicted in surface representation. Inhibitors **3a** and **10 h** are shown as sticks. Cruzain subsites are labeled as S1, S1', S2, and S3.

The molecular optimization strategy also involved modifications to the hydrophobic (isobutyl) fragment of compound **3a** (Figure 3A). Removing the isobutyl group to obtain a methylene as the linker between the ester and the imide rendered the resulting compound inactive (**10g**). Bulky hydrophobic groups proved to be essential for interaction with the S1' subsite. Replacing the isobutyl with a methylene led to a completely different binding pattern from that observed for the active compounds. The docking algorithm was unable to place the imide into S1 and the aromatic ring into S2, which rendered compound **10g** inactive (Figure S3A). Replacement of the isobutyl by the bulkier and planar benzyl group increased the potency by 2-fold (**10h**, IC_50_ = 1.4 μM) compared to compound **3a**. This activity improvement can be reasoned to be a result of the better complementarity of the benzyl group with the S1' subsite of the enzyme (Figure 3B). Replacing the benzyl with the bulkier methylene naphthyl moiety led to compound **10i**, which was significantly less active, suggesting the ideal volume of a benzyl ring to occupy the S1' subsite. In fact, the naphthyl group of analog **10i** projects into the S2 subsite, which can be the driving force for the percent inhibition value (58%) determined for this compound since S2 usually accommodates bulky hydrophobic groups (Figure S3B). However, this modification caused the imide to lose its interaction with S1, which is a key driving force for the activity of this series.

**Figure 4:**
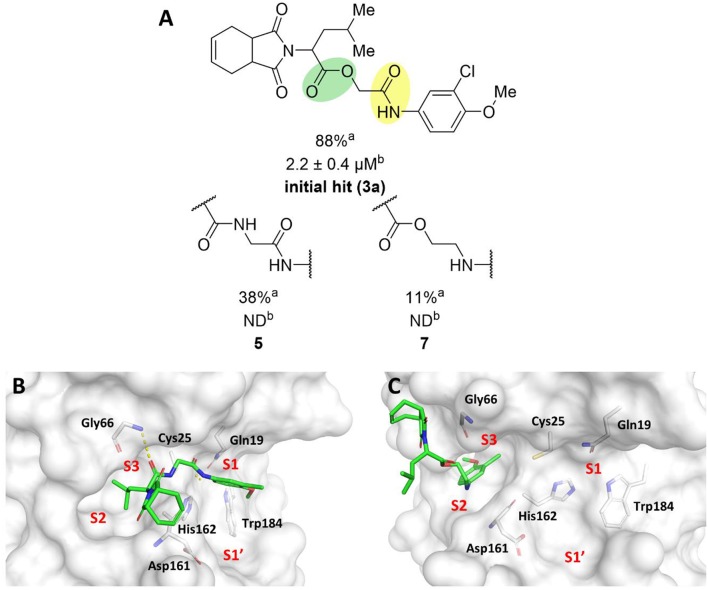
**(A)** Results for the modifications to the ester and secondary amide functions. ^a^Percent inhibition against cruzain at 100 μM corresponding to the mean of three measurements. ^b^IC_50_ values against cruzain, which were independently determined by obtaining rate measurements in triplicate for at least six inhibitor concentrations. The IC_50_ values correspond to the mean ± SD of three individual experiments. ND: IC_50_ not determined. **(B)** Molecular docking predicted binding mode of compound **5**. **(C)** Predicted binding mode of compound **7**. Cruzain (PDB 3KKU, 1.28 Å) is depicted in surface representation. Binding site residues (carbon in gray) and inhibitors (carbon in green) are shown as sticks. Hydrogen bonds are shown as dashed lines. Cruzain subsites are labeled as S1, S1', S2, and S3.

To probe the relevance of the ester and amide groups for the activity of cruzain inhibitors, compounds **5** and **7** were prepared (Figure 4A). Replacing the ester with an amide, resulting in compound **5**, caused a reduction in the percent inhibition from 88 to 38%. Converting the amide carbonyl into a methylene decreased the biological activity even further, as observed for compound **7** (11% inhibition). As illustrated in Figure 1B, the amide oxygen of compound **3a** is predicted to form a hydrogen bond with the main chain nitrogen of Gly66 and the ester carbonyl interacts with Gln19. The loss of these interactions may be one of the reasons for the significant activity drop observed for compound **7**. In fact, modifying the ester and amide groups significantly changed the binding mode of both compounds **5** and **7** (Figures 4B,C, respectively) compared to that of the active analogs. Compound **5**, which had the two carbonyl groups preserved, forms a hydrogen bond with Gly66 at S3, which can be reasoned as the cause of its higher percent inhibition value (38%) compared to that of analog **7** (11%). Notwithstanding, the imide of both compounds is exposed to the solvent and no interaction with S1' is observed, which are critical detrimental features of analogs **5** and **7**.

**Figure 5:**
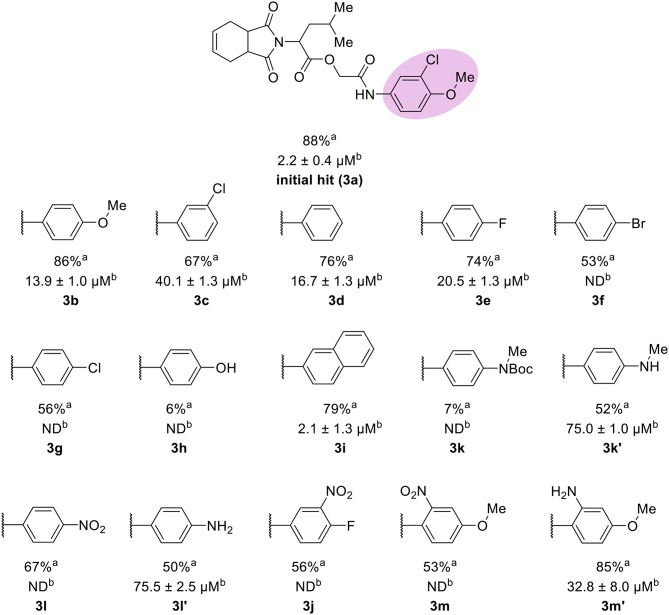
Results for the modifications to the aromatic ring. ^a^Percent inhibition against cruzain at 100 μM corresponding to the mean of three measurements. ^b^IC_50_ values against cruzain, which were independently determined by obtaining rate measurements in triplicate for at least six inhibitor concentrations. The IC_50_ values correspond to the mean ± SD of three individual experiments. ND, IC_50_ not determined.

The leverage of the 3-chloro-4-methoxyphenyl moiety on cruzain activity was assessed by modifying the substitution pattern of the aromatic ring (Figure 5). Removing either chlorine or methoxyphenyl led to the less active compounds **3b** (IC_50_ = 13.9 μM) and **3c** (IC_50_ = 40.1 μM), respectively. Compound **3b** retains the key polar interactions with the enzyme, however, the lack of the chlorine prevents an optimal interaction with the S2 pocket, which is known to be essential for potent cruzain inhibition as reported previously (Ferreira et al., 2014) (Figure S4A). The effect of removing the methoxy group from the S2-interacting aromatic ring proved to be more detrimental to activity than the removal of the chlorine. In fact, compound **3c** was predicted to lose most of the key polar interactions with the enzyme (Figure S4B), which resulted in a poorly active compound (IC_50_ = 40.1 μM). The unsubstituted phenyl derivative (**3d**) was 8-fold less active (IC_50_ = 16.7 μM) than the parent 3-chloro-4-methoxyphenyl compound (**3a**). Additional manipulations, such as introducing hydrogen bond donors and acceptors and replacing the chlorine or changing its position, led to poorly active or inactive compounds (**3e-h**, **3k**, **3k'**, **3l**, **3l'**, **3j**, **3m**, and **3m'**). Figures S4 and **S5** show that a suboptimal interaction between the aromatic ring and the S2 subsite lead to the loss of most, if not all, polar contacts that proved to be important for activity. The exception was compound **3i**, with naphthyl replacing the phenyl as the S2-interacting aromatic system (Figure S5D). This is consistent with the binding mode of these compounds, which shows that the aromatic moiety interacts with the S2 subsite (Figure 1B). The S2 subsite, which is mostly composed of hydrophobic amino acids, can accommodate bulky groups, as shown in our previous work on benzimidazole derivatives (Ferreira et al., 2014). Such groups can therefore promote an optimum interaction with the S2 subsite, leading to more potent cruzain inhibitors.

Since all compounds described so far were obtained from enantiopure amino acids, we next evaluated how the absolute configuration influences the activity against cruzain. To this end, the racemic form of compound **10h** was prepared (**10h**_rac_). The synthesis of **10h**_rac_ was achieved by the same procedure presented in Scheme 3, using *DL*-phenylalanine instead of *L*-phenylalanine. Chiral column HPLC and optical rotation analyses confirmed that all reactions did not lead to racemization when *L*-phenylalanine was used as the starting material. These analyses also confirmed that the racemic compound was obtained when *DL*-phenylalanine was used as the starting material. The racemate (**10h**_rac_) exhibited an IC_50_ value of 1.16 μM against cruzain, while compound **10h** had an IC_50_ of 1.4 μM. The very close IC_50_ values obtained for both the racemate and the enantiopure compound demonstrated that the absolute configuration of the stereocenter is not relevant to the activity of this series of compounds.

**Figure 6:**
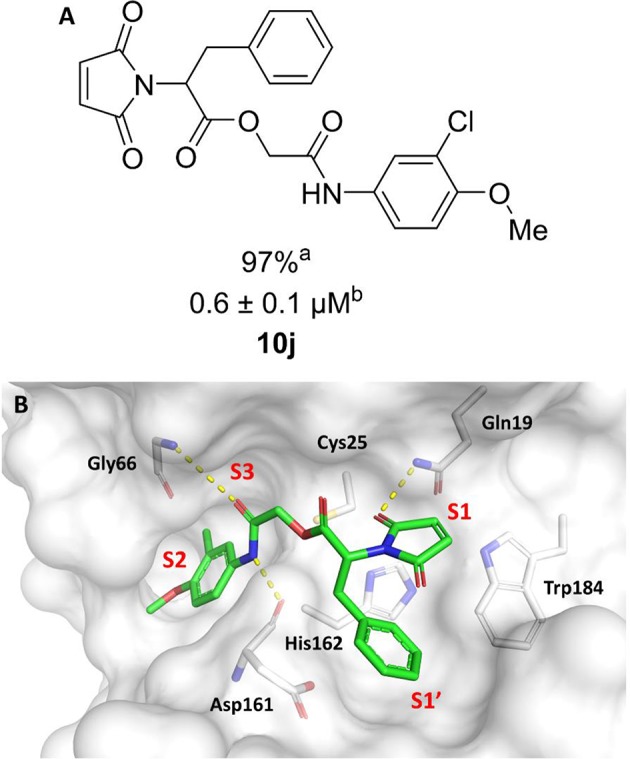
**(A)** Modifications to the imide nucleus and hydrophobic fragment. ^a^Percent inhibition against cruzain at 100 μM corresponding to the mean of three measurements. ^b^IC_50_ values against cruzain, which were independently determined by obtaining rate measurements in triplicate for at least six inhibitor concentrations. The IC_50_ values correspond to the mean ± SD of three individual experiments. ND, IC_50_ not determined. **(B)** Molecular docking predicted binding mode of compound **10j**. Cruzain (PDB 3KKU, 1.28 Å) is depicted in surface representation. Binding site residues (carbon in gray) and inhibitors (carbon in green) are shown as sticks. Hydrogen bonds are shown as dashed lines. Cruzain subsites are labeled as S1, S1', S2, and S3.

In the next molecular optimization step, we combined the best fragments from all five regions of initial hit **3a**. This strategy produced the most active compound (**10j**, IC_50_ = 0.6 μM) among the synthesized analogs, which was 4-fold more active than compound **3a** (Figure 6A). Compound **10j** conserves the same binding mode of the other active compounds in this series, forming the key intermolecular interactions with the S1, S1', S2, and S3 subsites. The S1' and S2 subsites are optimally filled with the benzyl and 3-chloro-4-methoxyphenyl rings, respectively. A hydrogen bond is formed between the amide oxygen and Gly66, and the imide carbonyl interacts with Gln19 (Figure 6B).

**Figure 7:**
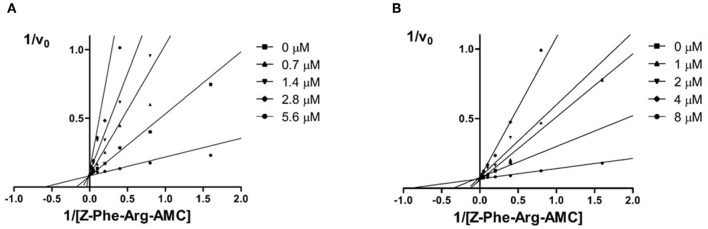
Double reciprocal *Lineweaver-Burk* plots for compounds **10 h** **(A)** and **3i (B)**. Each curve denotes to a different inhibitor concentration.

The aim of this study was to identify new imide derivatives as competitive cruzain inhibitors. Considering the competitive nature of hit compound **3a**, the synthesized analogs were expected to follow the same behavior. Hence, we conducted further studies to establish the mechanism of inhibition of compounds **10h** and **3i**. The competitive mechanism of these compounds was corroborated by the Lineweaver–Burk plots shown in Figure 7. As expected for competitive inhibitors, the maximum velocity (1/*V*_max_, intersections with the *y*-axis) remained unchanged with increasing inhibitor concentrations [I], while the apparent Michaelis–Menten constant ($K_{\text{M}}^{\text{app}}$ = – 1/*K*_M_, intersections with the *x*-axis) increased with escalating [I].

### Discovery of Novel Trypanocidal Agents

The biological activity of 22 compounds was evaluated against *T. cruzi* intracellular amastigotes along with **BZ** (Tables 1–**3**). Several compounds showed trypanocidal activity similar to or superior to that of **BZ** (IC_50_ = 3.0 μM). The data in Table 1 show that the hit compound **3a**, despite being a potent cruzain inhibitor, was not active against the parasite. The same behavior was observed for compound **10b**, which differs from **3a** only by the absence of a double bond in the ring coupled to the imide. Compounds having the imide with unsaturation and without the coupled six-membered ring showed promising trypanocidal activity (**10c**, **10e**, and **10f**, IC_50_ values of 0.9, 1.2 and 2 μM, respectively).

**Table 1:** Cruzain inhibition and trypanocidal activity of compounds with modifications to the imide nucleus.

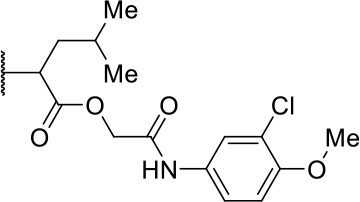			
**Compound**	**Structure**	**IC**_**50**_ **cruzain (μM)**^**a**^	**IC**_**50**_ ***T. cruzi*** **(μM)**^**b**^
Initial hit (3a)	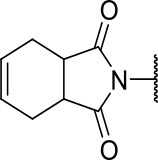	2.2 ± 0.4	>100
10b	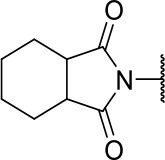	4.2 ± 1.5	>100
10c	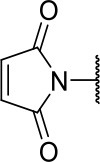	2.3 ± 1.0	0.9 ± 0.3
10d	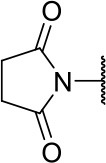	12.0 ± 1.0	18.5 ± 3.1
10e	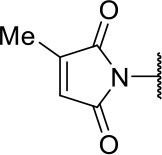	2.5 ± 0.6	1.2 ± 0.3
10f	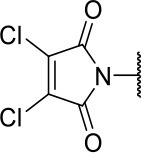	0.6 ± 0.1	2.0 ± 0.7
10k	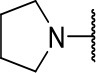	ND	>100
10l	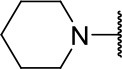	ND	11.6 ± 2.4
10m	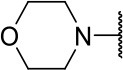	ND	1.8 ± 0.4
10n	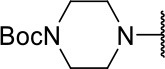	ND	1.6 ± 0.5
10o	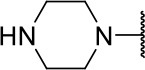	ND	1.7 ± 0.5
BZ	-	ND	3.0 ± 0.6

a *IC_50_ values against cruzain were independently determined by obtaining rate measurements in triplicate for at least six inhibitor concentrations. The values represent the mean ± SD of three individual experiments*.

b *IC_50_ values against T. cruzi represent the mean ± SD of two individual experiments. ND, not determined*.

Compounds lacking the imide carbonyl groups (**10l**-**10o**), although inactive against cruzain, were active against *T. cruzi*. Compound **10k**, in which the imide was replaced with a pyrrolidine, was inactive against both cruzain and *T. cruzi*. Replacing pyrrolidine with piperidine, a larger heterocycle, resulted in compound **10l**, which was moderately active against the parasite (IC_50_ = 11.6 μM). Introducing an additional heteroatom in the 6-membered cycle, whether nitrogen or oxygen, generated remarkably active compounds (**10m**, **10n**, and **10o**, IC_50_ values of 1.8, 1.6, and 1.7, respectively). These results indicate that these cyclic amines exert their trypanocidal activity by modulating a molecular target other than cruzain.

Table 2 shows the data for compounds modified on the hydrophobic fragment and the amide group. Replacing the isobutyl with a benzyl group resulted in compound **10h**, which, although active against cruzain, was inactive against the parasite. Notwithstanding, the benzyl group in compound **10j** combined with the maleimide fragment generated the most active compound against both *T. cruzi* (IC_50_ = 1.0 μM) and cruzain (IC_50_ = 0.6 μM). Compound **7**, in which the amide carbonyl was removed, displayed the opposite behavior to that of compound **10h**, i.e., **7** was active against *T. cruzi* but inactive on cruzain.

**Table 2:** Cruzain inhibition and trypanocidal activity of compounds with modifications to the hydrophobic fragment and the amide function.

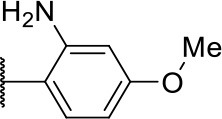			
**Compound**	**Structure**	**IC**_**50**_ **cruzain (μM)**^**a**^	**IC**_**50**_ ***T. cruzi*** **(μM)**^**b**^
10h	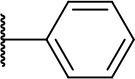	1.4 ± 0.8	>100
10j	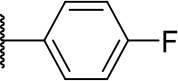	0.6 ± 0.1	1.0 ± 0.3
7	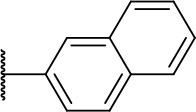	ND	1.5 ± 0.4

a *IC_50_ values against cruzain were independently determined by obtaining rate measurements in triplicate for at least six inhibitor concentrations. The values represent the mean ± SD of three individual experiments*.

b *IC_50_ values against T. cruzi represent the mean ± SD of two individual experiments. ND, not determined*.

As shown in Table 3, except for compounds **3k** and **3i**, modifications to the aromatic ring were unfavorable for activity against both *T. cruzi* and cruzain.

**Table 3:** Cruzain inhibition and trypanocidal activity of compounds with modifications to the aromatic ring.

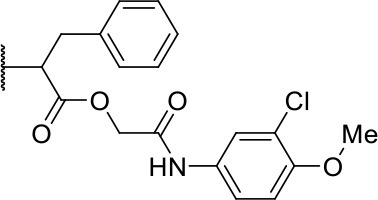			
**Compound**	**Structure**	**IC**_**50**_ **cruzain (μM)**^**a**^	**IC**_**50**_ ***T. cruzi*** **(μM)**^**b**^
3k	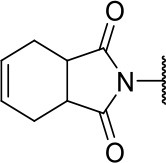	ND	3.9 ± 0.9
3k'	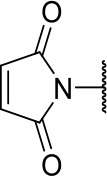	75.0 ± 1.0	>100
3l	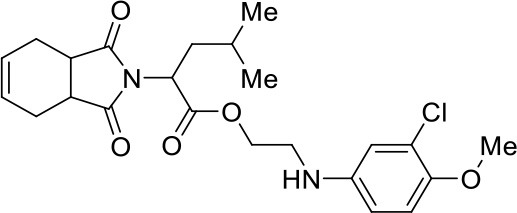	ND	>100
3l'	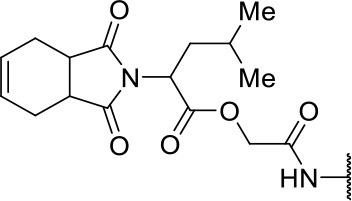	75.5 ± 2.5	>100
3m'	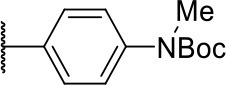	32.8 ± 8.0	>100
3d	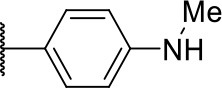	16.7 ± 1.3	>100
3e	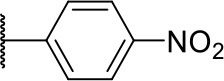	20.5 ± 1.3	>100
3i	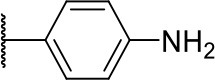	2.1 ± 1.3	32.8 ± 4.9

a *IC_50_ values against cruzain were independently determined by obtaining rate measurements in triplicate for at least six inhibitor concentrations. The values represent the mean ± SD of three individual experiments*.

b *IC_50_ values against T. cruzi represent the mean ± SD of two individual experiments. ND, not determined*.

**Figure 8:**
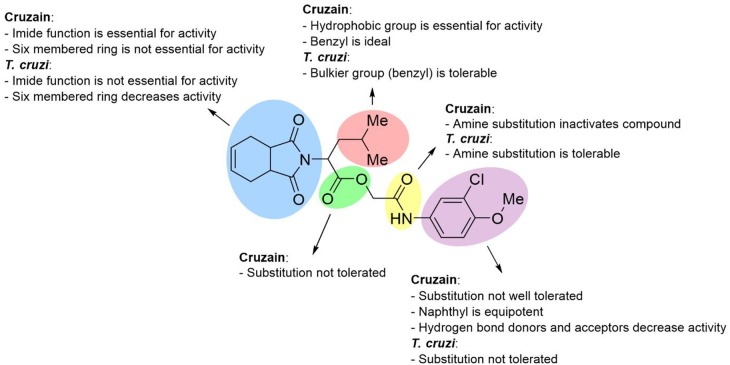
SAR scheme for both cruzain and *T. cruzi*. Compound **3a** was used as the reference structure.

Figure 8 illustrates an SAR scheme for the synthesized imide derivatives. Following our initial approach, compound **3a** was divided into five fragments, and the most relevant SARs were identified. In short, the imide function, although required for cruzain inhibition, is not essential for activity against *T. cruzi*. Benzyl is the ideal hydrophobic fragment for activity against cruzain and is tolerable regarding the activity against *T. cruzi*. Replacement of the 3-chloro-4-methoxyphenyl fragment is, in general, unfavorable for activity against *T. cruzi* and cruzain. Removal of the ester group is unfavorable for activity against cruzain, while the replacement of the secondary amide proved to be tolerable regarding the trypanocidal activity. The gathered data showed a lack of a direct correlation between the phenotypic and target-based results for some compounds; this was expected to some degree given the complexity of the intracellular environment and issues related to transport across membranes. For other compounds, however, the target-based and phenotypic data are clearly correlated. Compounds **10c**, **10e**, **10f**, and **10g**, for instance, besides being active against the enzyme, proved to be potent trypanocidal agents.

### *In vitro* Toxicity and Selectivity

The cytotoxicity of the synthesized analogs was assessed using HFF-1 human fibroblasts and HepG2 human hepatocytes (Table 4). The selectivity index (SI) was calculated as the ratio between the CC_50_ values for the human cells and the IC_50_ values for *T. cruzi*. In general, the designed compounds demonstrated no significant toxicity against HFF-1 and HepG2 cells. Three compounds showed SI values for HFF-1 fibroblasts comparable or superior to that of **BZ** (SI >33): **10m** (SI >55), **7** (SI >66) and **3k** (SI >25). It is worth noting that these compounds were active against *T. cruzi*. Another aspect worth mentioning is that the most cytotoxic compounds (**10c**, **10e**, and **10j**) are Michael acceptors, which is a possible reason for their cytotoxicity. With respect to cytotoxicity to HepG2 cells, most compounds produced higher SI values than that of **BZ**, particularly those that are highly active against *T. cruzi*: **10c** (SI = 36.6), **10f** (SI >32), **10m** (SI >35), **10o** (SI = 30.6), **10j** (SI = 67), and **7** (SI = 42).

**Table 4:** Comparison of the trypanocidal activity with cytotoxicity data obtained for HFF-1 and HepG2 cells.

**Compound**	**IC_**50**_*T. cruzi* (μM)^**a**^**	**CC_**50**_ HFF-1 (μM)^**b**^**	**SI HFF-1/*T. cruzi*^**c**^**	**CC_**50**_ HepG2 (μM)^**b**^**	**SI HepG2/*T. cruzi*^**c**^**
BZ	3.0 ± 0.6	>100	>33	>64	>21
10c	0.9 ± 0.3	12 ± 1	13.3	33 ± 5	36.6
10d	18.5 ± 3.1	>100	>5	>64	>3
10e	1.2 ± 0.3	4.8 ± 0.5	4	5 ± 1	4.2
10f	2.0 ± 0.7	33 ± 9	16.5	>64	>32
10l	11.6 ± 2.4	>100	>8	>64	>5
10m	1.8 ± 0.4	> 100	>55	>64	>35
10n	1.6 ± 0.5	32 ± 5	20	27 ± 2	16.9
10o	1.7 ± 0.5	32 ± 3	18.8	52 ± 7	30.6
10j	1.0 ± 0.3	15.7 ± 0.8	15.7	67 ± 8	67
7	1.5 ± 0.4	>100	>66	63 ± 8	42
3k	3.9 ± 0.9	>100	>25	>64	>16
3i	32.8 ± 4.9	>100	>3	45 ± 8	1.4
Doxorubicin	–	0.21 ± 0.09	–	0.4 ± 0.1	–

a *T. cruzi intracellular amastigote assay. Data represent the mean ± SD of two independent assays*.

b *Cytotoxicity assay. Data represent the mean ± SD of two independent assays*.

c *Selectivity index (SI), CC_50_/IC_50_*.

## Conclusion

The SBDD strategy applied herein, comprising synthetic organic chemistry, molecular docking, enzyme kinetics and phenotypic assays, resulted in the discovery of potent, reversible and non-peptidic cruzain inhibitors with remarkable trypanocidal activity. The success of this experimental-computational molecular optimization approach can be illustrated by compound **10j**, the most potent cruzain inhibitor (IC_50_ = 0.6 μM), which is significantly more active than initial hit **3a** (IC_50_ = 2.2 μM). One of the most potent cruzain inhibitors reported to date, compound **10j** represents a new chemical class among the known inhibitors of this enzyme. Furthermore, compound **10j** shows trypanocidal activity (IC_50_ = 1.0 μM) that is 3-fold higher than that of the clinically used drug **BZ** (IC_50_ = 3.0 μM). Other promising compounds are **10c**, **10f**, **10m**, **10n**, **10o**, **10j**, **7** and **3k**, which showed trypanocidal activity comparable to that of **BZ** and SI values (HFF-1/*T. cruzi*) >10.

The target-based results enabled the identification of relevant SARs, which allowed the uncovering of pivotal structural aspects that drive the enzyme-inhibitor molecular recognition and the activity of the investigated compounds. Moreover, these findings provide substantial insights into the design of reversible cruzain inhibitors, which can be useful to surmount the drawbacks associated with irreversible ligands. An important aspect to remark is the *in vitro* toxicity profile of some compounds, which indicates that they may be safer than the drugs currently available for the treatment of Chagas disease. In a context characterized by a lack of therapeutic innovation and serious safety and efficacy issues, the cruzain inhibitors described herein can be explored as novel chemical matter in forthcoming Chagas disease drug discovery campaigns.

## Data Availability Statement

All datasets generated for this study are included in the article/Supplementary Material.

## Author Contributions

RAF: conceptualization, writing, molecular design, and organic synthesis. IP: conceptualization, writing, molecular design, and enzyme kinetics. TS: organic synthesis. CR: organic synthesis. MS: molecular design and enzyme kinetics. RSF: conceptualization, molecular design, and enzyme kinetics. LM and RK: *in vitro* experiments. LF: writing, molecular design, and *in vitro* experiments. LD: conceptualization, supervision, organic synthesis, and writing. AA: conceptualization, supervision, molecular design, and writing.

### Conflict of Interest

The authors declare that the research was conducted in the absence of any commercial or financial relationships that could be construed as a potential conflict of interest.

## Footnotes

^1^https://www.who.int/chagas/en/

## Supplementary Material

The Supplementary Material for this article including NMR spectra (Figures S6–S70) can be found online at: https://www.frontiersin.org/articles/10.3389/fchem.2019.00798/full#supplementary-material
