# Supplementary material for: Structure-Based and Molecular Modeling Studies for the Discovery of Cyclic Imides as Reversible Cruzain Inhibitors With Potent Anti-Trypanosoma cruzi Activity
Source: Front Chem. 2019 Nov 25;7:798. doi: 10.3389/fchem.2019.00798 (PMC6886403; doi:10.3389/fchem.2019.00798)
Supplement: Supplementary file 1 [file Image_1.pdf]

Supporting Information

Structure-Based and Molecular Modeling Studies for the Discovery of Cyclic Imides as Reversible Cruzain Inhibitors with Potent Anti-*Trypanosoma cruzi* Activity

Rafael A. A. Ferreira,[a+] Ivani Pauli,[b+] Thiago S. Sampaio,[a] Mariana L. de Souza,[b] Leonardo L. G. Ferreira,[b] Luma G. Magalhães,[b] Celso de O. Resende Jr,[a] Rafaela S. Ferreira,[c] Renata Krogh,[b] Luiz C. Dias,[a*] Adriano D. Andricopulo[b*]

Affiliation

[a]Instituto de Química, Universidade Estadual de Campinas, 6154, Campinas, SP, Brazil

[b]Laboratório de Química Medicinal e Computacional, Centro de Pesquisa e Inovação em Biodiversidade e Fármacos, Instituto de Física de São Carlos, Universidade de São Paulo, São Carlos, SP, Brazil

[c]Departamento de Bioquímica e Imunologia, Universidade Federal de Minas Gerais, Belo Horizonte, MG, Brazil

Correspondence

Adriano D. Andricopulo: aandrico@ifsc.usp.br

Luiz Carlos Dias: ldias@unicamp.br

[+] These authors contributed equally to this work

Table of contents:

Supplementary Figures S1

NMR Spectra S5

Supplementary Figures

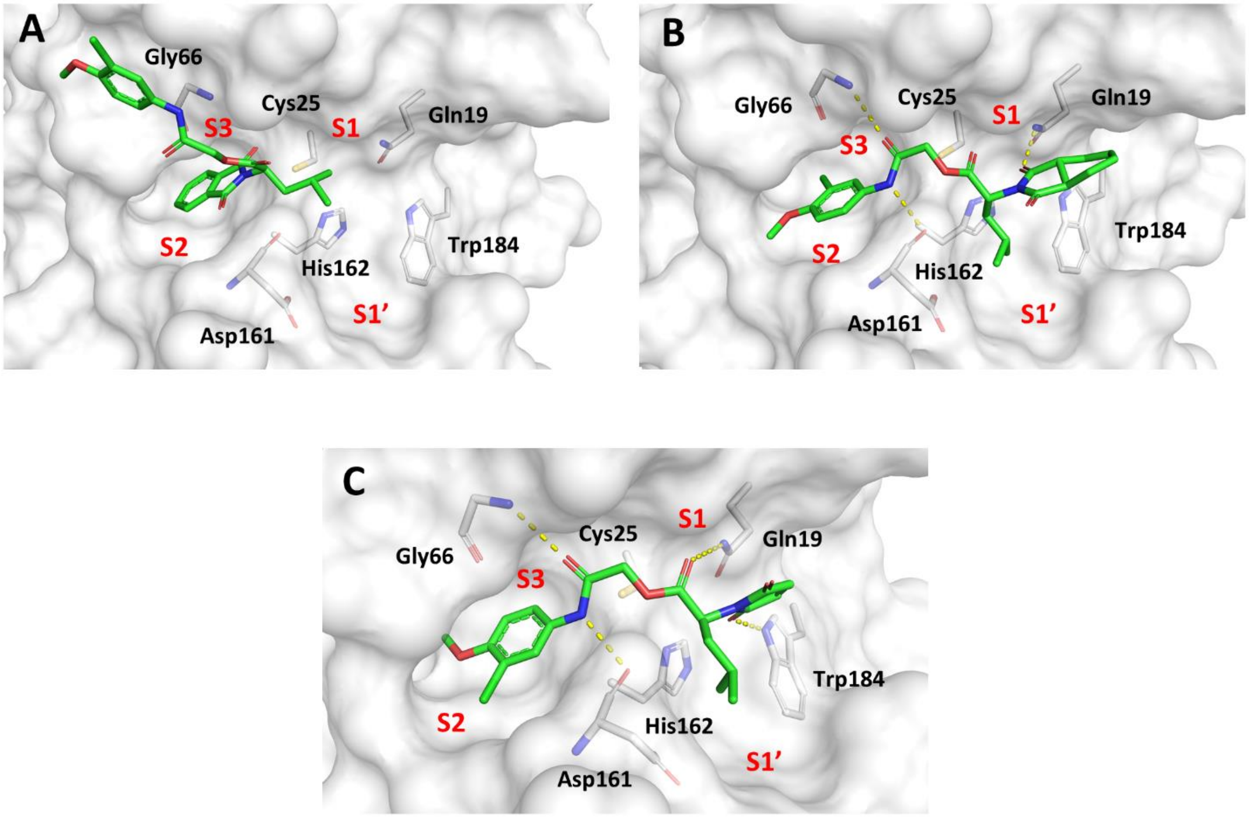

Figure S1. Molecular docking predicted binding mode of imide derivatives within the active site of cruzain. **(A)** Compound **10a**. **(B)** Compound **10b**. **(C)** Compound **10c**. Cruzain (PDB 3KKU, 1.28 Å) is depicted in surface representation. Binding site residues (carbon in gray) and inhibitors (carbon in green) are shown as sticks. Hydrogen bonds are shown as dashed lines. Cruzain subsites are labeled as S1, S1', S2 and S3.

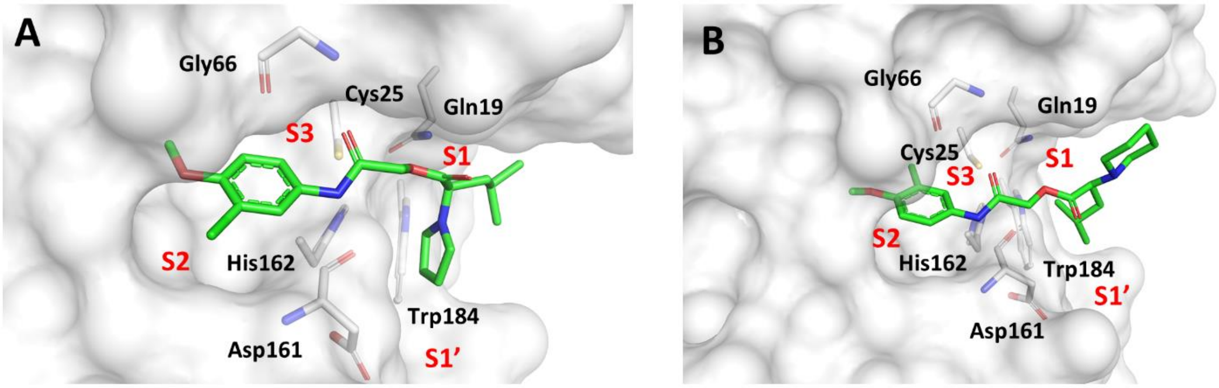

Figure S2. Molecular docking predicted binding mode of imide derivatives within the active site of cruzain. **(A)** Compound **10k**. **(B)** Compound **10l**. Cruzain (PDB 3KKU, 1.28 Å) is depicted in surface representation. Binding site residues (carbon in gray) and inhibitors (carbon in green) are shown as sticks. Hydrogen bonds are shown as dashed lines. Cruzain subsites are labeled as S1, S1’, S2 and S3.

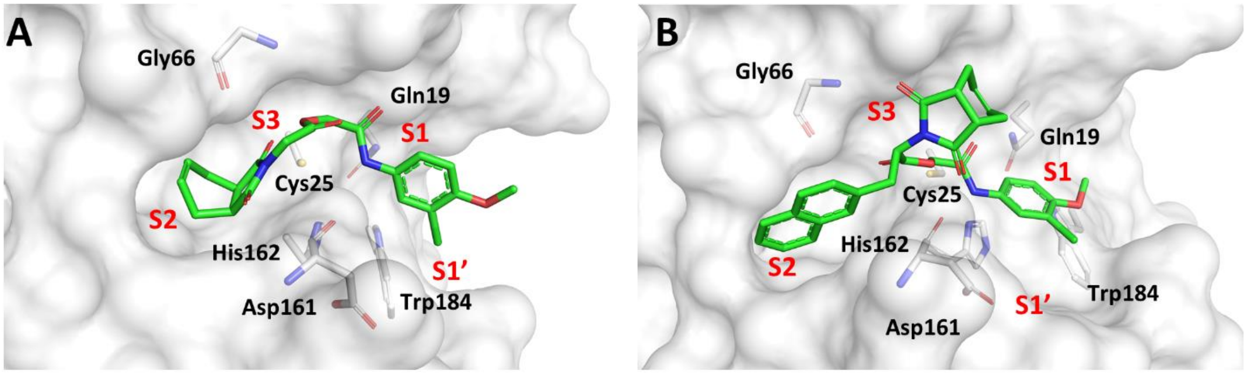

Figure S3. Molecular docking predicted binding mode of imide derivatives within the active site of cruzain. **(A)** Compound **10g**. **(B)** Compound **10i**. Cruzain (PDB 3KKU, 1.28 Å) is depicted in surface representation. Binding site residues (carbon in gray) and inhibitors (carbon in green) are shown as sticks. Hydrogen bonds are shown as dashed lines. Cruzain subsites are labeled as S1, S1’, S2 and S3.

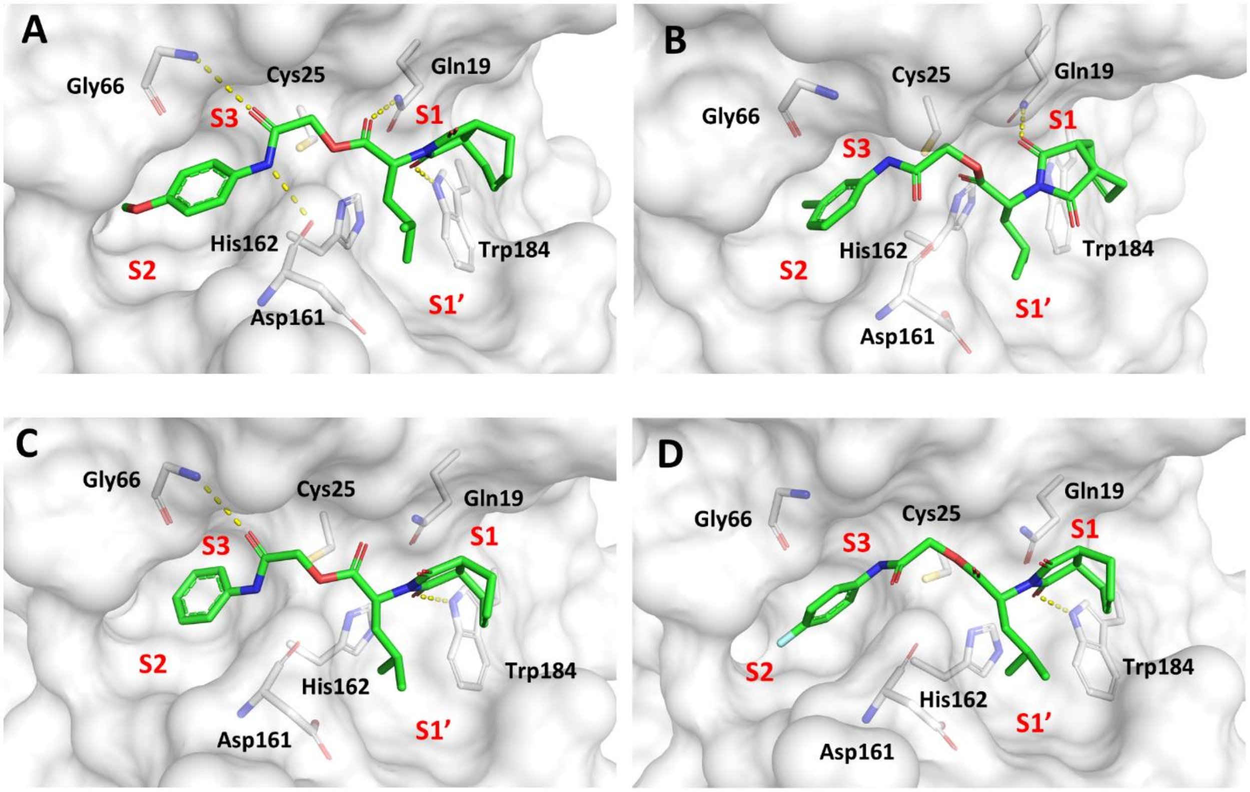

Figure S4. Molecular docking predicted binding mode of imide derivatives within the active site of cruzain. **(A)** Compound **3b**. **(B)** Compound **3c**. **(C)** Compound **3d (D)** Compound **3e**. Cruzain (PDB 3KKU, 1.28 Å) is depicted in surface representation. Binding site residues (carbon in gray) and inhibitors (carbon in green) are shown as sticks. Hydrogen bonds are shown as dashed lines. Cruzain subsites are labeled as S1, S1’, S2 and S3.

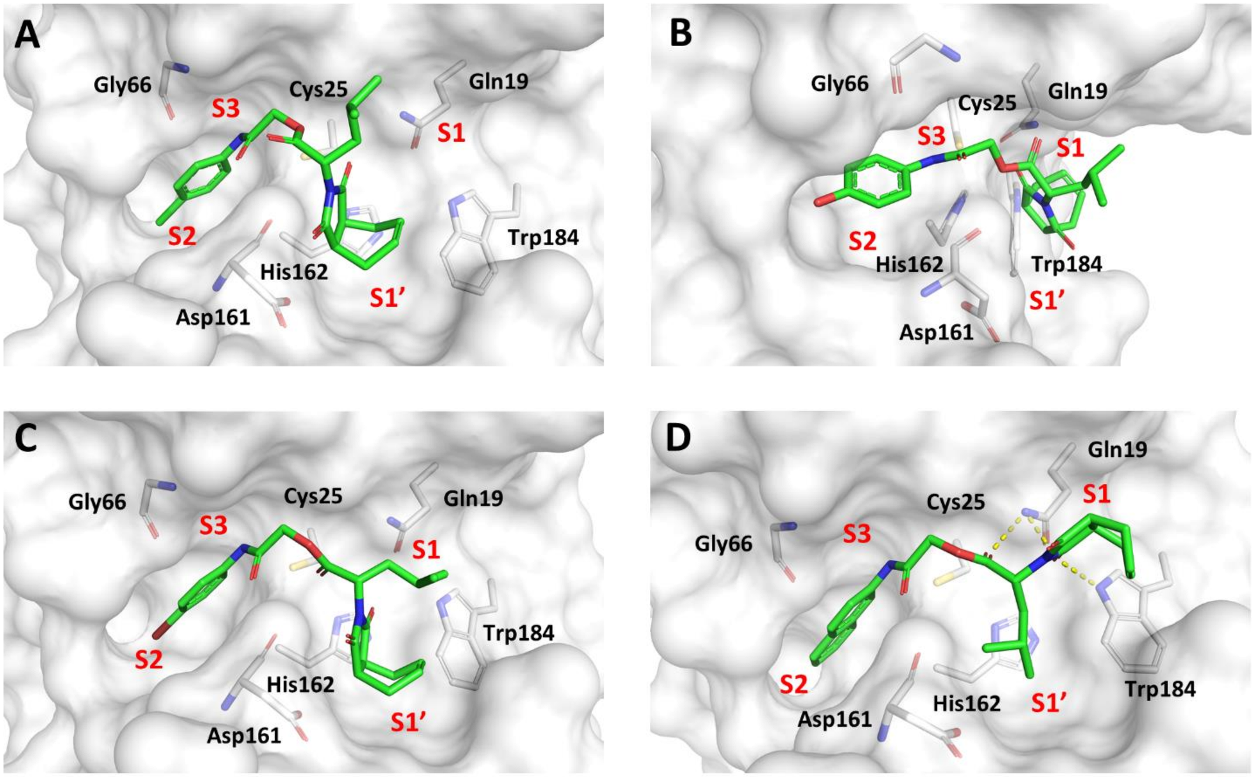

Figure S5. Molecular docking predicted binding mode of imide derivatives within the active site of cruzain. **(A)** Compound **3g**. **(B)** Compound **3h**. **(C)** Compound **3f**. **(D)** Compound **3i**. Cruzain (PDB 3KKU, 1.28 Å) is depicted in surface representation. Binding site residues (carbon in gray) and inhibitors (carbon in green) are shown as sticks. Hydrogen bonds are shown as dashed lines. Cruzain subsites are labeled as S1, S1', S2 and S3.

NMR Spectra

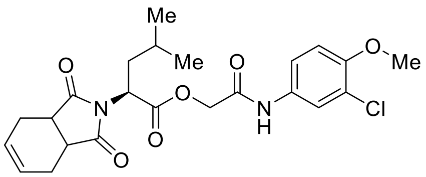

Acquisition Time (sec)	1.5903	*Comment*	Rafael - RAF 105 - CDCl3 - Avance 500 MHz - set02rafH1 - 1H			*Date*	02 Sep 2015 07:37:18
File Name	E:\RMN Dissert\500 MHz\Set 15\set02rafH1 RAF 105_001001r			*Frequency (MHz)*	499.87	*Nucleus*	1H
Number of Transients	16	*Original Points Count*	16384	*Points Count*	65536	*Pulse Sequence*	zg30
Solvent	CHLOROFORM-D	*Sweep Width (Hz)*	10302.20	*Temperature (degree C)*	25.150		

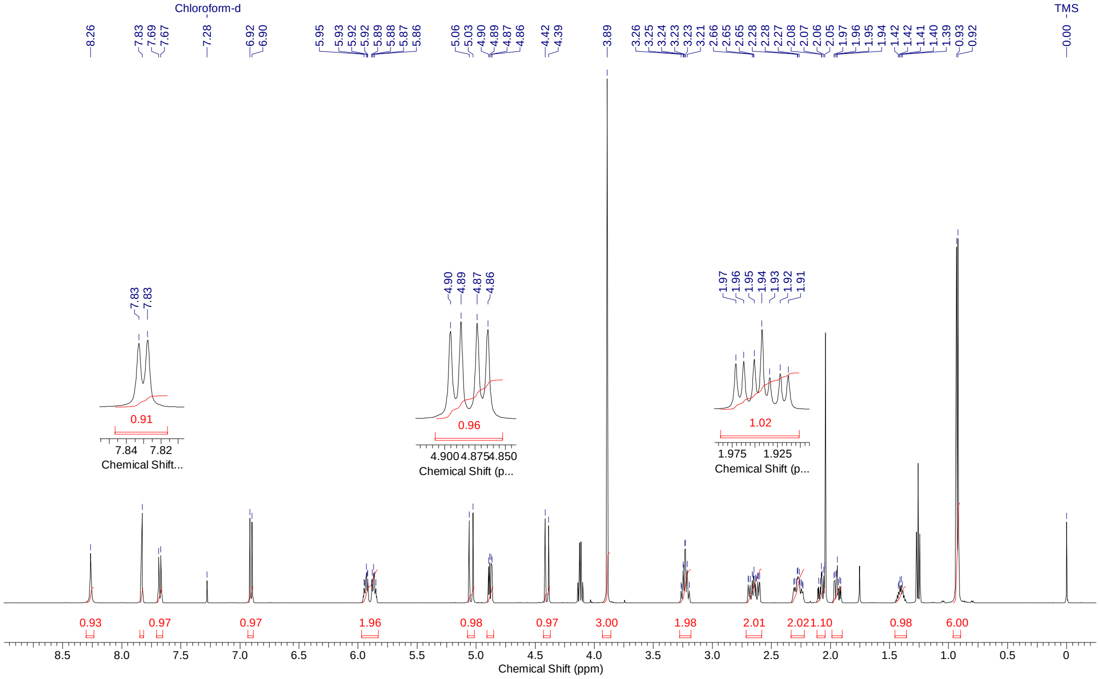

Figure S6 ^{1}H NMR spectrum of compound **3a** (500 MHz, $CDCl_3$).

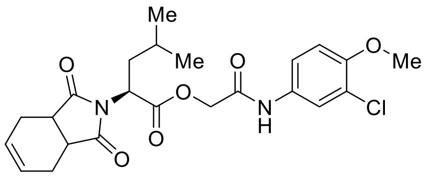

Acquisition Time (sec)	0.4981	***Comment***	Rafael - RAF 105 - CDCl3 - Avance 500 MHz - set02rafH1 - 13C			***Date***	02 Sep 2015 07:41:38
File Name	E:\RMN Dissert\500 MHz\Set 15\set02rafH1 RAF 105_002001r			***Frequency (MHz)***	125.69	***Nucleus***	13C
Number of Transients	128	***Original Points Count***	16384	***Points Count***	32768	***Pulse Sequence***	zgpg30
Solvent	CHLOROFORM-D	***Sweep Width (Hz)***	32894.74	***Temperature (degree C)***	25.211		

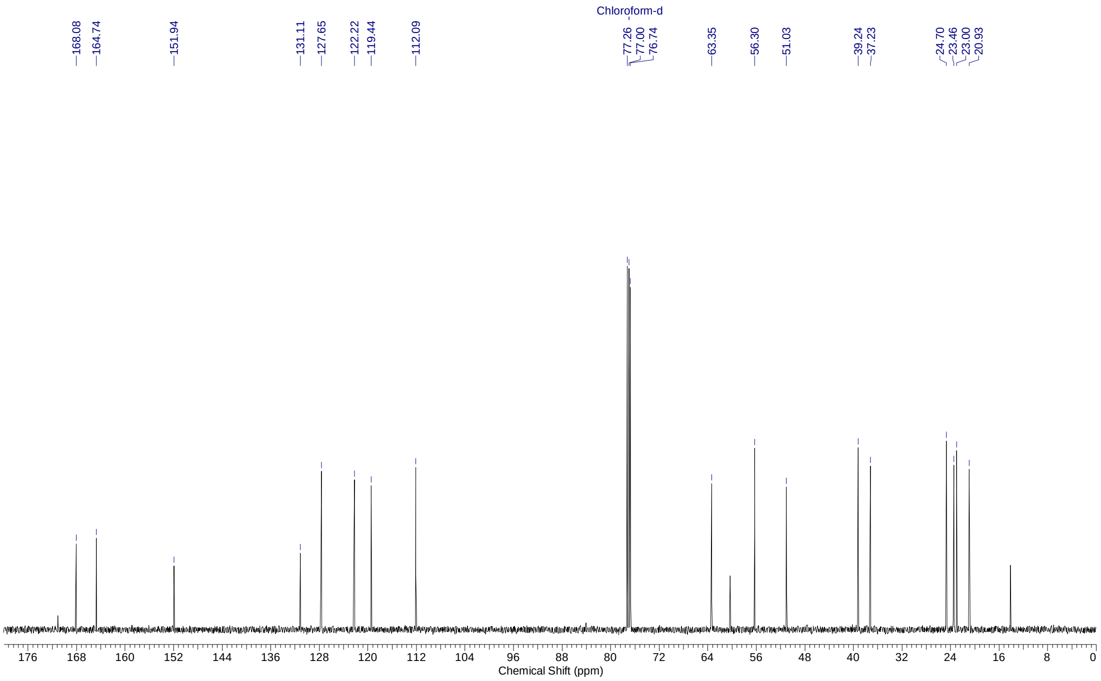

Figure S7 ^{13}C NMR spectrum of compound **3a** (125 MHz, $CDCl_3$).

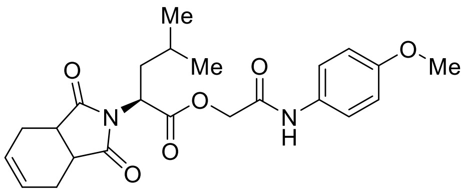

Acquisition Time (sec)	3.1654	***Comment***	Thiago TSS-027 DMSO-d6 250 MHz set20tssH4			***Date***	20 Sep 2012 19:53:52
Date Stamp	20 Sep 2012 19:53:52			***File Name***	C:\USERS\RAFAEL\DESKTOP\ESPECTROS TSS\250 - 2012\SET20TSSH4\1\PDATA\1\1R		
Frequency (MHz)	250.13	***Nucleus***	1H	***Number of Transients***	16	***Origin***	spect
Original Points Count	16384	***Owner***	root	***Points Count***	32768	***Pulse Sequence***	zg30
Receiver Gain	456.10	***SW(cyclical) (Hz)***	5175.98	***Solvent***	DMSO-d6	***Spectrum Offset (Hz)***	1755.9128
Spectrum Type	STANDARD	***Sweep Width (Hz)***	5175.83	***Temperature (degree C)***	25.160		

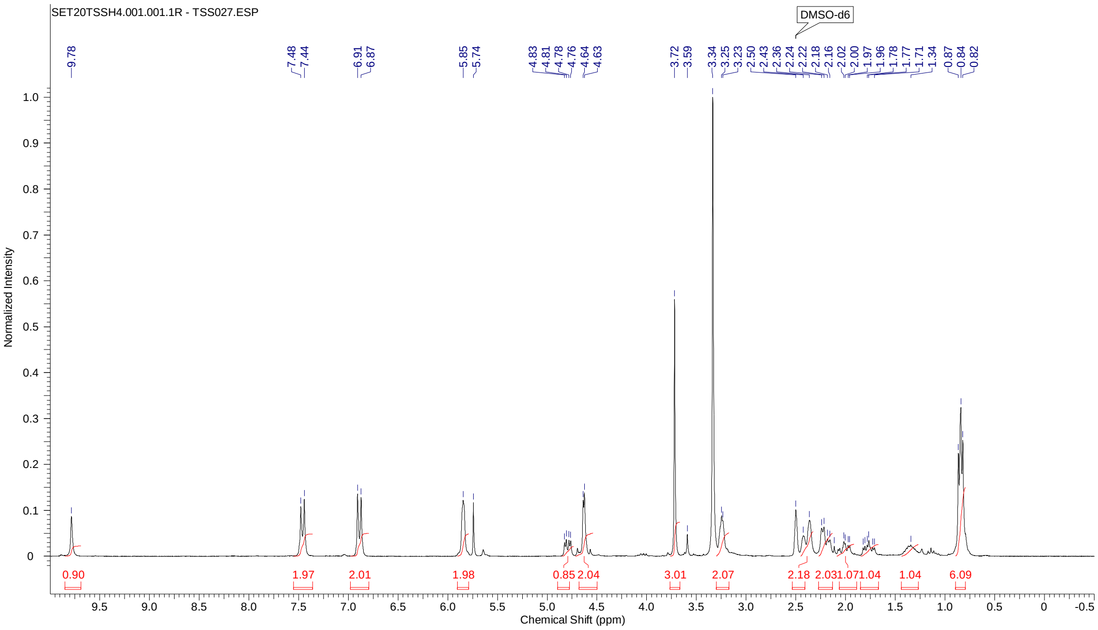

Figure S8 ^{1}H NMR spectrum of compound **3b** (250 MHz, DMSO).

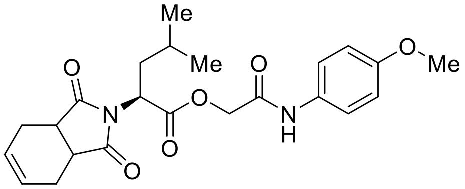

Acquisition Time (sec)	0.5439	***Comment***	Thiago TSS-027 DMSO-d6 250 MHz set20tssC4 13C			***Date***	20 Sep 2012 20:17:20
Date Stamp	20 Sep 2012 20:17:20			***File Name***	C:\USERS\RAFAEL\DESKTOP\ESPECTROS TSS\250 - 2012\SET20TSSC4\2\PDATA\1\1R		
Frequency (MHz)	62.90	***Nucleus***	13C	***Number of Transients***	1024	***Origin***	spect
Original Points Count	8192	***Owner***	root	***Points Count***	32768	***Pulse Sequence***	zgpg30
Receiver Gain	724.10	***SW(cyclical) (Hz)***	15060.24	***Solvent***	DMSO-d6	***Spectrum Offset (Hz)***	6261.3115
Spectrum Type	STANDARD	***Sweep Width (Hz)***	15059.78	***Temperature (degree C)***	25.160		

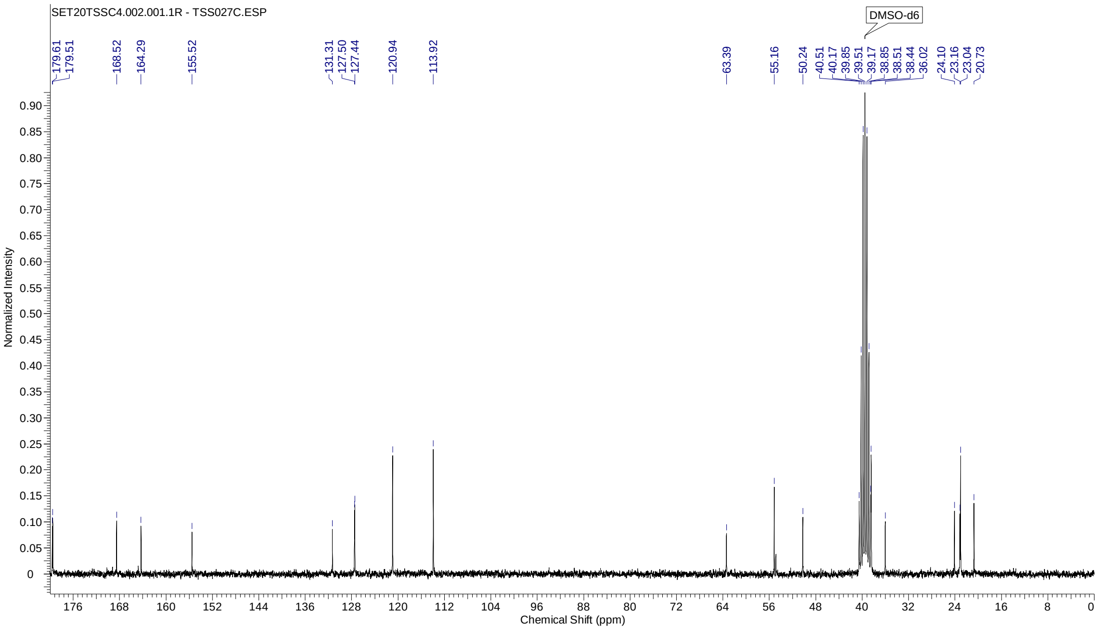

Figure S9 ^{13}C NMR spectrum of compound **3b** (62.5 MHz, DMSO).

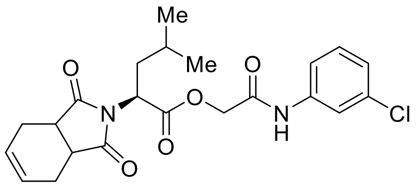

Acquisition Time (sec)	3.1654	***Comment***	Thiago_DMSO_250mHZ_TSS-028_set25tssH1			***Date***	25 Sep 2012 11:13:20
Date Stamp	25 Sep 2012 11:13:20			***File Name***	C:\USERS\RAFAEL\DESKTOP\ESPECTROS TSS\250 - 2012\SET25TSSH1\1\PDATA\1\1R		
Frequency (MHz)	250.13	***Nucleus***	1H	***Number of Transients***	16	***Origin***	spect
Original Points Count	16384	***Owner***	root	***Points Count***	32768	***Pulse Sequence***	zg30
Receiver Gain	114.00	***SW(cyclical) (Hz)***	5175.98	***Solvent***	DMSO-d6	***Spectrum Offset (Hz)***	1756.0708
Spectrum Type	STANDARD	***Sweep Width (Hz)***	5175.83	***Temperature (degree C)***	25.160		

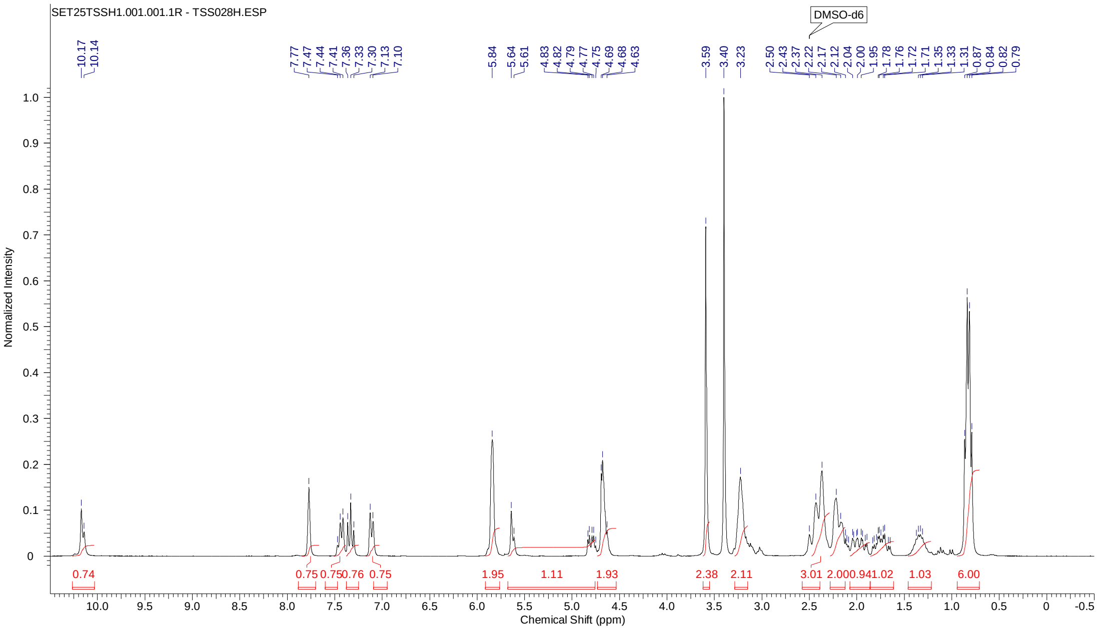

Figure S10 ^{1}H NMR spectrum of compound **3c** (250 MHz, DMSO).

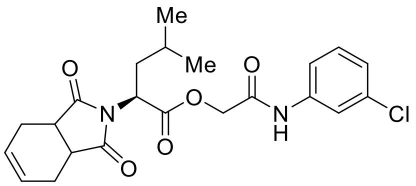

Acquisition Time (sec)	0.5439	***Comment***	Thiago_DMSO_250mHZ_TSS-028_set25tssC113C			***Date***	25 Sep 2012 12:00:16
Date Stamp	25 Sep 2012 12:00:16			***File Name***	C:\USERS\RAFAEL\DESKTOP\ESPECTROS TSS\250 - 2012\SET25TSSC1\2\PDATA\1\1R		
Frequency (MHz)	62.90	***Nucleus***	13C	***Number of Transients***	1024	***Origin***	spect
Original Points Count	8192	***Owner***	root	***Points Count***	32768	***Pulse Sequence***	zgpg30
Receiver Gain	1448.20	***SW(cyclical) (Hz)***	15060.24	***Solvent***	DMSO-d6	***Spectrum Offset (Hz)***	6264.9883
Spectrum Type	STANDARD	***Sweep Width (Hz)***	15059.78	***Temperature (degree C)***	25.160		

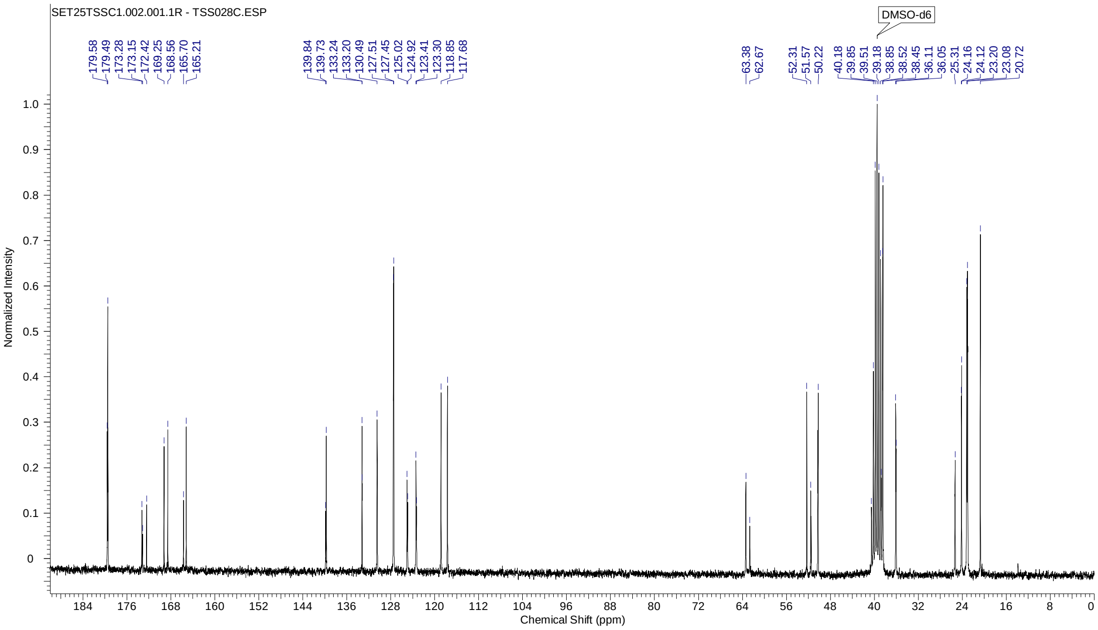

Figure S11 ^{13}C NMR spectrum of compound **3c** (62.5 MHz, DMSO).

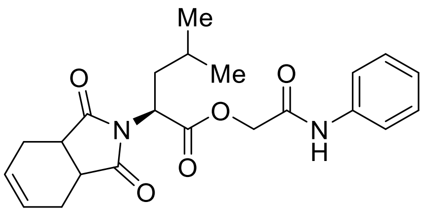

Acquisition Time (sec)	1.9923	***Comment***	Thiago - TSS-085 - dmso - Avance 400MHz - fev04tssH1			***Date***	04 Feb 2013 12:34:40
Date Stamp	04 Feb 2013 12:34:40			***File Name***	C:\USERS\RAFAEL\DESKTOP\ESPECTROS TSS\400 - 2013\FEV04TSSH1\1\PDATA\1\1R		
Frequency (MHz)	400.18	***Nucleus***	1H	***Number of Transients***	16	***Origin***	spect
Original Points Count	16384	***Owner***	avance	***Points Count***	32768	***Pulse Sequence***	zg30
Receiver Gain	64.00	***SW(cyclical) (Hz)***	8223.68	***Solvent***	DMSO-d6	***Spectrum Offset (Hz)***	2463.7126
Spectrum Type	STANDARD	***Sweep Width (Hz)***	8223.43	***Temperature (degree C)***	25.160		

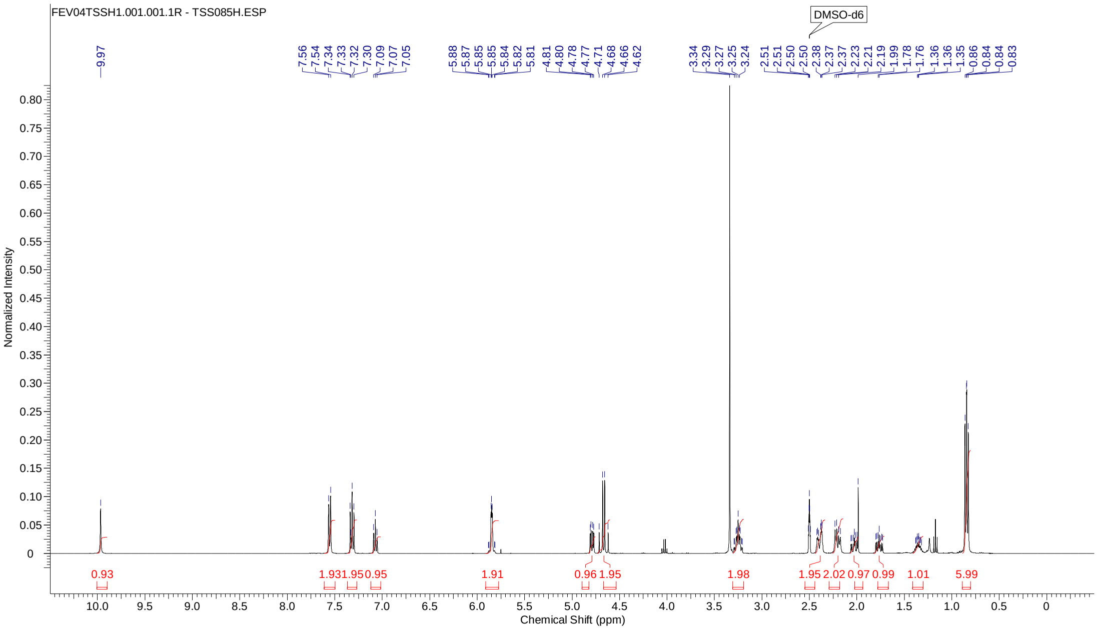

Figure S12 ^{1}H NMR spectrum of compound **3d** (400 MHz, DMSO).

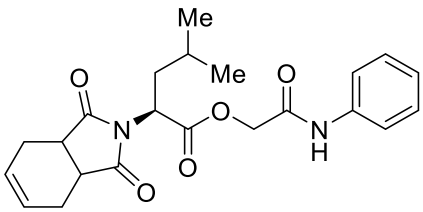

Acquisition Time (sec)	0.6816	***Comment***	Thiago - TSS-085 - dmso - Avance 400MHz - fev04tssH1 - 13C			***Date***	04 Feb 2013 12:58:08
Date Stamp	04 Feb 2013 12:58:08			***File Name***	C:\USERS\RAFAEL\DESKTOP\ESPECTROS TSS\400 - 2013\FEV04TSSH1\2\PDATA\1\1R		
Frequency (MHz)	100.63	***Nucleus***	13C	***Number of Transients***	5120	***Origin***	spect
Original Points Count	16384	***Owner***	avance	***Points Count***	131072	***Pulse Sequence***	zgpg30
Receiver Gain	203.00	***SW(cyclical) (Hz)***	24038.46	***Solvent***	DMSO-d6	***Spectrum Offset (Hz)***	10012.3740
Spectrum Type	STANDARD	***Sweep Width (Hz)***	24038.28	***Temperature (degree C)***	25.160		

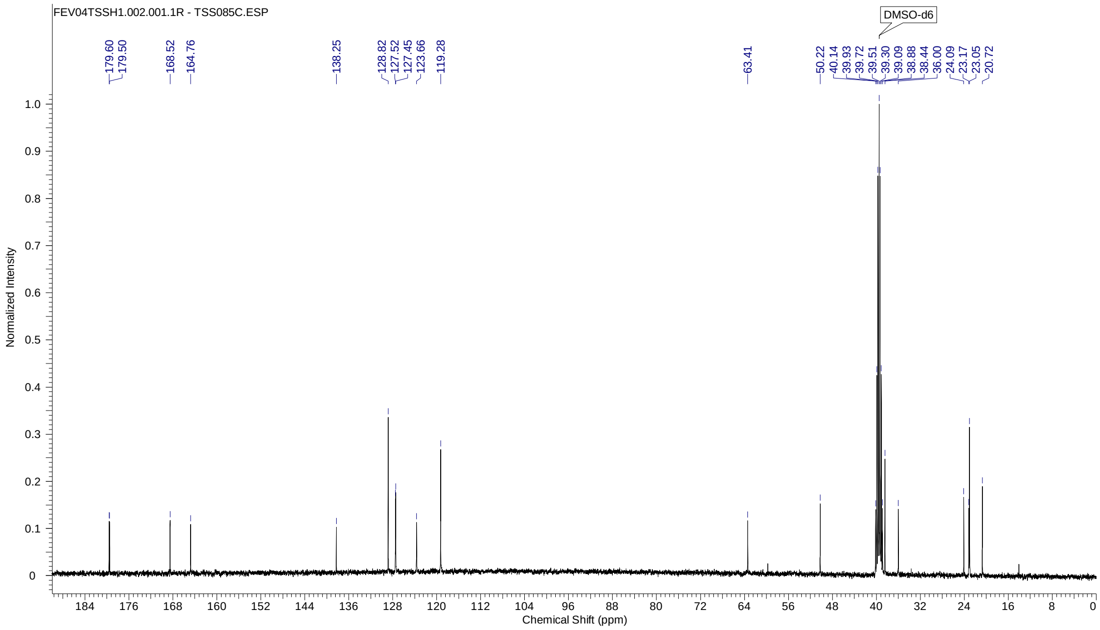

Figure S13 ^{13}C NMR spectrum of compound **3d** (100 MHz, DMSO).

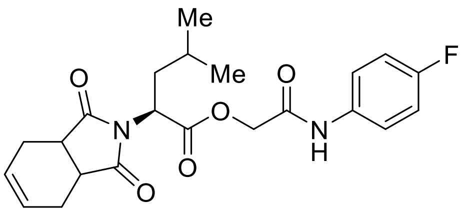

Acquisition Time (sec)	3.1654	***Comment***	Thiago TSS-053 DMSO-d6 250 MHz set27tssH1			***Date***	27 Sep 2012 18:17:52
Date Stamp	27 Sep 2012 18:17:52			***File Name***	C:\USERS\RAFAEL\DESKTOP\ESPECTROS TSS\250 - 2012\SET27TSSH1\1\PDATA\1\1R		
Frequency (MHz)	250.13	***Nucleus***	1H	***Number of Transients***	8	***Origin***	spect
Original Points Count	16384	***Owner***	root	***Points Count***	32768	***Pulse Sequence***	zg30
Receiver Gain	256.00	***SW(cyclical) (Hz)***	5175.98	***Solvent***	DMSO-d6	***Spectrum Offset (Hz)***	1756.2288
Spectrum Type	STANDARD	***Sweep Width (Hz)***	5175.83	***Temperature (degree C)***	25.160		

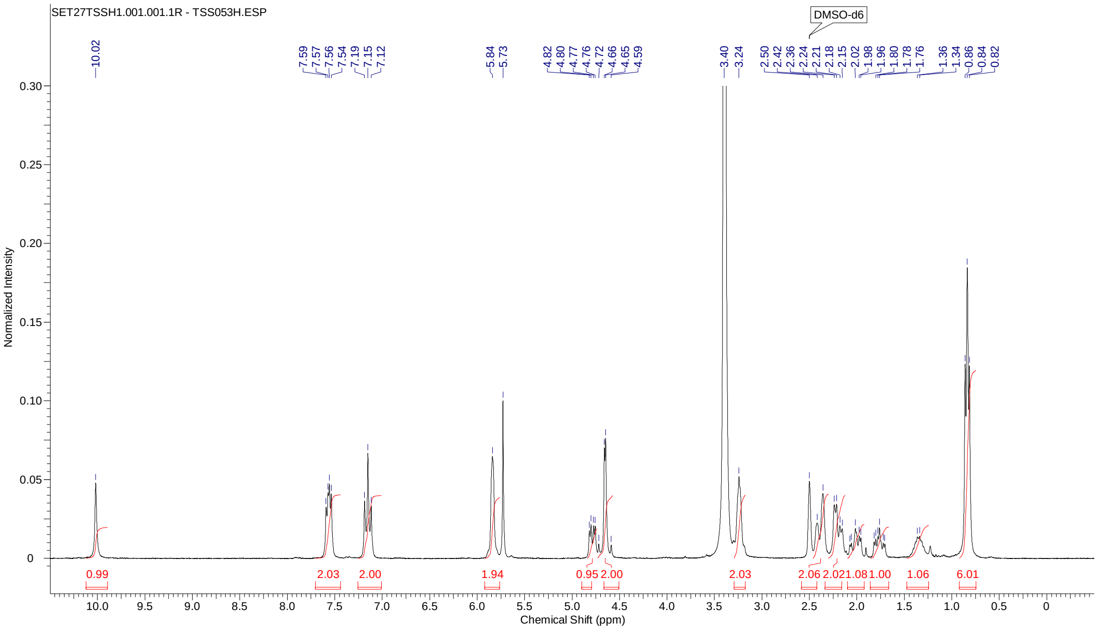

Figure S14 ^{1}H NMR spectrum of compound **3e** (250 MHz, DMSO).

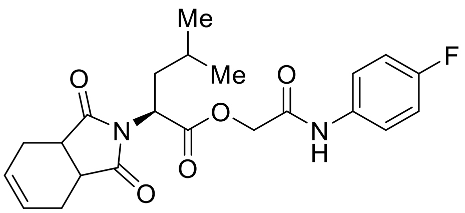

Acquisition Time (sec)	0.5439	***Comment***	Thiago_250Mhz_DMSO_TSS-053_oct02tssC3			***Date***	02 Oct 2012 12:06:40
Date Stamp	02 Oct 2012 12:06:40			***File Name***	C:\USERS\RAFAEL\DESKTOP\ESPECTROS TSS\250 - 2012\OCT02TSSC3\1\FID		
Frequency (MHz)	62.90	***Nucleus***	13C	***Number of Transients***	2191	***Origin***	spect
Original Points Count	8192	***Owner***	root	***Points Count***	8192	***Pulse Sequence***	zgpg30
Receiver Gain	256.00	***SW(cyclical) (Hz)***	15060.24	***Solvent***	DMSO-d6	***Spectrum Offset (Hz)***	6264.0869
Spectrum Type	STANDARD	***Sweep Width (Hz)***	15058.40	***Temperature (degree C)***	25.160		

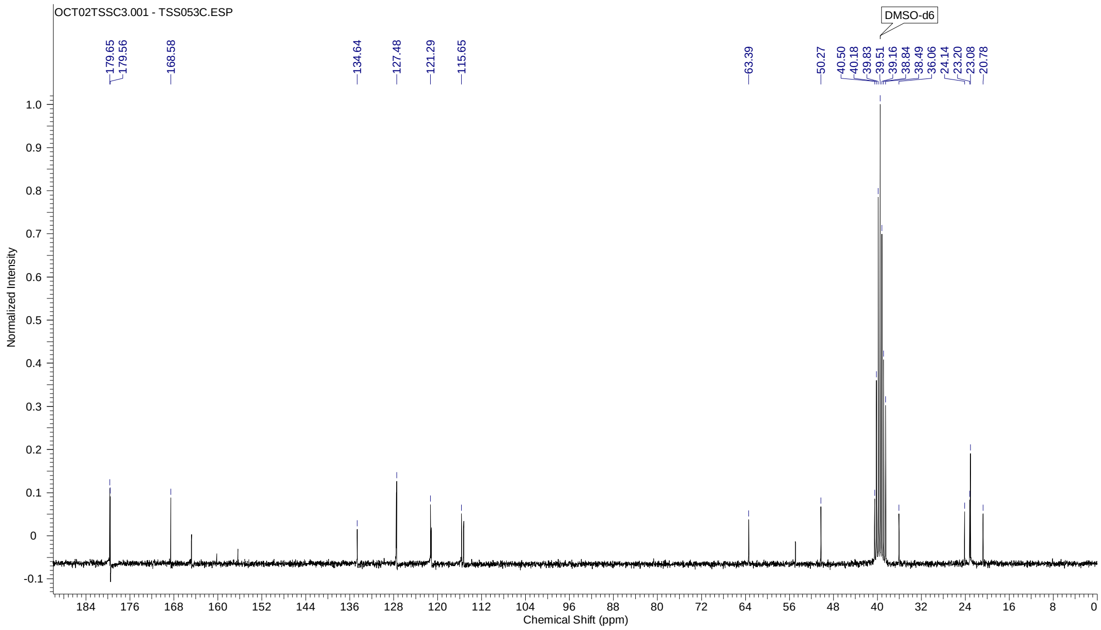

Figure S15 ^{13}C NMR spectrum of compound **3e** (62.5 MHz, DMSO).

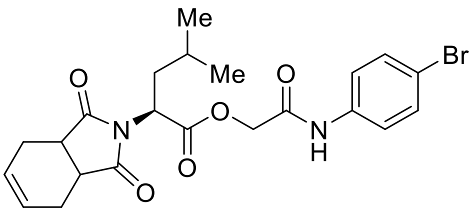

Acquisition Time (sec)	3.1654	***Comment***	Thiago_250MHz_DMSO-d6_TSS-055_out04tssH5			***Date***	04 Oct 2012 20:19:28
Date Stamp	04 Oct 2012 20:19:28			***File Name***	C:\USERS\RAFAEL\DESKTOP\ESPECTROS TSS\250 - 2012\OUT04TSSH5\1\PDATA\1\1R		
Frequency (MHz)	250.13	***Nucleus***	1H	***Number of Transients***	16	***Origin***	spect
Original Points Count	16384	***Owner***	root	***Points Count***	32768	***Pulse Sequence***	zg30
Receiver Gain	456.10	***SW(cyclical) (Hz)***	5175.98	***Solvent***	DMSO-d6	***Spectrum Offset (Hz)***	1755.7549
Spectrum Type	STANDARD	***Sweep Width (Hz)***	5175.83	***Temperature (degree C)***	25.260		

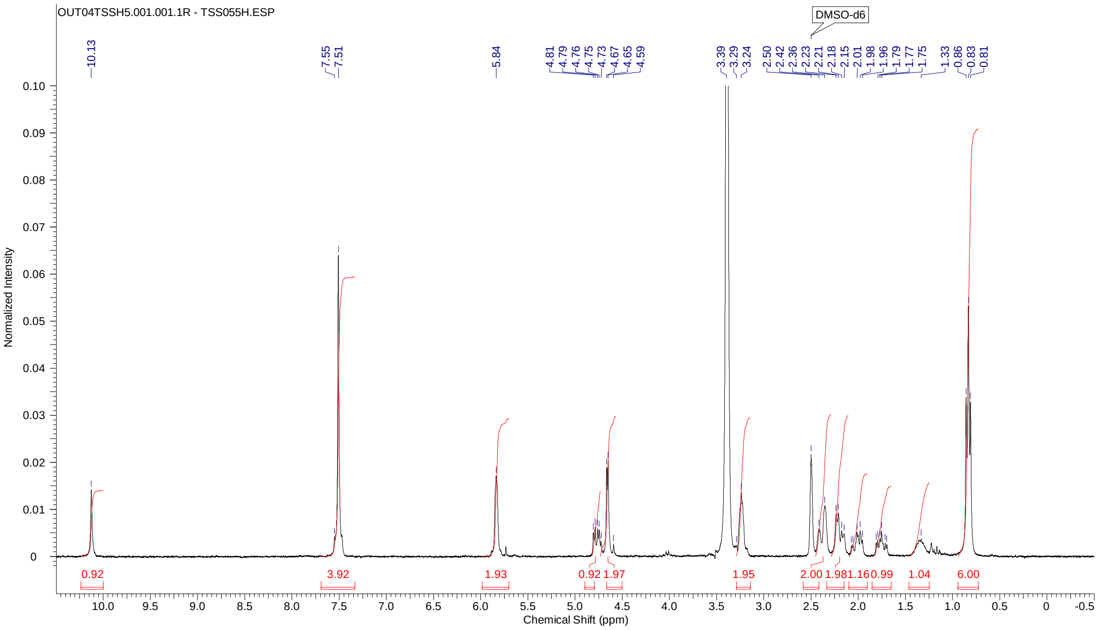

Figure S16 ^{1}H NMR spectrum of compound **3f** (250 MHz, DMSO).

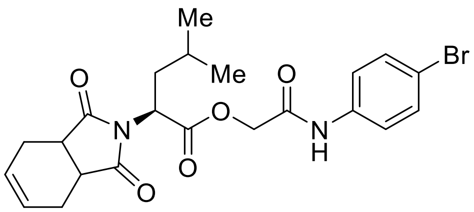

Acquisition Time (sec)	0.5439	Comment	Thiago_250MHz_DMSO-d6_TSS-055_out04tss13C			Date	05 Oct 2012 07:31:28
Date Stamp	05 Oct 2012 07:31:28			File Name	C:\USERS\RAFAEL\DESKTOP\ESPECTROS TSS\250 - 2012\OUT04TSSC5\2\PDATA\1\1R		
Frequency (MHz)	62.90	Nucleus	13C	Number of Transients	10380	Origin	spect
Original Points Count	8192	Owner	root	Points Count	32768	Pulse Sequence	zgpg30
Receiver Gain	287.40	SW(cyclical) (Hz)	15060.24	Solvent	DMSO-d6	Spectrum Offset (Hz)	6265.4478
Spectrum Type	STANDARD	Sweep Width (Hz)	15059.78	Temperature (degree C)	25.160		

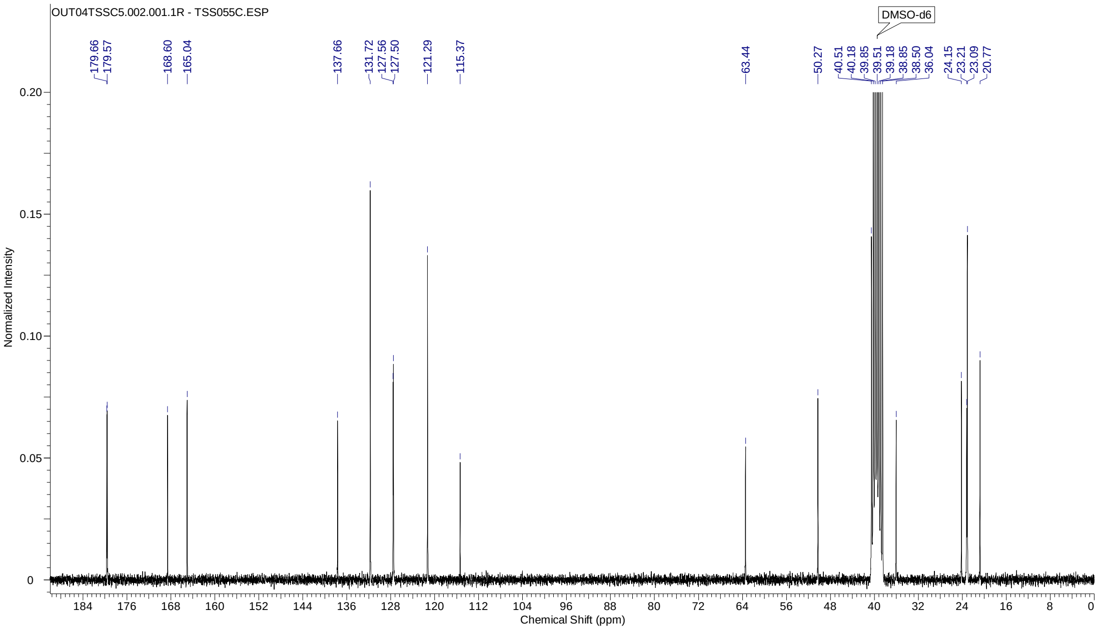

Figure S17 ^{13}C NMR spectrum of compound **3f** (62.5 MHz, DMSO).

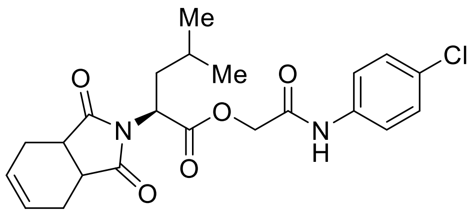

Acquisition Time (sec)	1.5903	***Comment***	Thiago "tss-056" dmso/Av500 out03tssH1			***Date***	03 Oct 2012 14:23:12
Date Stamp	03 Oct 2012 14:23:12			***File Name***	C:\USERS\RAFAEL\DESKTOP\ESPECTROS TSS\500 - 2012\OUT03TSSH1\1\PDATA\1\1R		
Frequency (MHz)	499.87	***Nucleus***	1H	***Number of Transients***	16	***Origin***	spect
Original Points Count	16384	***Owner***	nmrsu	***Points Count***	65536	***Pulse Sequence***	zg30
Receiver Gain	40.30	***SW(cyclical) (Hz)***	10302.20	***Solvent***	DMSO-d6	***Spectrum Offset (Hz)***	3077.8215
Spectrum Type	STANDARD	***Sweep Width (Hz)***	10302.04	***Temperature (degree C)***	25.160		

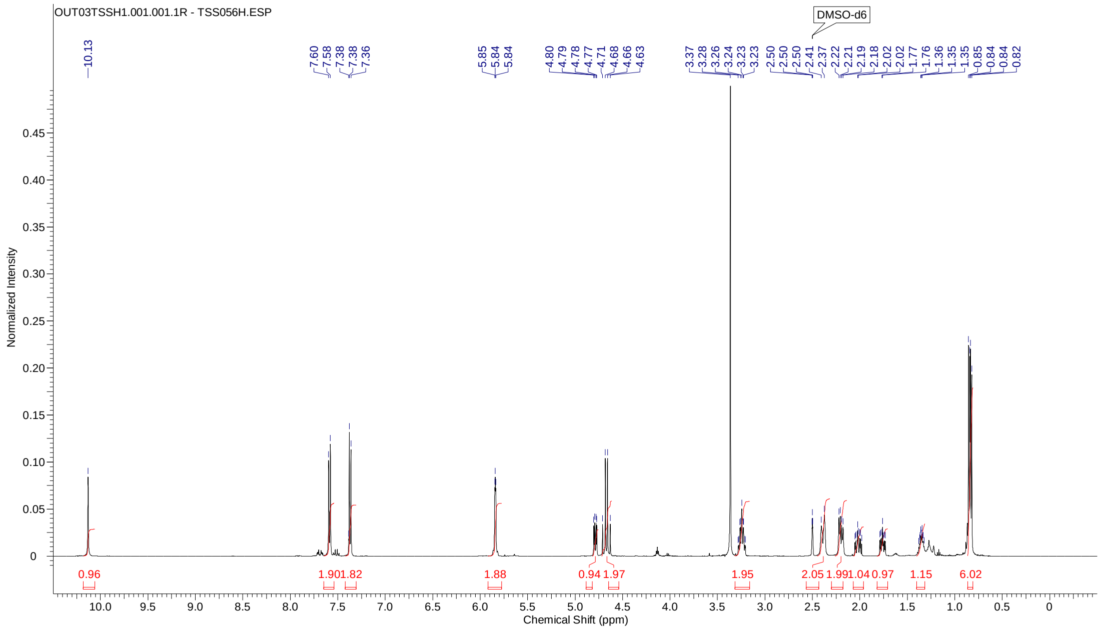

Figure S18 ^{1}H NMR spectrum of compound **3g** (500 MHz, DMSO).

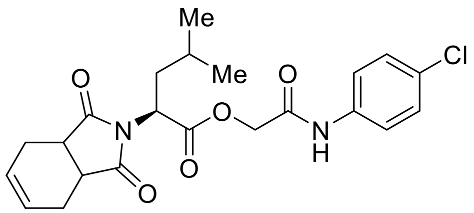

**Acquisition Time (sec)**	0.4981	**Comment**	Thiago "tss-056" dmso/Av500 out03tssC1			**Date**	03 Oct 2012 14:31:44
**Date Stamp**	03 Oct 2012 14:31:44			**File Name**	C:\USERS\RAFAEL\DESKTOP\ESPECTROS TSS\500 - 2012\OUT03TSSH1\2\PDATA\1\1R		
**Frequency (MHz)**	125.69	**Nucleus**	13C	**Number of Transients**	512	**Origin**	spect
**Original Points Count**	16384	**Owner**	nmrsu	**Points Count**	32768	**Pulse Sequence**	zgpg30
**Receiver Gain**	2050.00	**SW(cyclical) (Hz)**	32894.74	**Solvent**	DMSO-d6	**Spectrum Offset (Hz)**	13767.5176
**Spectrum Type**	STANDARD	**Sweep Width (Hz)**	32893.73	**Temperature (degree C)**	25.152		

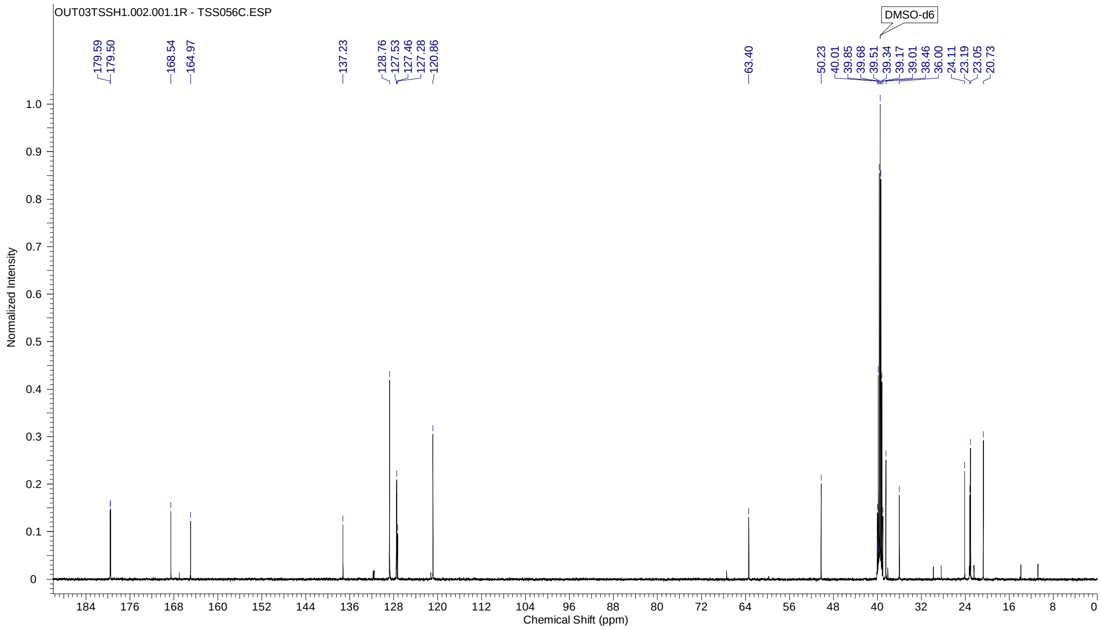

Figure S19 ^{13}C NMR spectrum of compound **3g** (125 MHz, DMSO).

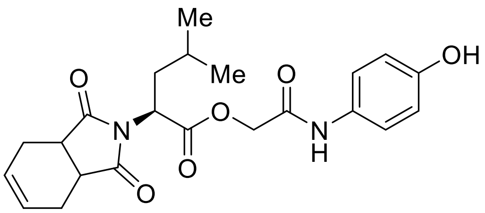

Acquisition Time (sec)	3.1654	***Comment***	Thiago TSS-076 DMSO-d6 250 MHz dez06tssH2			***Date***	06 Dec 2012 18:16:00
Date Stamp	06 Dec 2012 18:16:00						
File Name	C:\USERS\RAFAEL\DESKTOP\ESPECTROS TSS\250 - 2012\DEZ06TSSH2 - TSS076\1\PDATA\1\1R					***Frequency (MHz)***	250.13
Nucleus	1H	***Number of Transients***	32	***Origin***	spect	***Original Points Count***	16384
Owner	root	***Points Count***	32768	***Pulse Sequence***	zg30	***Receiver Gain***	645.10
SW(cyclical) (Hz)	5175.98	***Solvent***	DMSO-d6	***Spectrum Offset (Hz)***	1755.2810	***Spectrum Type***	STANDARD
Sweep Width (Hz)	5175.83	***Temperature (degree C)***	25.160				

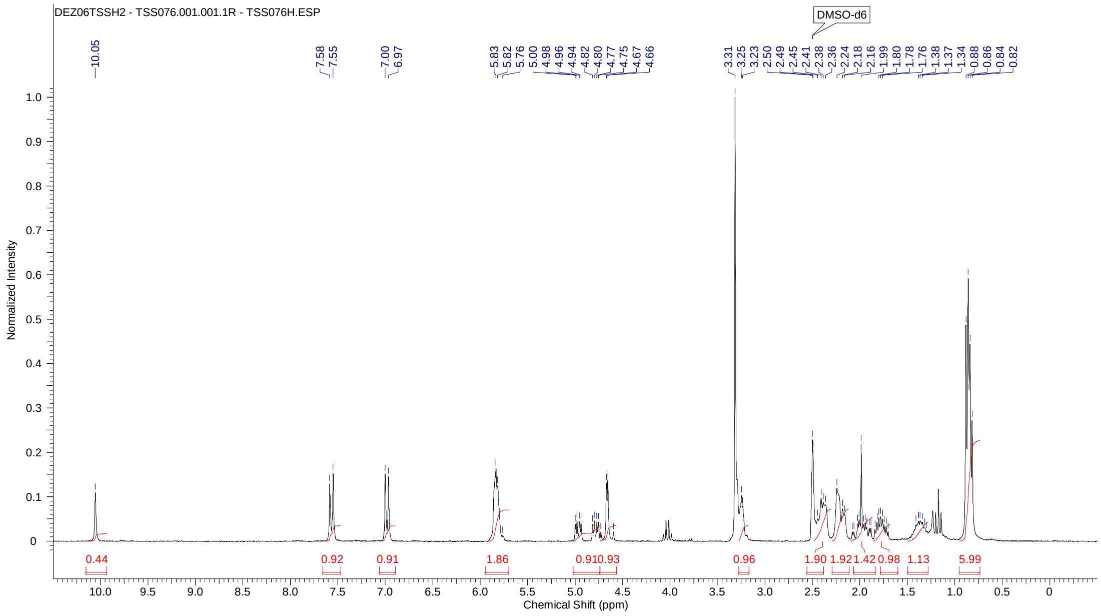

Figure S20 ^{1}H NMR spectrum of compound **3h** (250 MHz, DMSO).

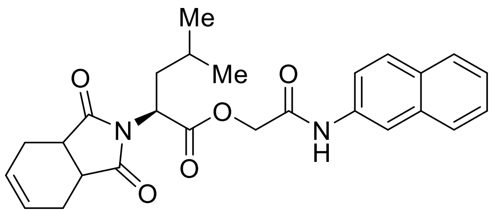

Acquisition Time (sec)	3.1654	*Comment*	Thiago_TSS-095_CDCl3_250MHz_abr18tssH1			*Date*	18 Apr 2013 18:02:56
Date Stamp	18 Apr 2013 18:02:56			*File Name*	C:\USERS\RAFAEL\DESKTOP\ESPECTROS TSS\250 - 2013\ABR18TSSH1\1\PDATA\1\1R		
Frequency (MHz)	250.13	*Nucleus*	1H	*Number of Transients*	16	*Origin*	spect
Original Points Count	16384	*Owner*	root	*Points Count*	32768	*Pulse Sequence*	zg30
Receiver Gain	912.30	*SW(cyclical) (Hz)*	5175.98	*Solvent*	CHLOROFORM-d		
Spectrum Offset (Hz)	1752.9714	*Spectrum Type*	STANDARD	*Sweep Width (Hz)*	5175.83	*Temperature (degree C)*	25.160

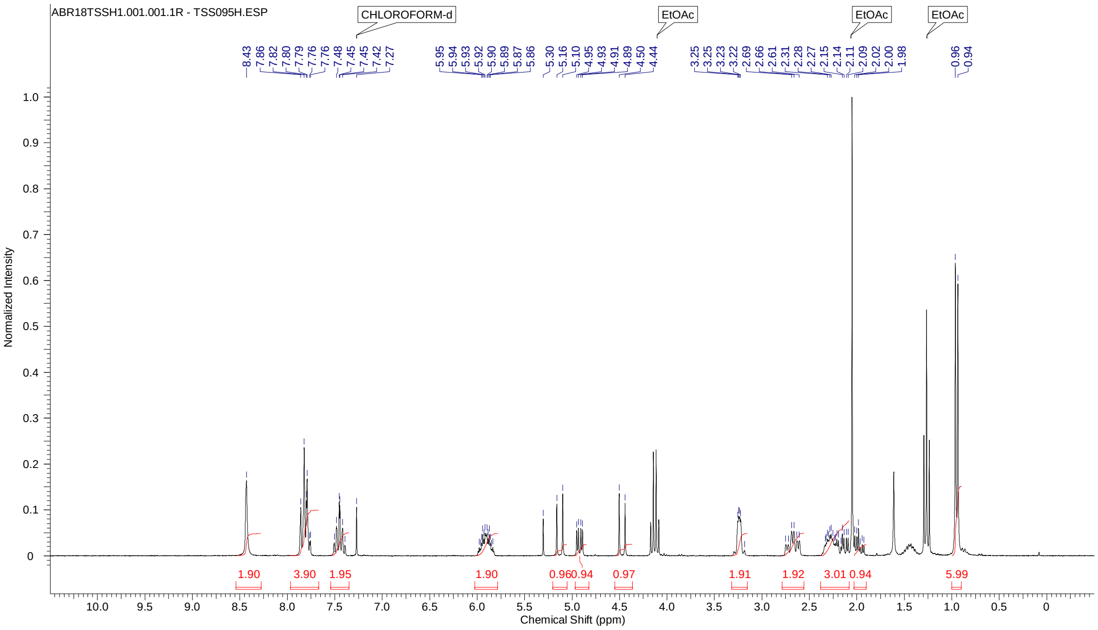

Figure S21 ^{1}H NMR spectrum of compound **3i** (250 MHz, DMSO).

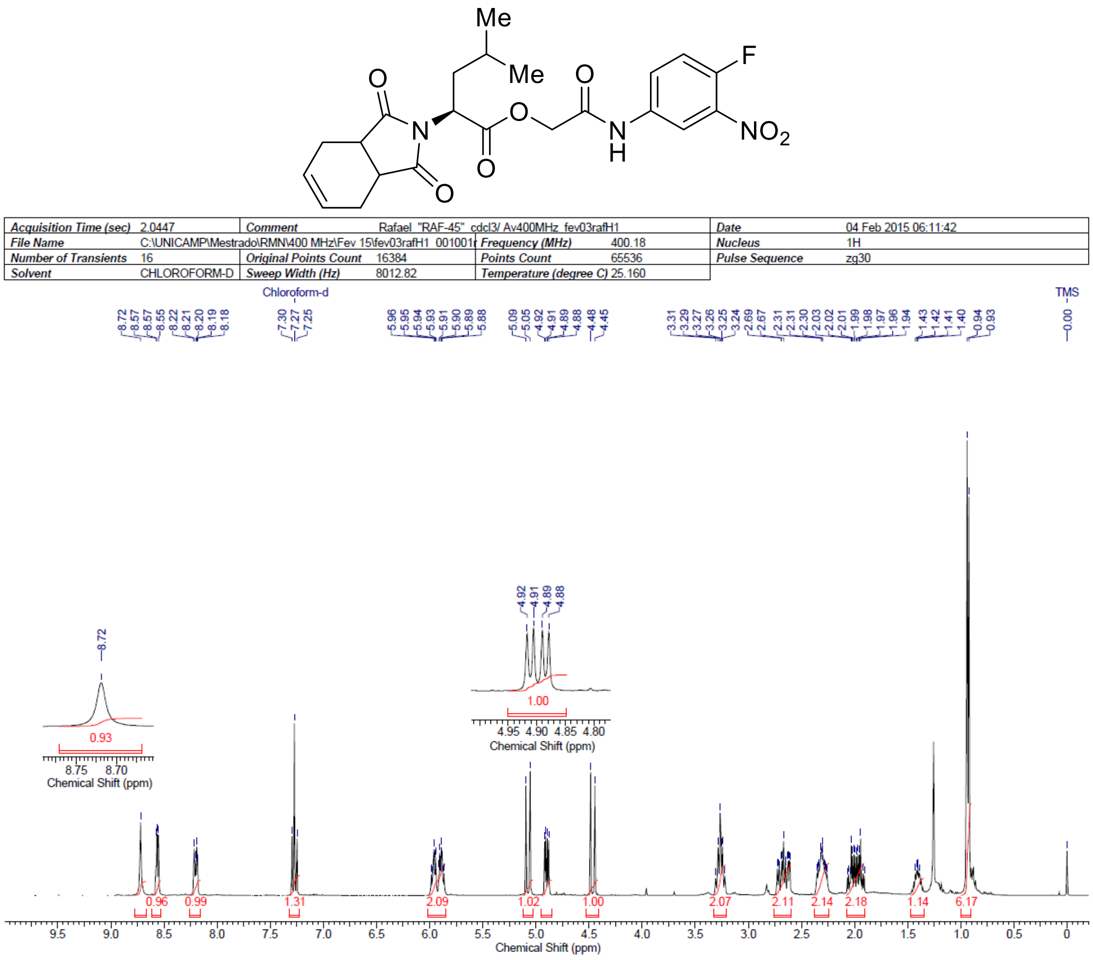

Figure S22 ^{1}H NMR spectrum of compound **3j** (400 MHz, $CDCl_3$).

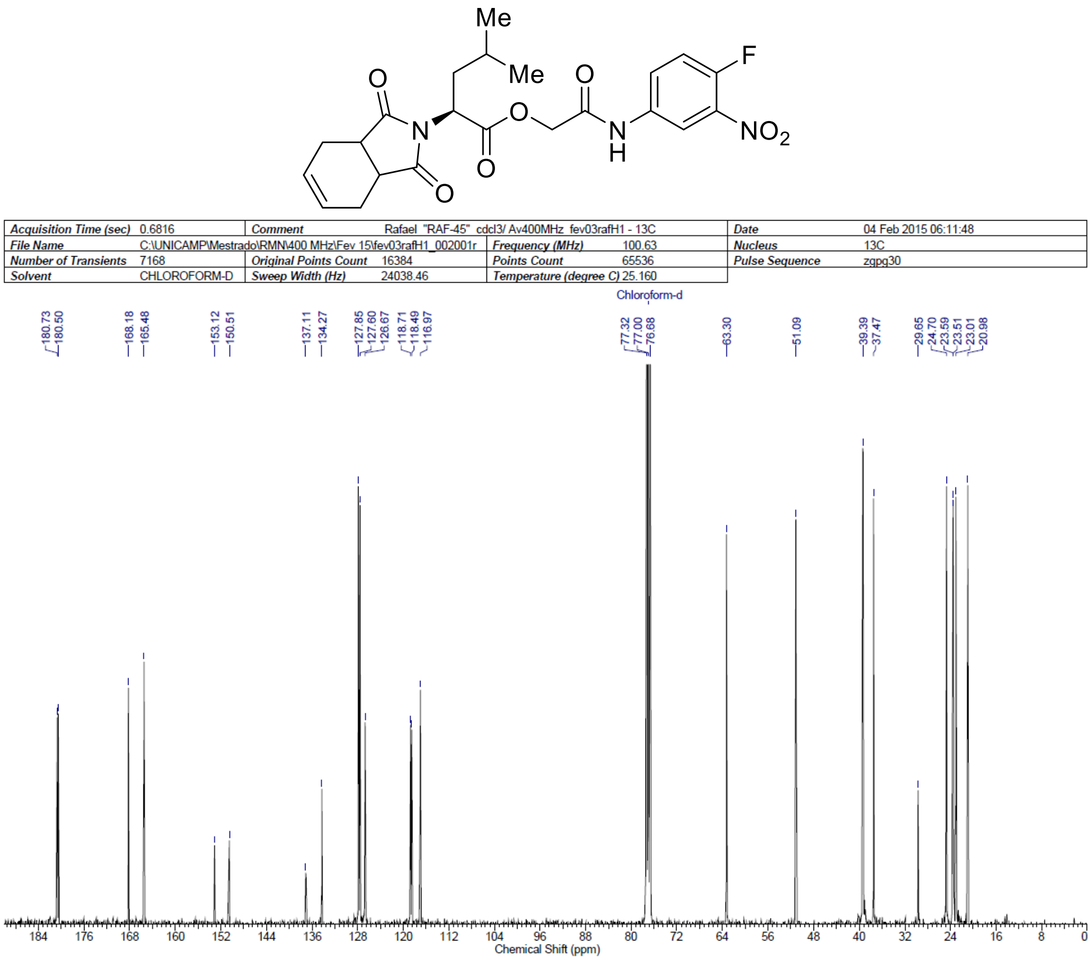

Figure S23 ^{13}C NMR spectrum of compound **3j** (100 MHz, $CDCl_3$).

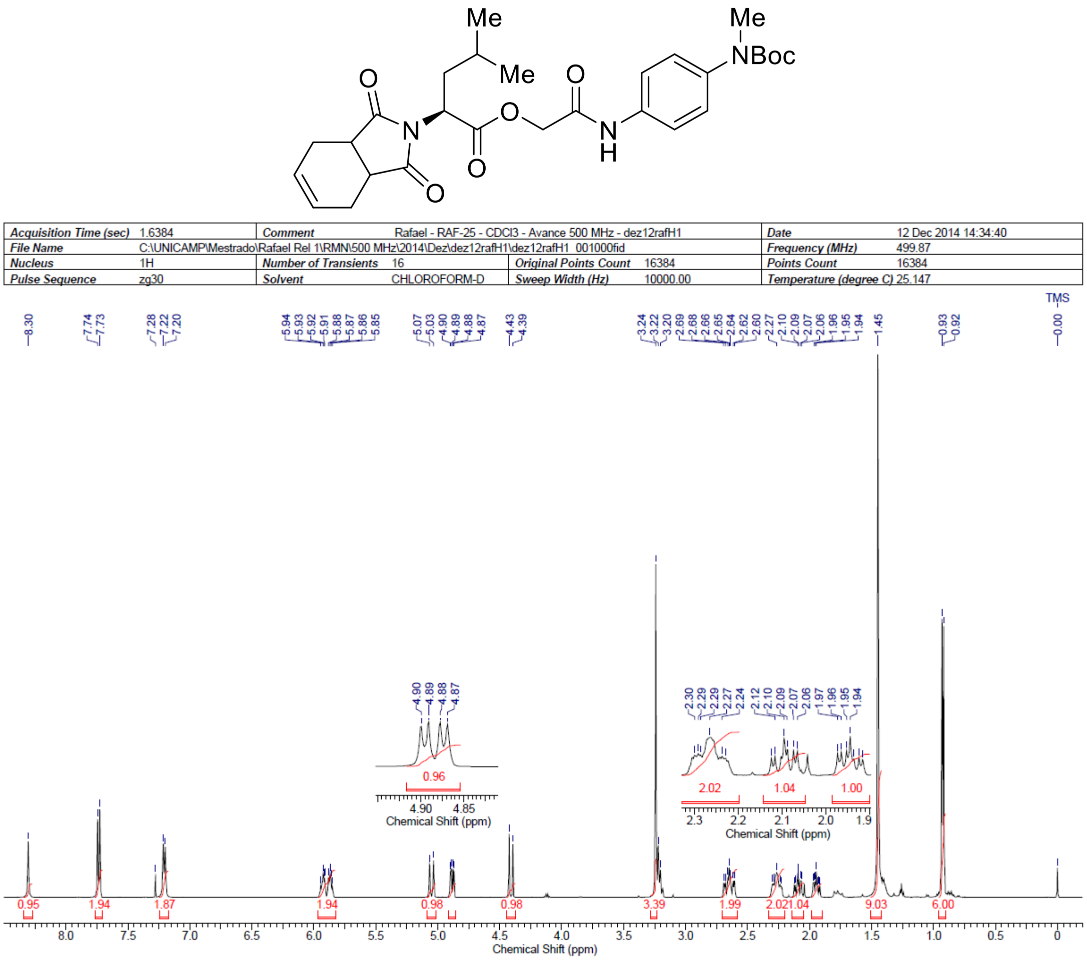

Figure S24 ^{1}H NMR spectrum of compound **3k** (500 MHz, $CDCl_3$).

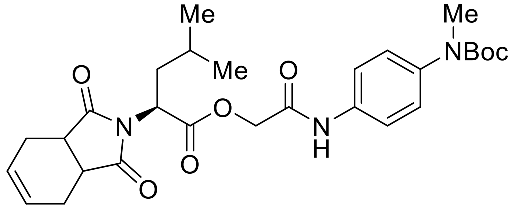

Acquisition Time (sec)	0.5505	Comment	Rafael - RAF-25 - CDCl3 - Avance 500 MHz - dez12rafH1			Date	12 Dec 2014 14:58:08
File Name	C:\UNICAMP\Mestrado\Rafael Rel 1\RMN\500 MHz\2014\Dez\dez12rafH1\dez12rafH1_002000fid					Frequency (MHz)	125.70
Nucleus	13C	Number of Transients	512	Original Points Count	16384	Points Count	16384
Pulse Sequence	zgpg30	Solvent	CHLOROFORM-D	Sweep Width (Hz)	29761.90	Temperature (degree C)	25.150

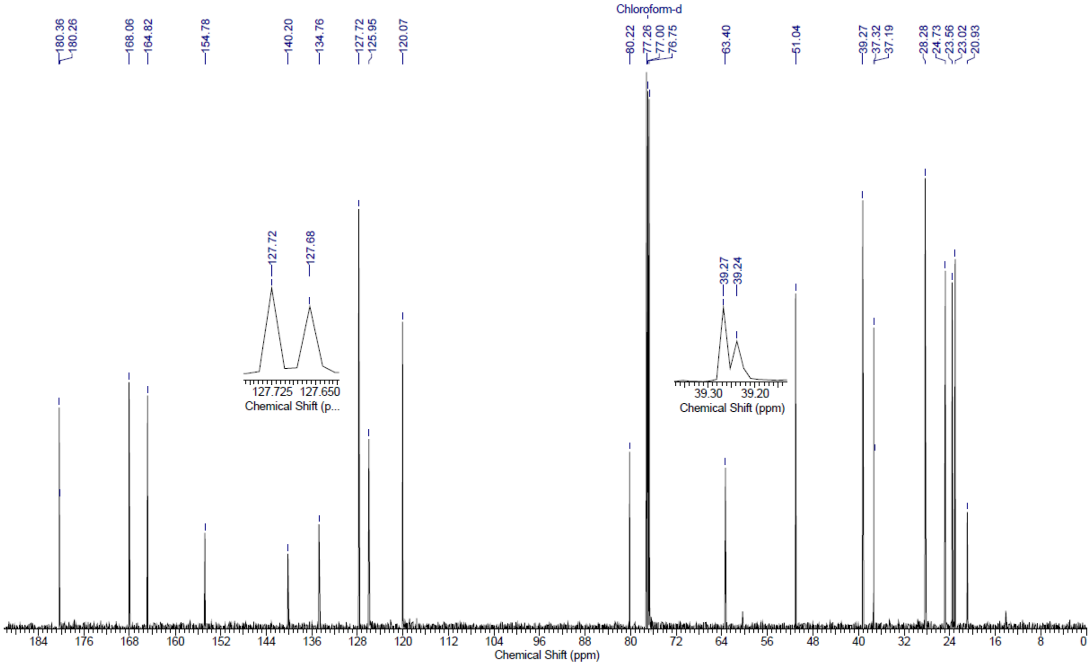

Figure S25 ^{13}C NMR spectrum of compound **3k** (125 MHz, $CDCl_3$).

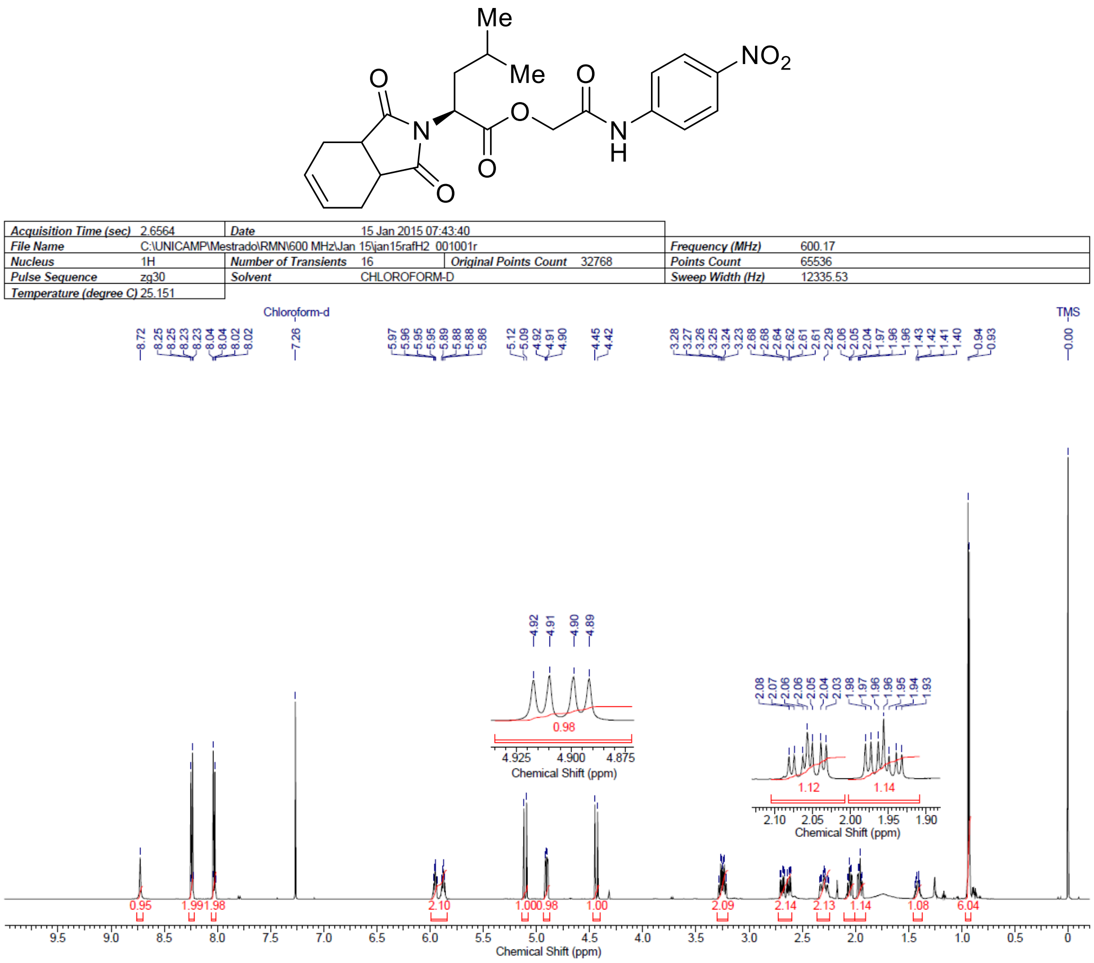

Figure S26 ^{1}H NMR spectrum of compound **3l** (500 MHz, $CDCl_3$).

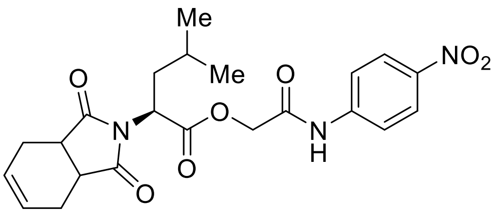

Acquisition Time (sec)	0.4544	Date	15 Jan 2015 08:10:50		
File Name	C:\UNICAMP\Mestrado\RMN\600 MHz\Jan 15\jan15rafH2_002001r			Frequency (MHz)	150.91
Nucleus	13C	Number of Transients	620	Original Points Count 16384	Points Count 32768
Pulse Sequence	zgpg30	Solvent	CHLOROFORM-D	Sweep Width (Hz)	36057.69
Temperature (degree C)	25.147				

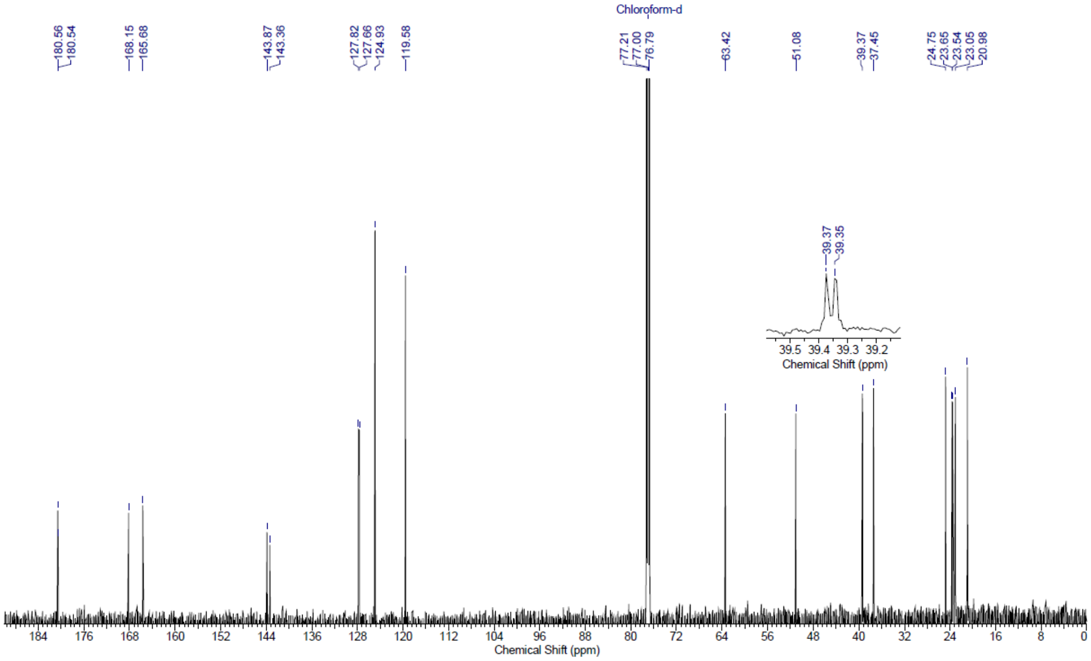

Figure S27 ^{13}C NMR spectrum of compound **3l** (125 MHz, $CDCl_3$).

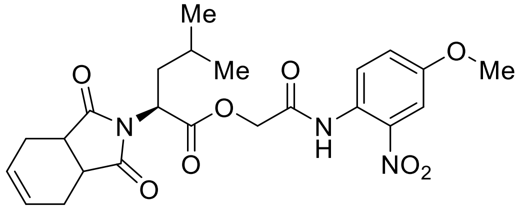

Acquisition Time (sec)	1.6384	Comment	Rafael - RAF-52 - CDCl3 - Avance 500 MHz - fev20rafH1 - 1H			Date	20 Feb 2015 12:09:14
File Name	C:\UNICAMP\Mestrado\RMN\500 MHz\2015\Fev\fev20rafH1_001001r			Frequency (MHz)	499.87	Nucleus	1H
Number of Transients	1	Original Points Count	16384	Points Count	65536	Pulse Sequence	zg30
Solvent	CHLOROFORM-D	Sweep Width (Hz)	10000.00	Temperature (degree C)	25.133		

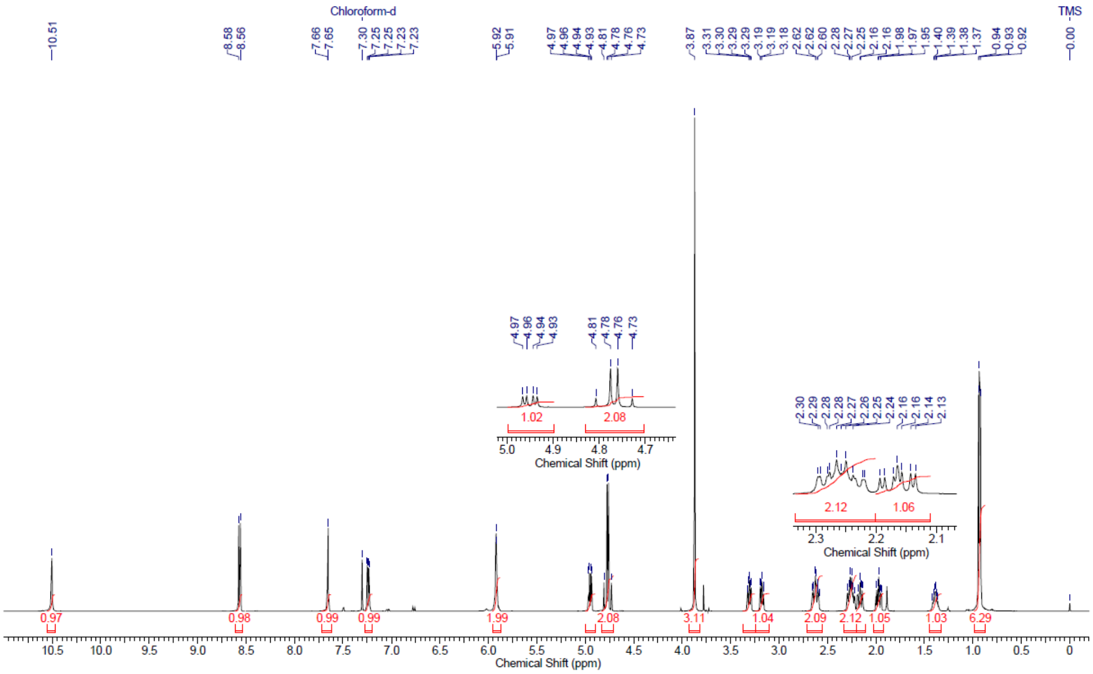

Figure S28 ^{1}H NMR spectrum of compound **3m** (500 MHz, $CDCl_3$).

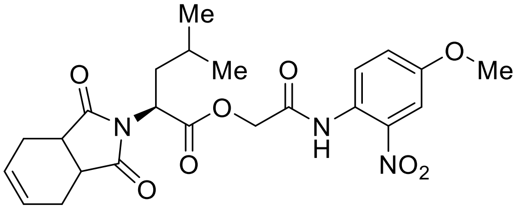

Acquisition Time (sec)	0.5505	Comment	Rafael - RAF-52 - CDCl3 - Avance 500 MHz - fev20rafH1 - 13C			Date	20 Feb 2015 12:10:04
File Name	C:\UNICAMP\Mestrado\RMN\500 MHz\2015\Fev\fev20rafH1_002001r			Frequency (MHz)	125.69	Nucleus	13C
Number of Transients	300	Original Points Count	16384	Points Count	32768	Pulse Sequence	zgpg30
Solvent	CHLOROFORM-D	Sweep Width (Hz)	29761.90	Temperature (degree C)	25.150		

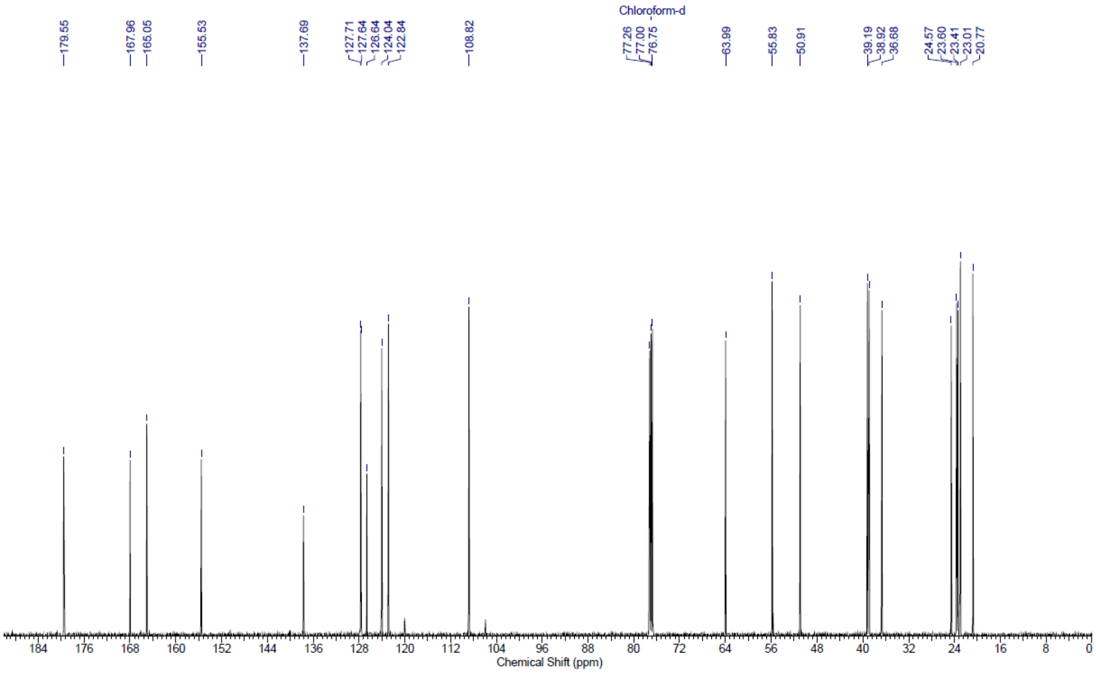

Figure S29 ^{13}C NMR spectrum of compound **3m** (125 MHz, $CDCl_3$).

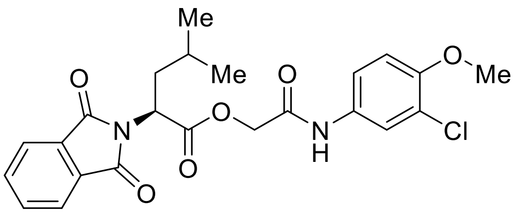

Acquisition Time (sec)	3.1654	Comment	Thiago- TSS 023 - DMSO - Bruker 250 MHz - set25tssH2	Date	25 Sep 2012 14:38:08
Date Stamp	25 Sep 2012 14:38:08			File Name	C:\USERS\RAFAEL\DESKTOP\ESPECTROS TSS\250 - 2012\SET25TSSH2\1\PDATA\1\1R
Frequency (MHz)	250.13	Nucleus	1H	Number of Transients	16	Origin	spect
Original Points Count	16384	Owner	root	Points Count	32768	Pulse Sequence	zg30
Receiver Gain	456.10	SW(cyclical) (Hz)	5175.98	Solvent	DMSO-d6	Spectrum Offset (Hz)	1756.3867
Spectrum Type	STANDARD	Sweep Width (Hz)	5175.83	Temperature (degree C)	25.160		

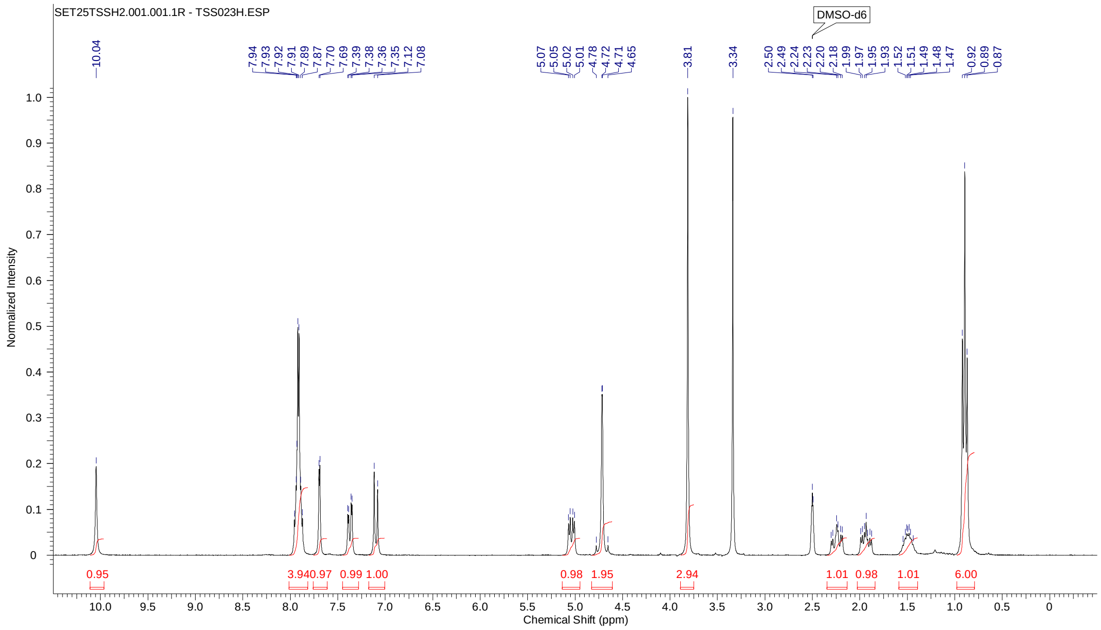

Figure S30 ^{1}H NMR spectrum of compound **10a** (250 MHz, DMSO).

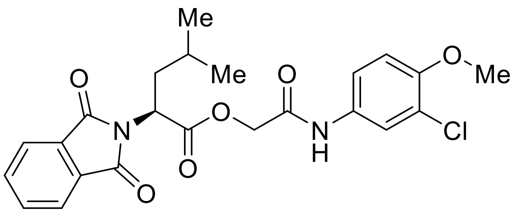

Acquisition Time (sec)	0.5439	***Comment***	Thiago - TSS 023 - DMSO - Bruker 250 MHz - set25tssC2			***Date***	25 Sep 2012 15:29:20
Date Stamp	25 Sep 2012 15:29:20			***File Name***	C:\USERS\RAFAEL\DESKTOP\ESPECTROS TSS\250 - 2012\SET25TSSC2\1\PDATA\1\1R		
Frequency (MHz)	62.90	***Nucleus***	13C	***Number of Transients***	1126	***Origin***	spect
Original Points Count	8192	***Owner***	root	***Points Count***	32768	***Pulse Sequence***	zgpg30
Receiver Gain	512.00	***SW(cyclical) (Hz)***	15060.24	***Solvent***	DMSO-d6	***Spectrum Offset (Hz)***	6260.8516
Spectrum Type	STANDARD	***Sweep Width (Hz)***	15059.78	***Temperature (degree C)***	25.160		

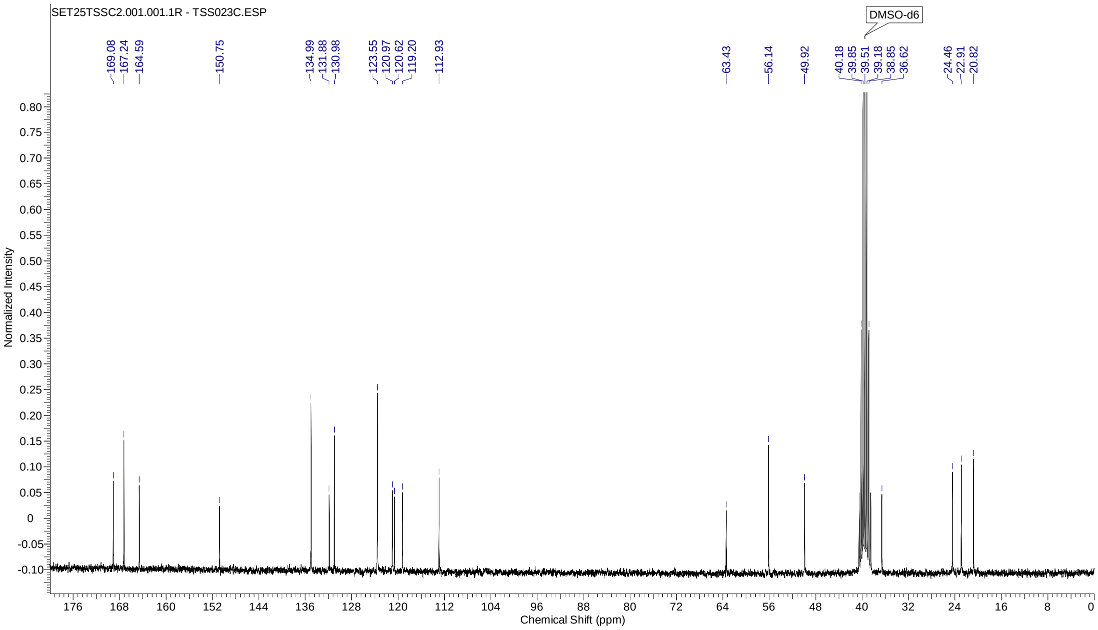

Figure S31 ^{13}C NMR spectrum of compound **10a** (62.5 MHz, DMSO).

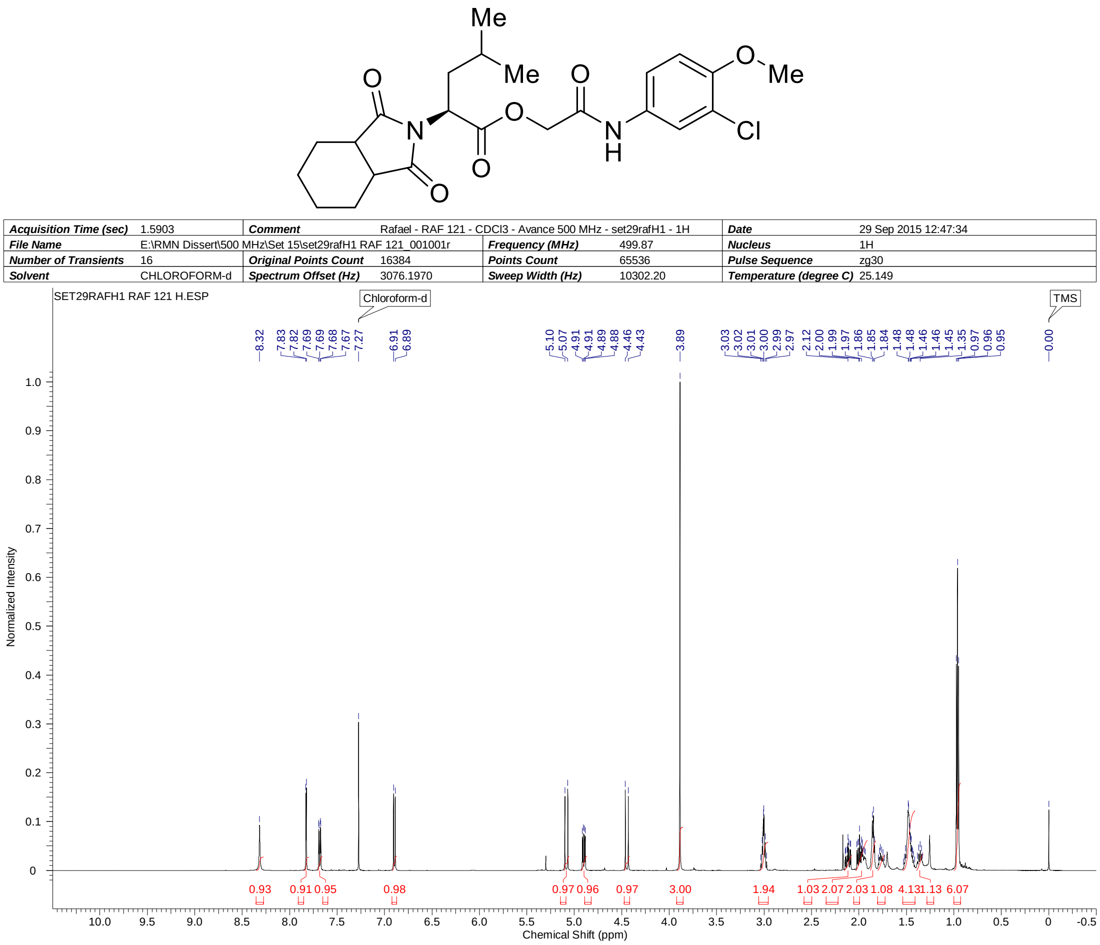

Figure S32 ^{1}H NMR spectrum of compound **10b** (500 MHz, $CDCl_3$).

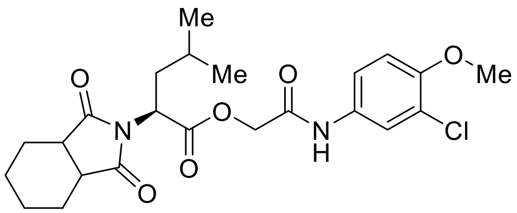

Acquisition Time (sec)	0.4981	***Comment***	Rafael - RAF 121 - CDCl3 - Avance 500 MHz - set29rafH1 - 13C			***Date***	29 Sep 2015 12:49:54
File Name	E:\RMN Dissert\500 MHz\Set 15\set29rafH1 RAF 121_002001r			***Frequency (MHz)***	125.69	***Nucleus***	13C
Number of Transients	171	***Original Points Count***	16384	***Points Count***	32768	***Pulse Sequence***	zgpg30
Solvent	CHLOROFORM-d	***Spectrum Offset (Hz)***	13817.8916	***Sweep Width (Hz)***	32894.74	***Temperature (degree C)***	25.148

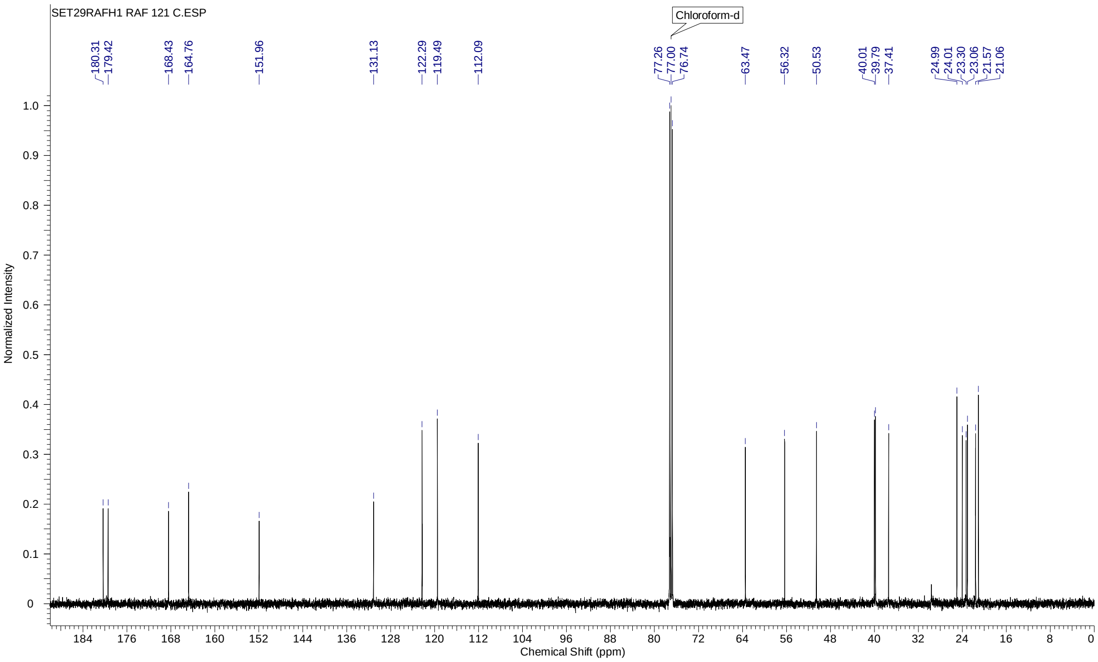

Figure S33 ^{13}C NMR spectrum of compound **10b** (125 MHz, $CDCl_3$).

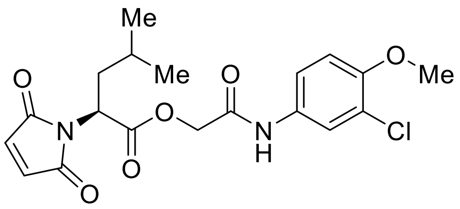

Acquisition Time (sec)	2.0447	***Comment***	Rafael "RAF-114" cdcl3/ Av400MHz nov09rafH1			***Date***	09 Nov 2015 14:09:52
File Name	E:\RMN Dissert\400 MHz\Nov 15\nov09rafH1 RAF 114_RAF 142_001001r					***Frequency (MHz)***	400.18
Nucleus	1H	***Number of Transients***	16	***Original Points Count***	16384	***Points Count***	65536
Pulse Sequence	zg30	***Solvent***	CHLOROFORM-d	***Spectrum Offset (Hz)***	2464.1240	***Sweep Width (Hz)***	8012.82
Temperature (degree C)	25.160						

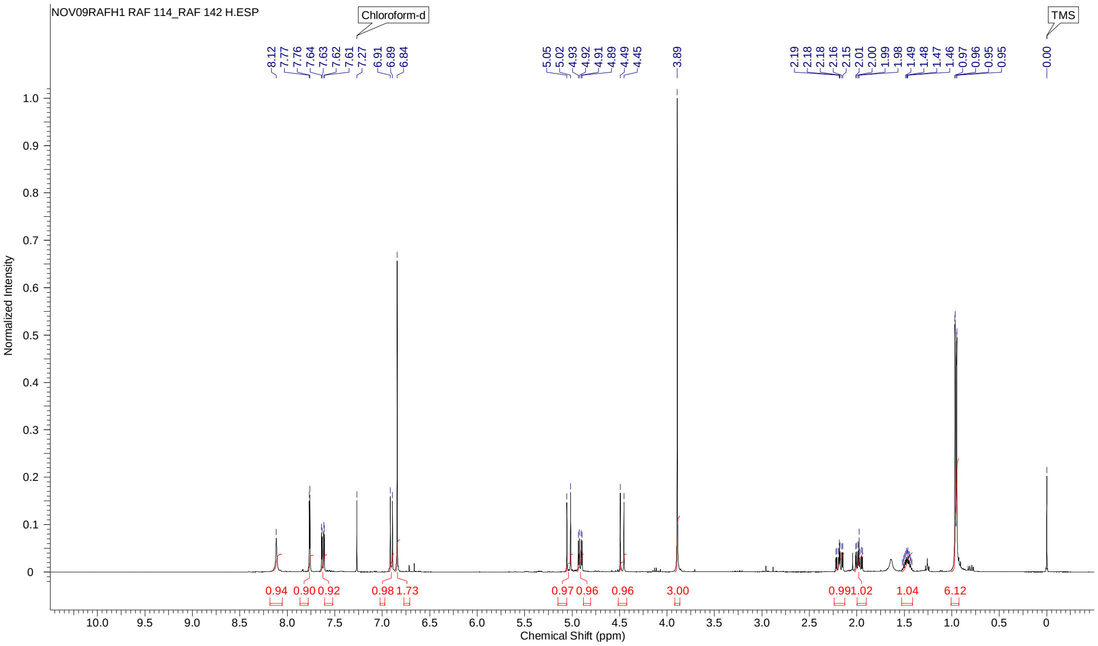

Figure S34 ^{1}H NMR spectrum of compound **10c** (400 MHz, $CDCl_3$).

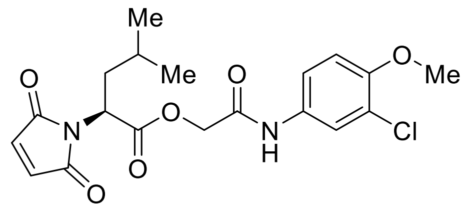

Acquisition Time (sec)	0.6816	***Comment***	Rafael "RAF-114" cdcl3/ Av400MHz nov09rafH1 - 13C			***Date***	09 Nov 2015 15:14:16
File Name	E:\RMN Dissert\400 MHz\Nov 15\nov09rafH1 RAF 114_RAF 142_002001r					***Frequency (MHz)***	100.63
Nucleus	13C	***Number of Transients***	1600	***Original Points Count***	16384	***Points Count***	65536
Pulse Sequence	zgpg30	***Solvent***	CHLOROFORM-d	***Spectrum Offset (Hz)***	10058.0957	***Sweep Width (Hz)***	24038.46
Temperature (degree C)	25.160						

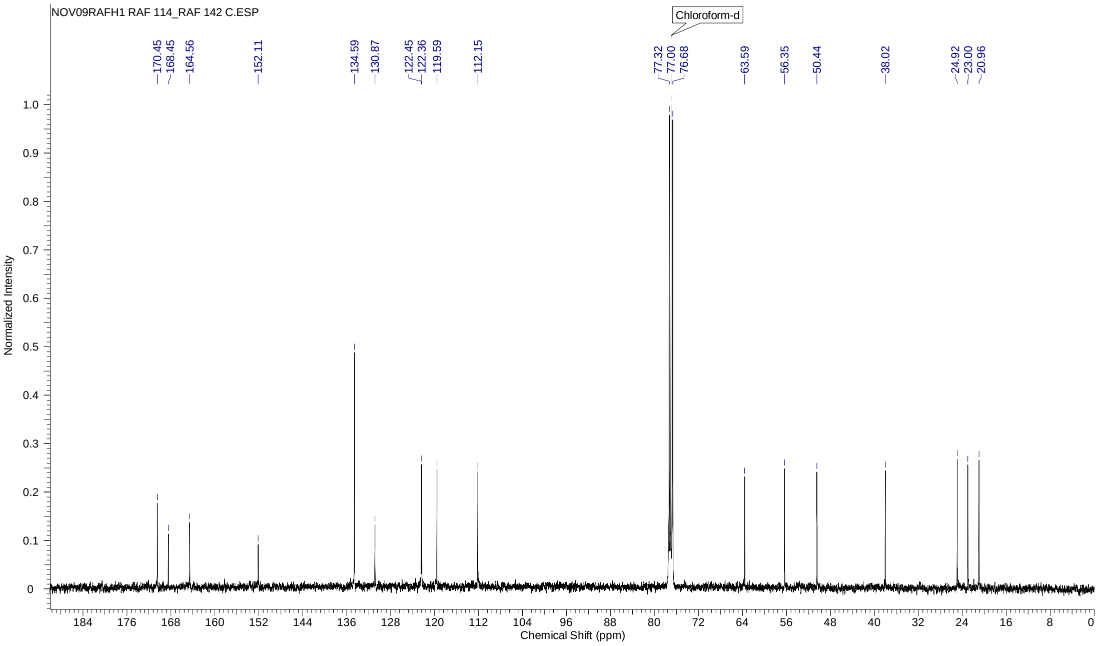

Figure S35 ^{13}C NMR spectrum of compound **10c** (100 MHz, $CDCl_3$).

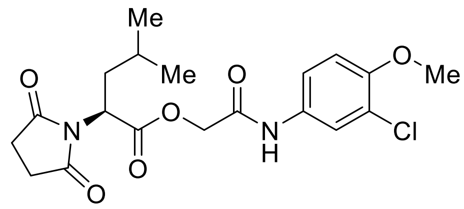

Acquisition Time (sec)	2.0447	Comment	Rafael - RAF 125 - CDCl3 - Avance 400 MHz - out08rafH1 - 1H			Date	08 Oct 2015 08:34:20
File Name	E:\RMN Dissert\400 MHz\Out 15\out08rafH1 RAF 125_001001r			Frequency (MHz)	400.18	Nucleus	1H
Number of Transients	16	Original Points Count	16384	Points Count	65536	Pulse Sequence	zg30
Solvent	CHLOROFORM-D	Sweep Width (Hz)	8012.82	Temperature (degree C)	25.260		

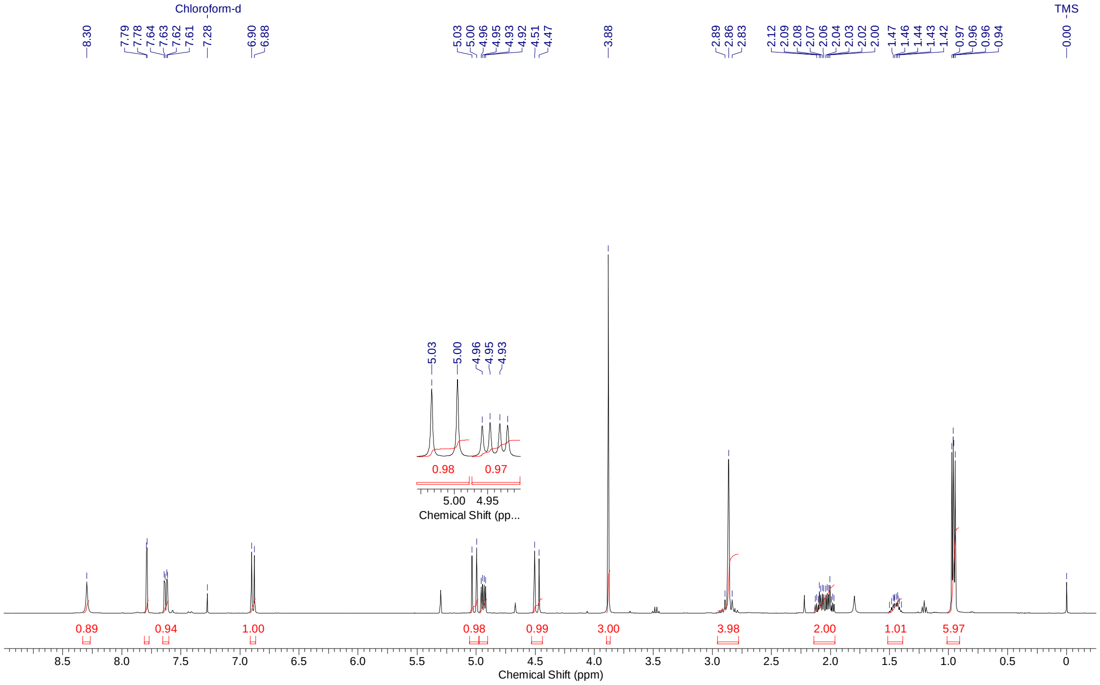

Figure S36 ^{1}H NMR spectrum of compound **10d** (400 MHz, $CDCl_3$).

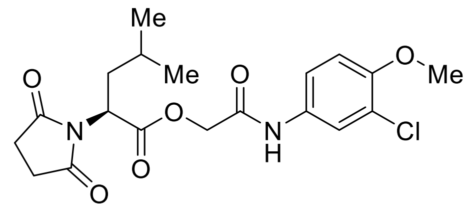

Acquisition Time (sec)	0.6816	***Comment***	Rafael - RAF 125 - CDCl3 - Avance 400 MHz - out08rafH1 - 13C			***Date***	08 Oct 2015 08:35:16
File Name	E:\RMN Dissert\400 MHz\Out 15\out08rafH1 RAF 125_002001r			***Frequency (MHz)***	100.63	***Nucleus***	13C
Number of Transients	647	***Original Points Count***	16384	***Points Count***	65536	***Pulse Sequence***	zgpg30
Solvent	CHLOROFORM-D	***Sweep Width (Hz)***	24038.46	***Temperature (degree C)***	25.160		

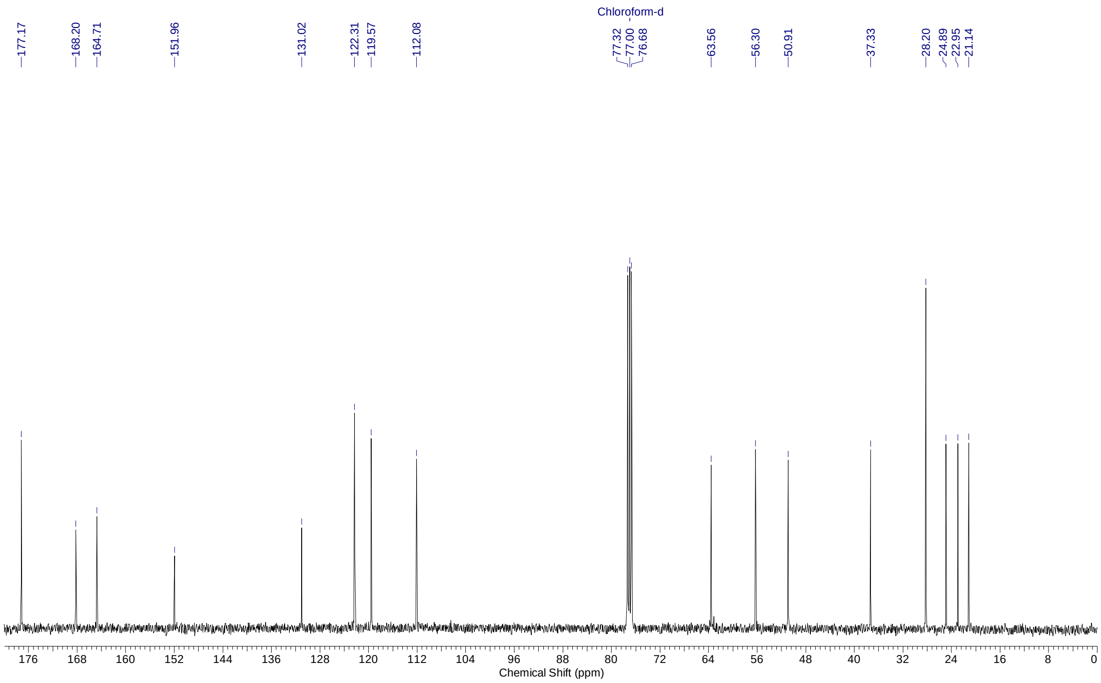

Figure S37 ^{13}C NMR spectrum of compound **10d** (100 MHz, $CDCl_3$).

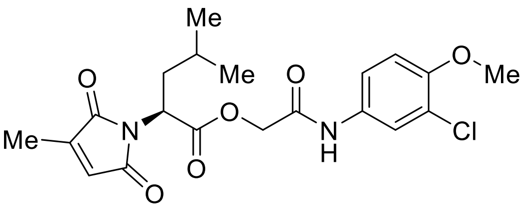

Acquisition Time (sec)	1.5903	*Comment*	Rafael - RAF175 - CDCl3 - Avance 500 MHz - mar24rafH1 - ¹H			*Date*	24 Mar 2016 14:31:32
File Name	E:\RMN Dissert\500 MHz\Mar 16\mar24rafH1 RAF 175_001001r			*Frequency (MHz)*	499.87	*Nucleus*	1H
Number of Transients	16	*Original Points Count*	16384	*Points Count*	65536	*Pulse Sequence*	zg30
Solvent	CHLOROFORM-D	*Sweep Width (Hz)*	10302.20	*Temperature (degree C)*	23.263		

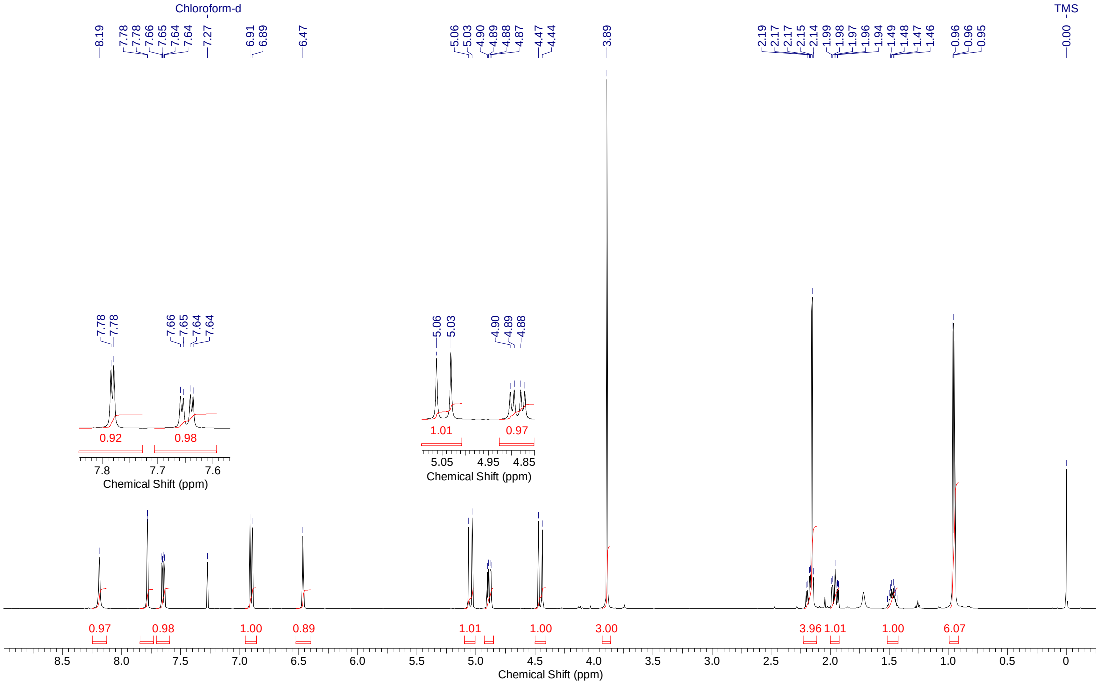

Figure S38 ^{1}H NMR spectrum of compound **10e** (500 MHz, $CDCl_3$).

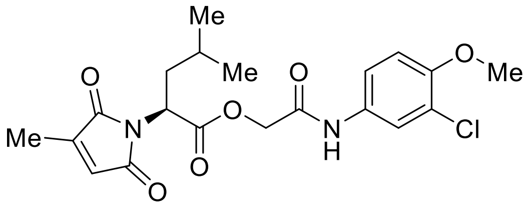

Acquisition Time (sec)	0.4981	***Comment***	Rafael - RAF175 - CDCl3 - Avance 500 MHz - mar24rafH1 - ^{13}C			***Date***	24 Mar 2016 14:43:10
File Name	E:\RMN Dissert\500 MHz\Mar 16\mar24rafH1 RAF 175_002001r			***Frequency (MHz)***	125.69	***Nucleus***	13C
Number of Transients	255	***Original Points Count***	16384	***Points Count***	32768	***Pulse Sequence***	zgpg30
Solvent	CHLOROFORM-D	***Sweep Width (Hz)***	32894.74	***Temperature (degree C)***	24.045		

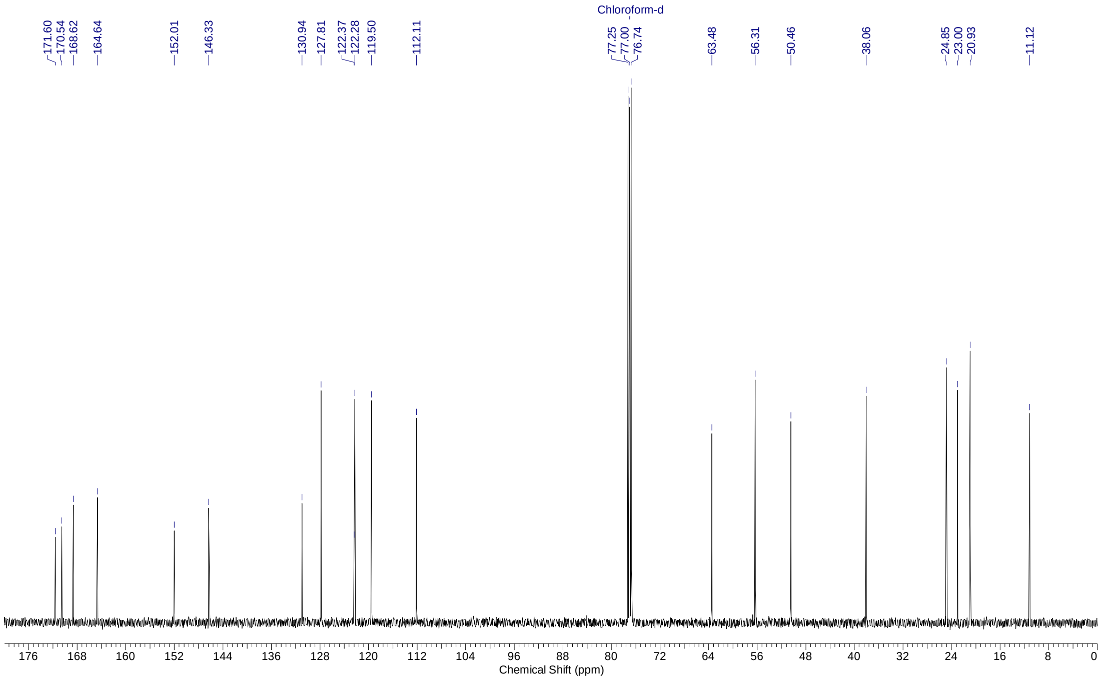

Figure S39 ^{13}C NMR spectrum of compound **10e** (125 MHz, $CDCl_3$).

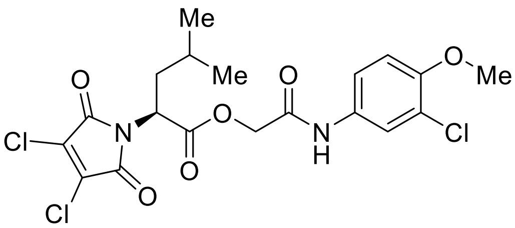

Acquisition Time (sec)	1.5903	***Comment***	Rafael - RAF 187 - CDCl3 - Avance 500 MHz - abr18rafH2 - 1H			***Date***	18 Apr 2016 13:39:50
File Name	E:\RMN Dissert\500 MHz\Abr 16\abr18rafH2 RAF 187_002001r			***Frequency (MHz)***	499.87	***Nucleus***	1H
Number of Transients	16	***Original Points Count***	16384	***Points Count***	65536	***Pulse Sequence***	zg30
Solvent	CHLOROFORM-D	***Sweep Width (Hz)***	10302.20	***Temperature (degree C)***	27.150		

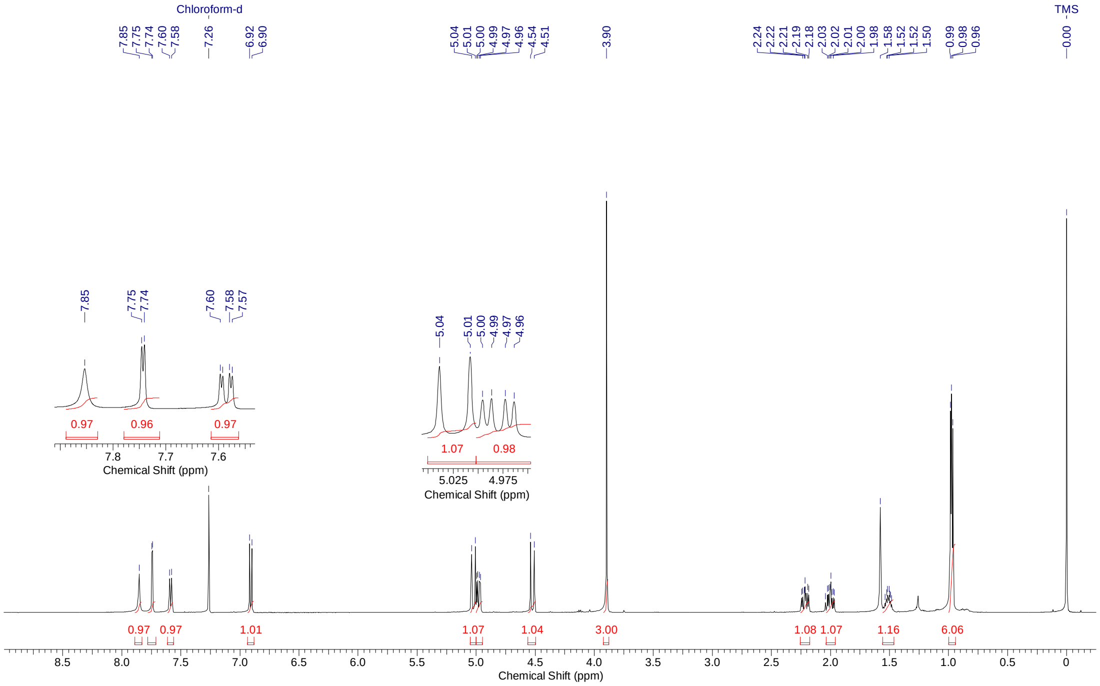

Figure S40 ^{1}H NMR spectrum of compound **10f** (500 MHz, $CDCl_3$).

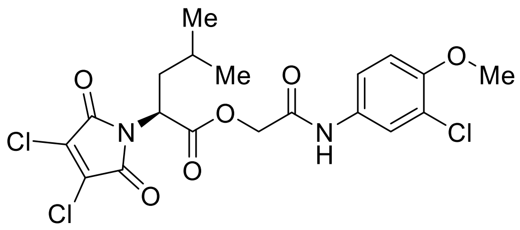

Acquisition Time (sec)	0.4981	***Comment***	Rafael - RAF 187 - CDCl3 - Avance 500 MHz - abr18rafH2 - 13C			***Date***	18 Apr 2016 14:18:14
File Name	E:\RMN Dissert\500 MHz\Abr 16\abr18rafH2 RAF 187_001001r			***Frequency (MHz)***	125.69	***Nucleus***	13C
Number of Transients	1024	***Original Points Count***	16384	***Points Count***	32768	***Pulse Sequence***	zgpg30
Solvent	CHLOROFORM-D	***Sweep Width (Hz)***	32894.74	***Temperature (degree C)***	27.151		

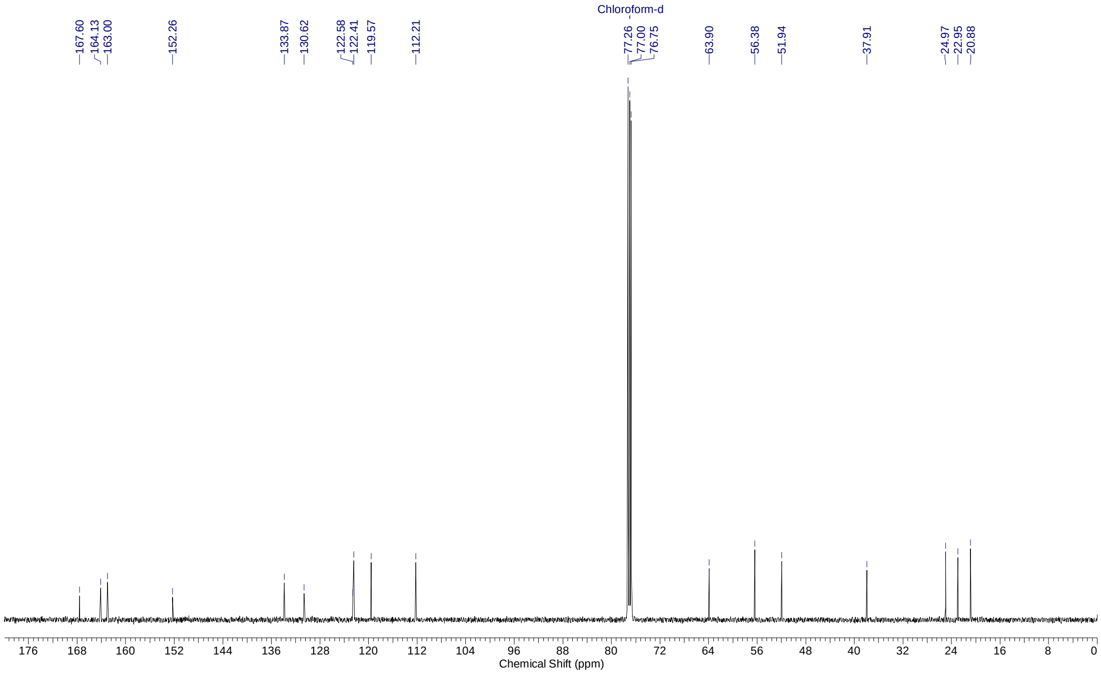

Figure S41 ^{13}C NMR spectrum of compound **10f** (125 MHz, $CDCl_3$).

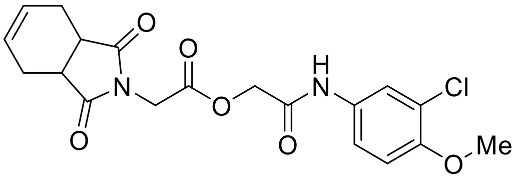

Acquisition Time (sec)	3.1654	***Comment***	Thiago TSS-075 DMSO-d6 250 MHz dez06tssH1	***Date***	06 Dec 2012 18:11:44
Date Stamp	06 Dec 2012 18:11:44				
File Name	C:\USERS\RAFAEL\DESKTOP\ESPECTROS TSS\250 - 2012\DEZ06TSSH1 - TSS075\1\PDATA\1\1R			***Frequency (MHz)***	250.13
Nucleus	1H	***Number of Transients***	32	***Origin*** spect	***Original Points Count*** 16384
Owner	root	***Points Count***	32768	***Pulse Sequence*** zg30	***Receiver Gain*** 724.10
SW(cyclical) (Hz)	5175.98	***Solvent***	DMSO-d6	***Spectrum Offset (Hz)*** 1755.7549	***Spectrum Type*** STANDARD
Sweep Width (Hz)	5175.83	***Temperature (degree C)***	25.160		

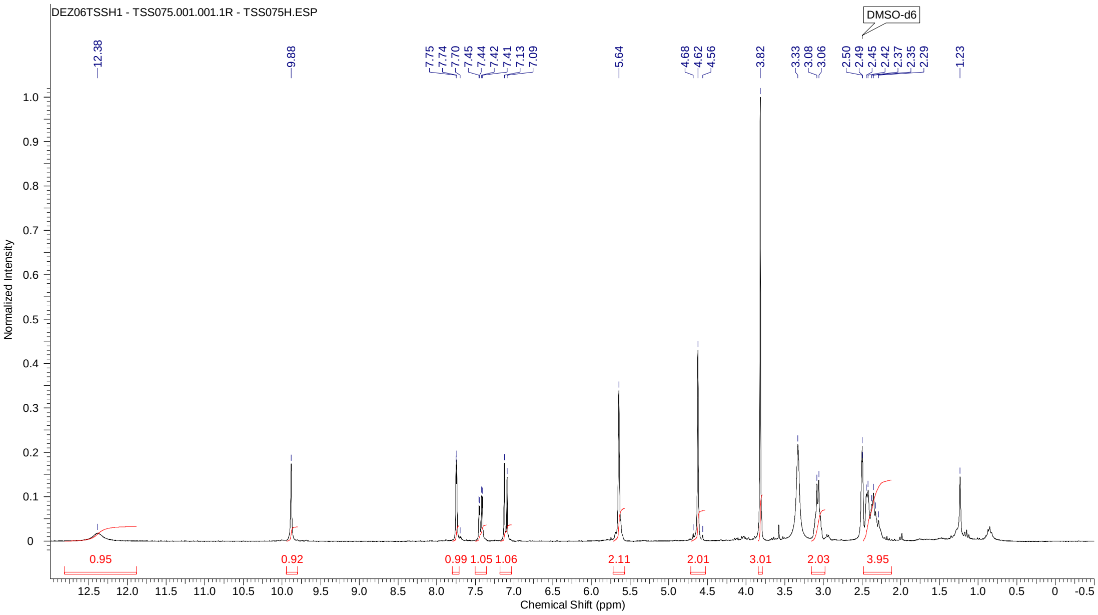

Figure S42 ^{1}H NMR spectrum of compound **10g** (250 MHz, DMSO).

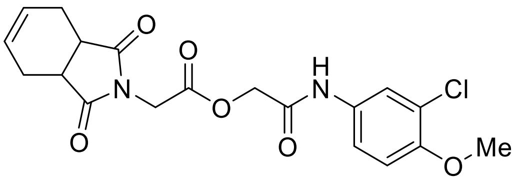

Acquisition Time (sec)	0.5505	Comment	Thiago - tss075 - DMSO - Av 500MHz - dez10tssC1			Date	11 Dec 2012 10:28:48
Date Stamp	11 Dec 2012 10:28:48			File Name	C:\USERS\RAFAEL\DESKTOP\ESPECTROS TSS\500 - 2012\DEZ10TSSC1\1\PDATA\1\1R		
Frequency (MHz)	125.69	Nucleus	13C	Number of Transients	1600	Origin	spect
Original Points Count	16384	Owner	nmrsu	Points Count	32768	Pulse Sequence	zgpg30
Receiver Gain	2050.00	SW(cyclical) (Hz)	29761.90	Solvent	DMSO-d6	Spectrum Offset (Hz)	12508.7627
Spectrum Type	STANDARD	Sweep Width (Hz)	29761.00	Temperature (degree C)	25.143		

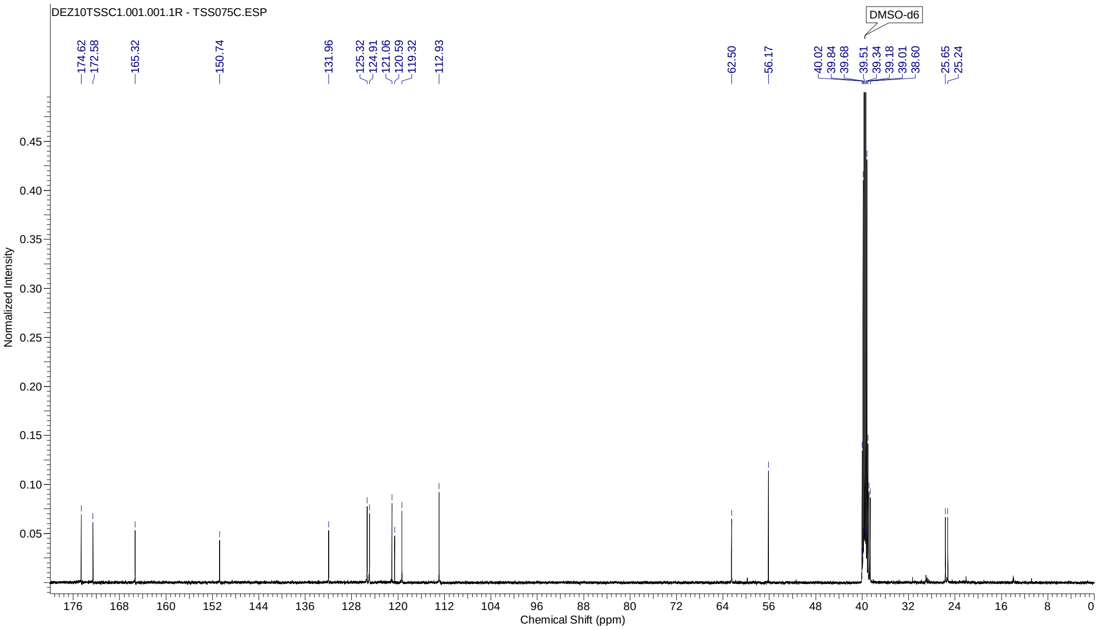

Figure S43 ^{13}C NMR spectrum of compound **10g** (62.5 MHz, DMSO).

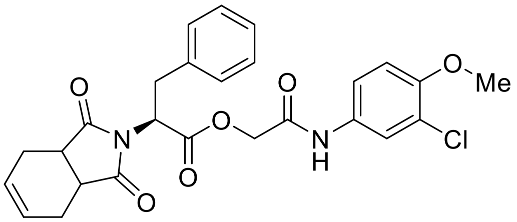

Acquisition Time (sec)	3.1654	***Comment***	jul08rafH1 - RAF 99 CDCl3 250 MHz			***Date***	08 Jul 2016 15:55:24
File Name	C:\Users\Rafael\Dropbox\Mestrado\Dissertação\RMN Dissert\jul08rafH1_001001r					***Frequency (MHz)***	250.13
Nucleus	1H	***Number of Transients***	16	***Original Points Count***	16384	***Points Count***	32768
Pulse Sequence	zg30	***Solvent***	CHLOROFORM-D	***Sweep Width (Hz)***	5175.98	***Temperature (degree C)***	25.160

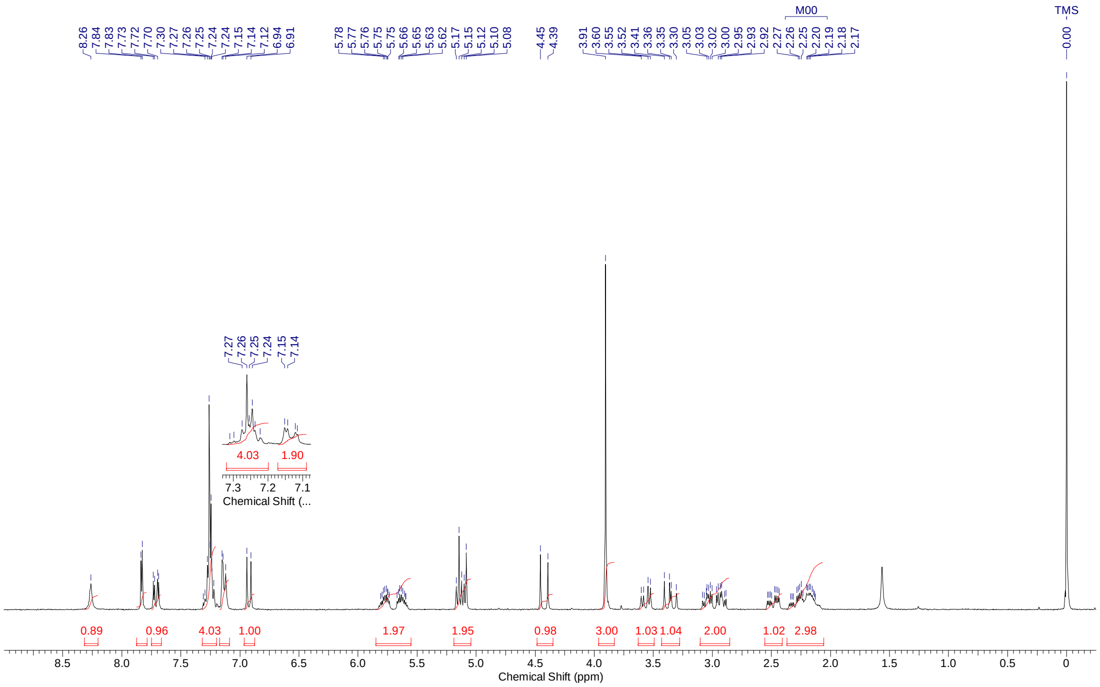

Figure S44 ^{1}H NMR spectrum of compound **10h** (250 MHz, $CDCl_3$).

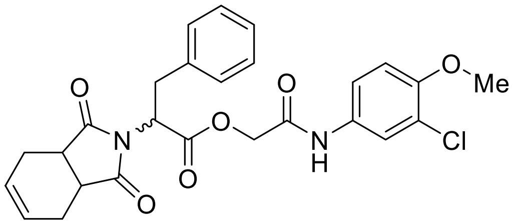

Acquisition Time (sec)	1.5903	*Comment*	Rafael - RAF-111 - CDCl3 - Avance 500 MHz - set18rafH3 - ¹H			*Date*	18 Sep 2015 15:41:22
File Name	E:\RMN Dissert\500 MHz\Set 15\set18rafH3 RAF 111_001001r			*Frequency (MHz)*	499.87	*Nucleus*	1H
Number of Transients	8	*Original Points Count*	16384	*Points Count*	65536	*Pulse Sequence*	zg30
Solvent	CHLOROFORM-D	*Sweep Width (Hz)*	10302.20	*Temperature (degree C)*	25.163		

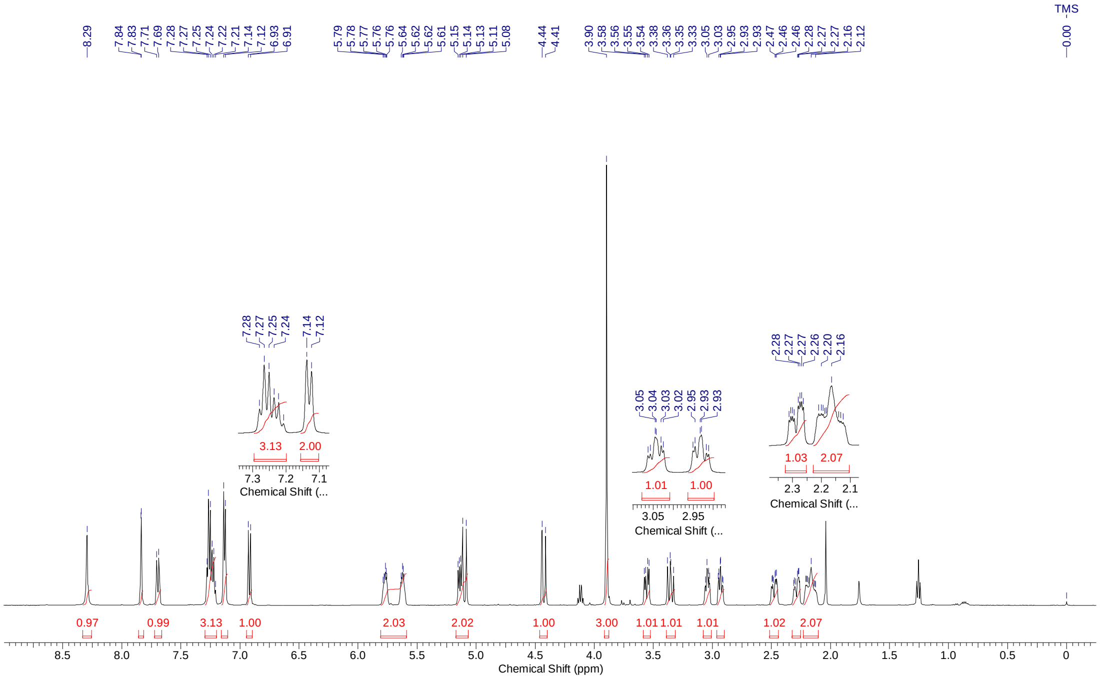

Figure S45 ^{1}H NMR spectrum of compound **$10h_{rac}$** (500 MHz, $CDCl_3$).

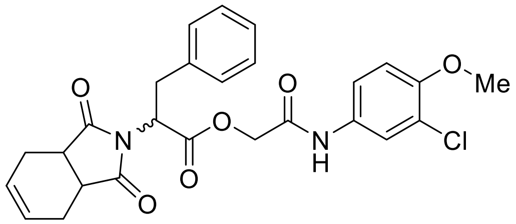

Acquisition Time (sec)	0.4981	***Comment***	Rafael - RAF-111 - CDCl3 - Avance 500 MHz - set18rafH3 - ¹³C			***Date***	18 Sep 2015 16:13:02
File Name	E:\RMN Dissert\500 MHz\Set 15\set18rafH3 RAF 111_002001r			***Frequency (MHz)***	125.69	***Nucleus***	13C
Number of Transients	256	***Original Points Count***	16384	***Points Count***	32768	***Pulse Sequence***	zgpg30
Solvent	CHLOROFORM-D	***Sweep Width (Hz)***	32894.74	***Temperature (degree C)***	25.140		

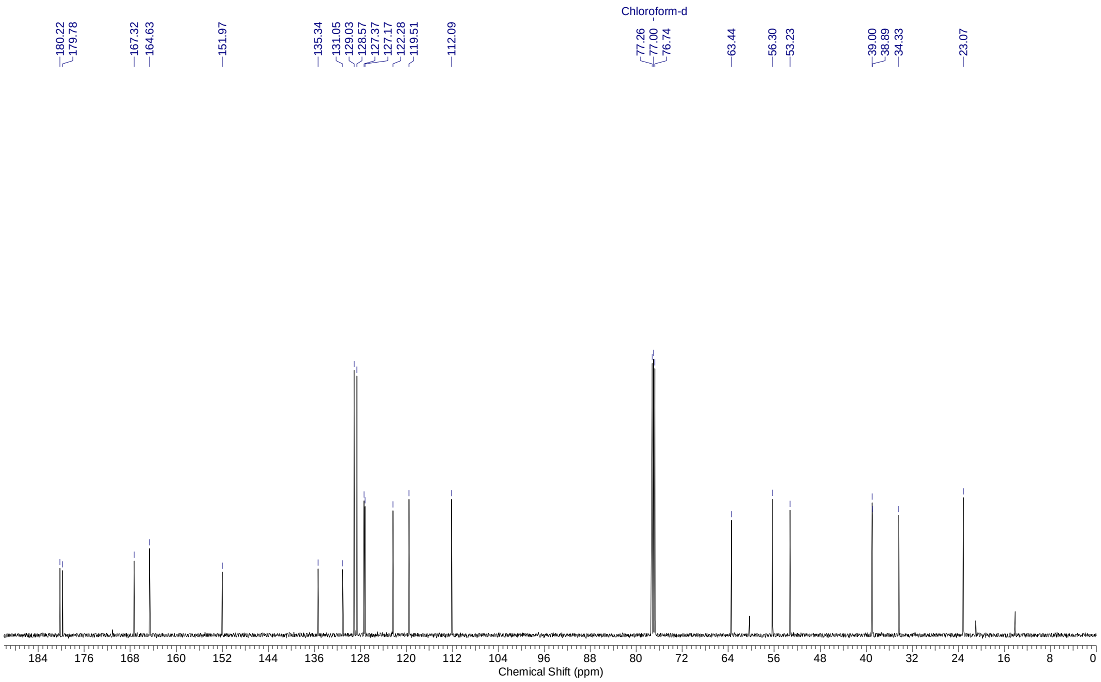

Figure S46 ^{13}C NMR spectrum of compound **10h$_{rac}$** (125 MHz, $CDCl_3$).

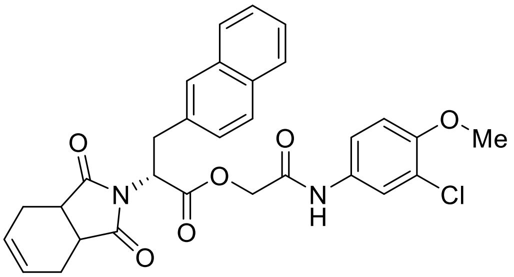

Acquisition Time (sec)	2.0447	*Date*	16 Sep 2015 10:33:30				
File Name	E:\RMN Dissert\400 MHz\Set 15\set16rafH1 RAF 110_001001r					*Frequency (MHz)*	400.18
Nucleus	1H	*Number of Transients*	16	*Original Points Count*	16384	*Points Count*	65536
Pulse Sequence	zg30	*Solvent*	CHLOROFORM-D			*Sweep Width (Hz)*	8012.82
Temperature (degree C)	25.260						

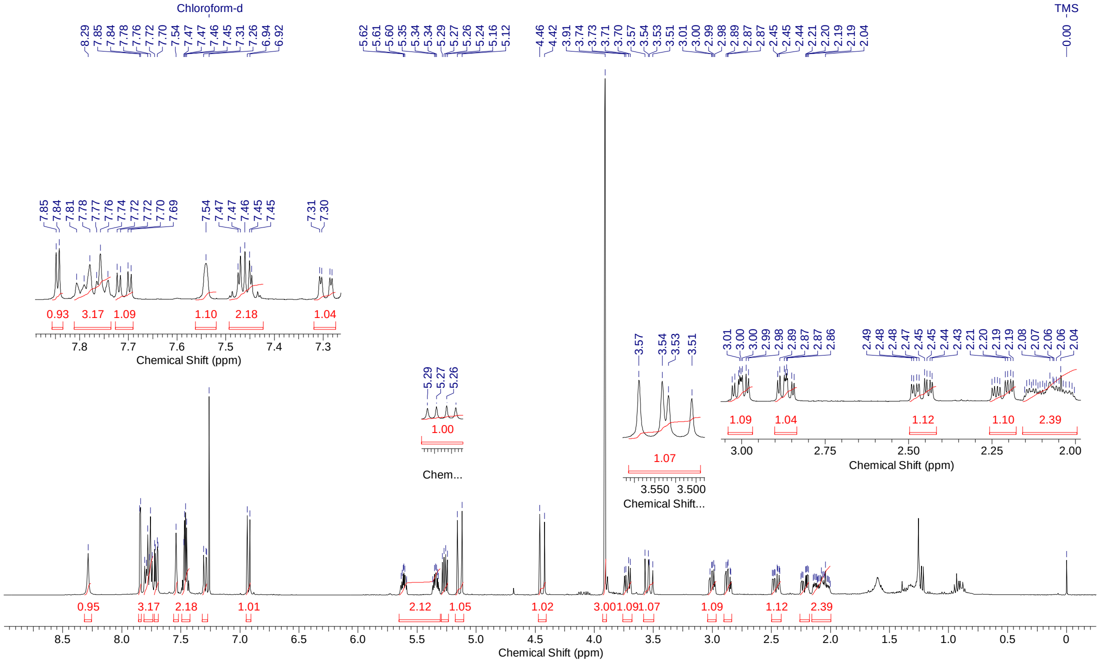

Figure S47 ^{1}H NMR spectrum of compound **10i** (400 MHz, $CDCl_3$).

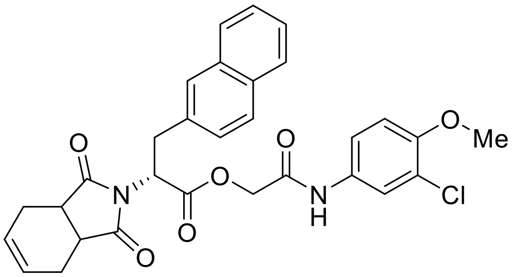

Acquisition Time (sec)	0.6816	Comment	Rafael - RAF - 110 - CDCl3 - Av 500MHz - set16rafH1 13C			Date	16 Sep 2015 13:23:58
File Name	E:\RMN Dissert\400 MHz\Set 15\set16rafH1 RAF 110_002001r			Frequency (MHz)	100.63	Nucleus	13C
Number of Transients	5120	Original Points Count	16384	Points Count	65536	Pulse Sequence	zgpg30
Solvent	CHLOROFORM-D	Sweep Width (Hz)	24038.46	Temperature (degree C)	25.160		

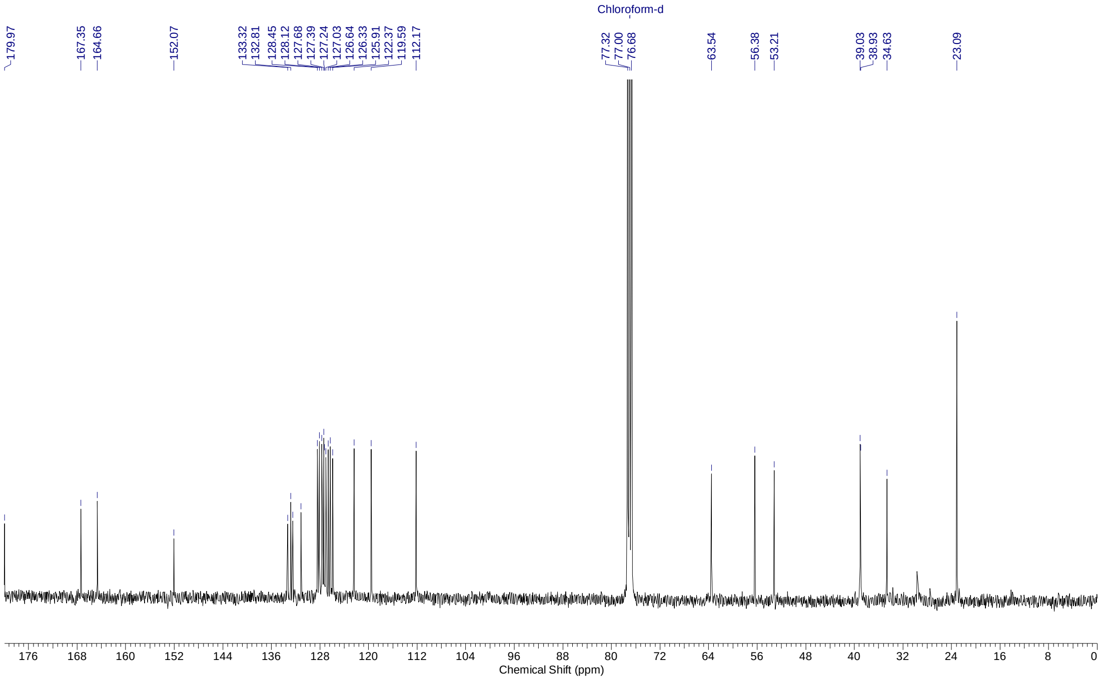

Figure S48 ^{13}C NMR spectrum of compound **10i** (100 MHz, $CDCl_3$).

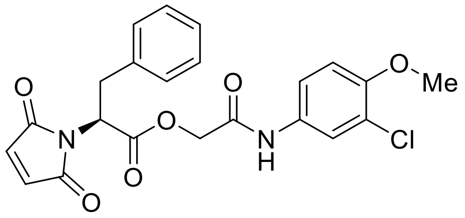

Acquisition Time (sec)	1.3282	Comment	Rafael "RAF-184" cdcl3/ AV600MHz abr12rafH2			Date	13 Apr 2016 07:34:08
File Name	E:\RMN Dissert\600 MHz\Abr 16\abr12rafH2 RAF 184_001001r			Frequency (MHz)	600.17	Nucleus	1H
Number of Transients	16	Original Points Count	16384	Points Count	65536	Pulse Sequence	zg30
Solvent	CHLOROFORM-D	Sweep Width (Hz)	12335.53	Temperature (degree C)	24.477		

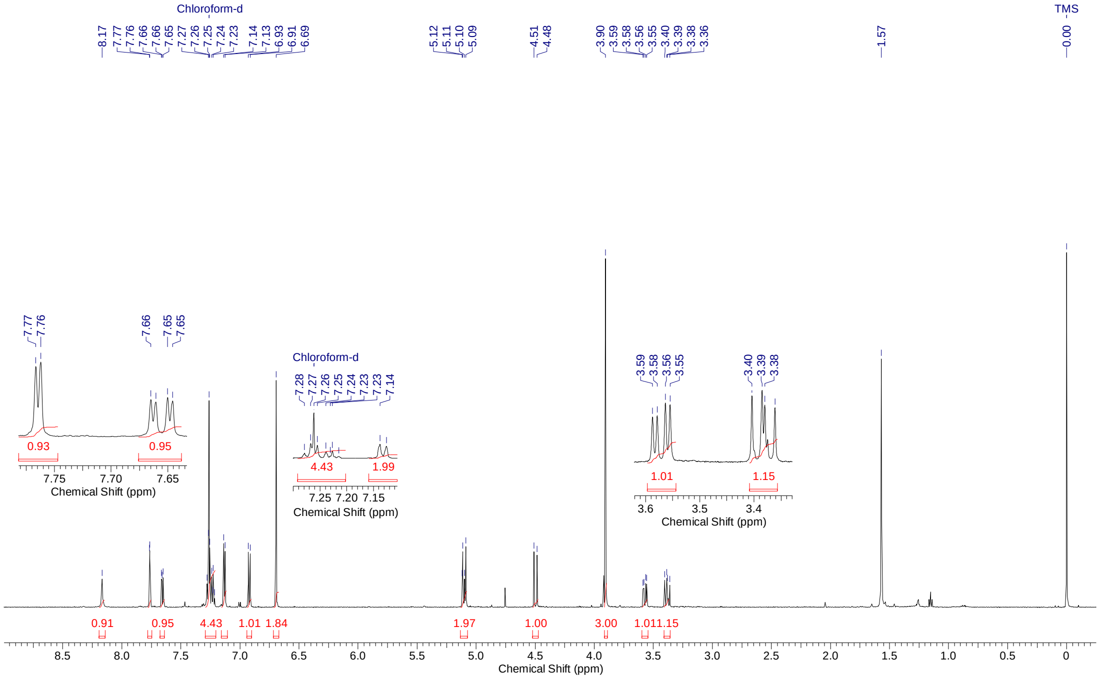

Figure S49 ^{1}H NMR spectrum of compound **10j** (600 MHz, $CDCl_3$).

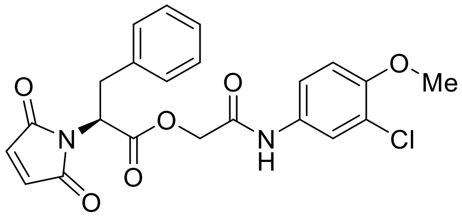

Acquisition Time (sec)	0.4020	***Comment***	Rafael "RAF-184" cdcl3/ AV600MHz abr12rafH2 - 13C			***Date***	13 Apr 2016 07:34:40
File Name	E:\RMN Dissert\600 MHz\Abr 16\abr12rafH2 RAF 184_002001r			***Frequency (MHz)***	150.91	***Nucleus***	13C
Number of Transients	5120	***Original Points Count***	16384	***Points Count***	32768	***Pulse Sequence***	zgpg30
Solvent	CHLOROFORM-D	***Sweep Width (Hz)***	40760.87	***Temperature (degree C)***	24.476		

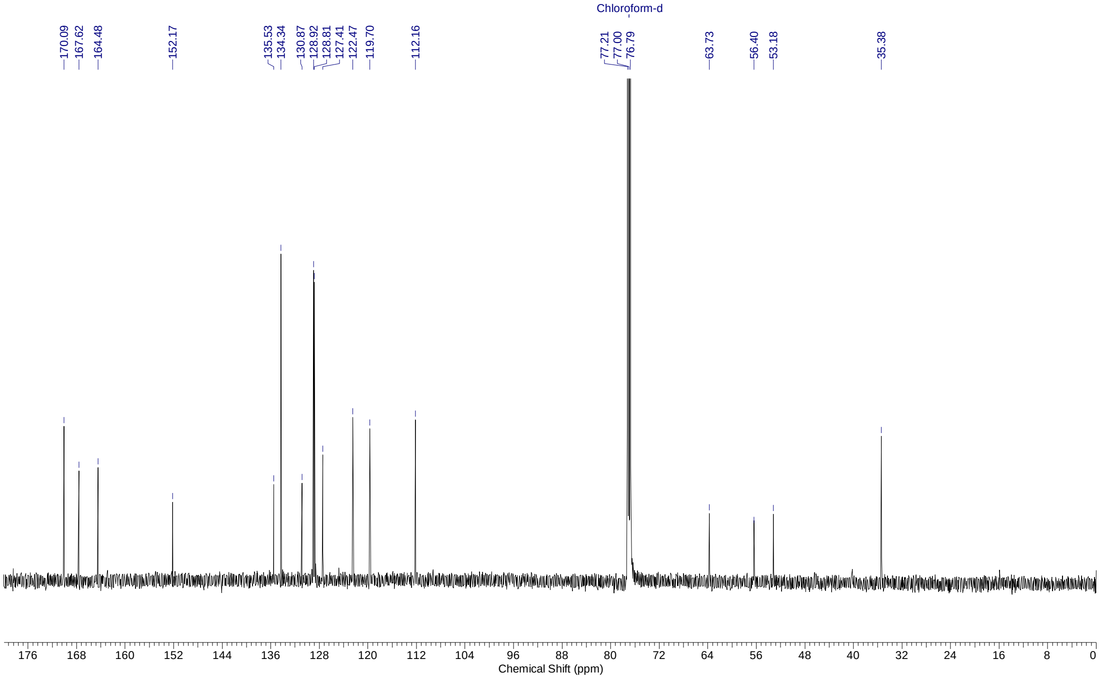

Figure S50 ^{13}C NMR spectrum of compound **10j** (150 MHz, $CDCl_3$).

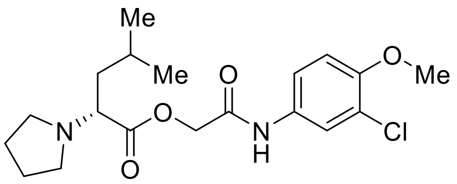

Acquisition Time (sec)	1.5903	***Comment***	Rafael - RAF-101 - CDCl3 - Avance 500 MHz - ago18rafH1 - ¹H			***Date***	28 Aug 2015 12:56:44
File Name	E:\RMN Dissert\500 MHz\Ago 15\ago28rafH1 RAF 101_001001r			***Frequency (MHz)***	499.87	***Nucleus***	1H
Number of Transients	4	***Original Points Count***	16384	***Points Count***	65536	***Pulse Sequence***	zg30
Solvent	CHLOROFORM-D	***Sweep Width (Hz)***	10302.20	***Temperature (degree C)***	25.149		

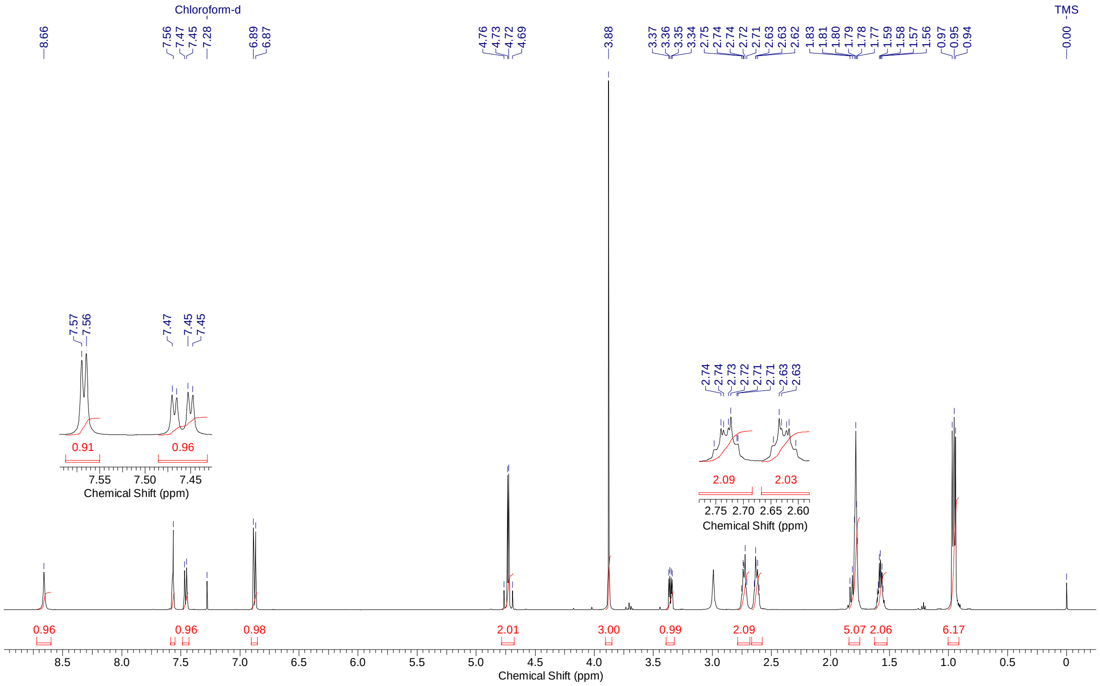

Figure S51 ¹H NMR spectrum of compound **10k** (500 MHz, $CDCl_3$).

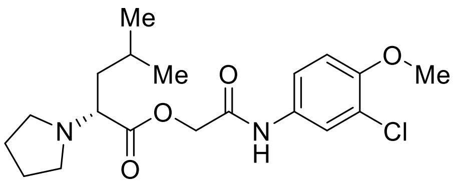

Acquisition Time (sec)	0.4981	Comment	Rafael - RAF-101 - CDCl3 - Avance 500 MHz - ago18rafH1 - ¹³C	Date	28 Aug 2015 12:56:54
File Name	E:\RMN Dissert\500 MHz\Ago 15\ago28rafH1 RAF 101_002001r	Frequency (MHz)	125.69	Nucleus	13C
Number of Transients	36	Original Points Count 16384	Points Count 32768	Pulse Sequence	zgpg30
Solvent	CHLOROFORM-D	Sweep Width (Hz) 32894.74	Temperature (degree C) 25.215		

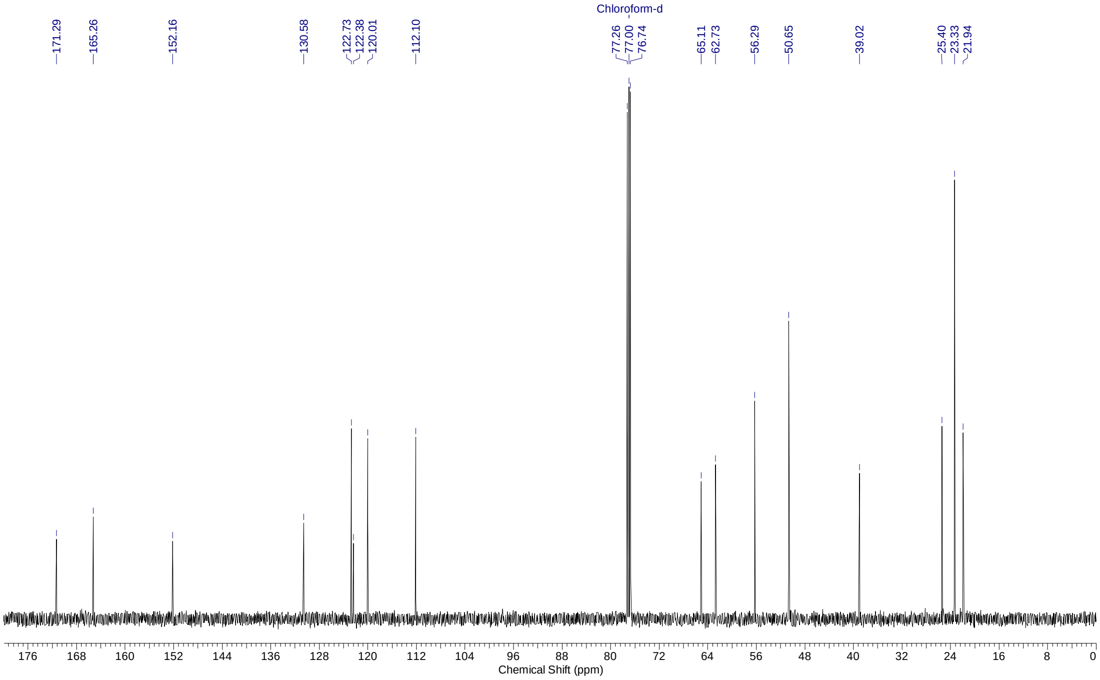

Figure S52 ^{13}C NMR spectrum of compound **10k** (125 MHz, $CDCl_3$).

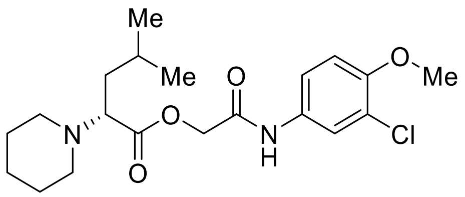

Acquisition Time (sec)	1.5903	***Comment***	Rafael "RAF-115" cdcl3/ Av500MHz set22rafH1			***Date***	23 Sep 2015 07:46:54
File Name	E:\RMN Dissert\500 MHz\Set 15\set22rafH1 RAF 115_001001r			***Frequency (MHz)***	499.87	***Nucleus***	1H
Number of Transients	16	***Original Points Count***	16384	***Points Count***	65536	***Pulse Sequence***	zg30
Solvent	CHLOROFORM-D	***Sweep Width (Hz)***	10302.20	***Temperature (degree C)***	25.147		

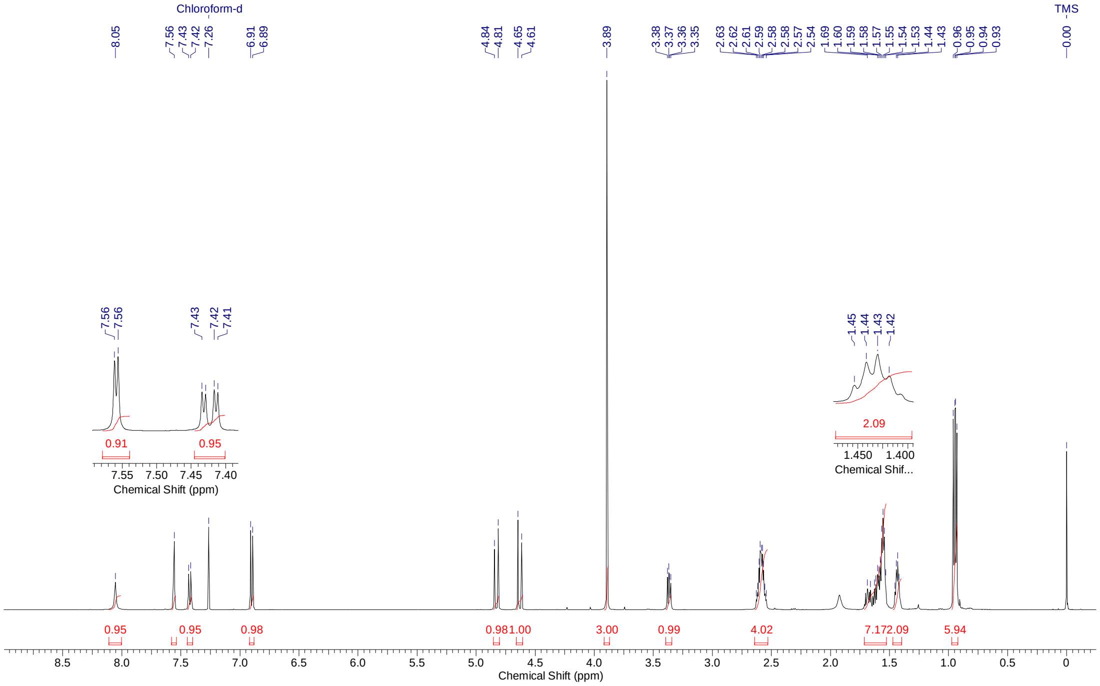

Figure S53 ^{1}H NMR spectrum of compound **10l** (500 MHz, $CDCl_3$).

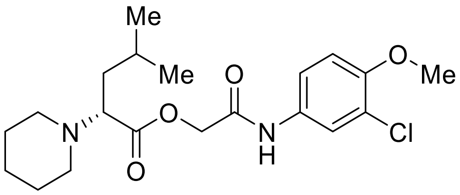

Acquisition Time (sec)	0.4981	***Comment***	Rafael "RAF-115" cdcl3/ Av500MHz set22rafH1 - 13C			***Date***	23 Sep 2015 07:46:40
File Name	E:\RMN Dissert\500 MHz\Set 15\set22rafH1 RAF 115_002001r			***Frequency (MHz)***	125.69	***Nucleus***	13C
Number of Transients	1024	***Original Points Count***	16384	***Points Count***	32768	***Pulse Sequence***	zgpg30
Solvent	CHLOROFORM-D	***Sweep Width (Hz)***	32894.74	***Temperature (degree C)***	25.152		

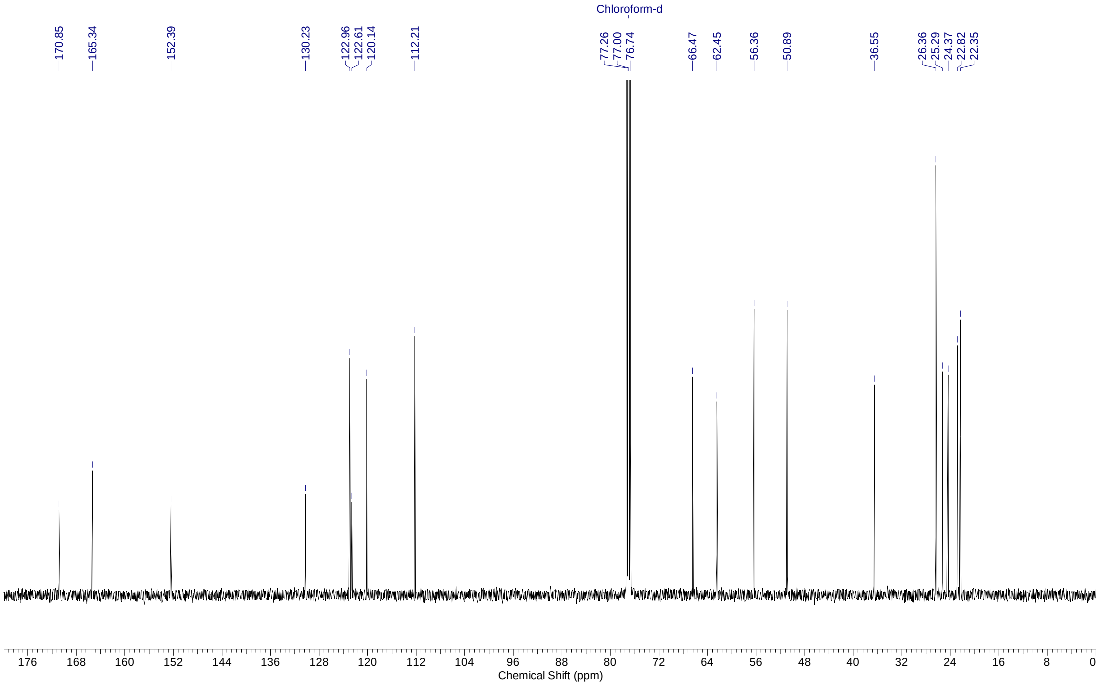

Figure S54 ^{13}C NMR spectrum of compound **10l** (125 MHz, $CDCl_3$).

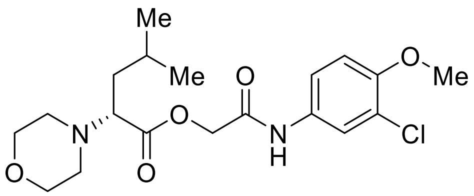

Acquisition Time (sec)	2.0447	Comment	Rafael "RAF-128" cdcl3/ Av400MHz out19rafH1	Date	20 Oct 2015 06:38:32
File Name	E:\RMN Dissert\400 MHz\Out 15\out19rafH1 RAF 128_001001r	Frequency (MHz)	400.18	Nucleus	1H
Number of Transients	16	Original Points Count 16384	Points Count 65536	Pulse Sequence	zg30
Solvent	CHLOROFORM-D		Sweep Width (Hz) 8012.82	Temperature (degree C)	25.260

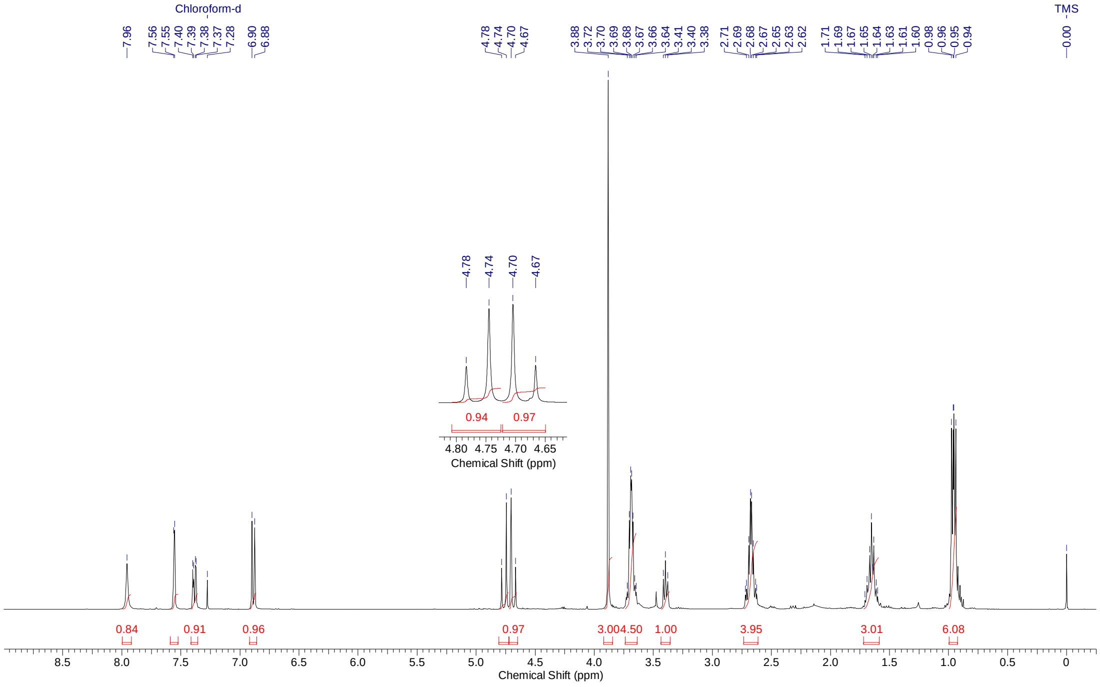

Figure S55 ^{1}H NMR spectrum of compound **10m** (400 MHz, $CDCl_3$).

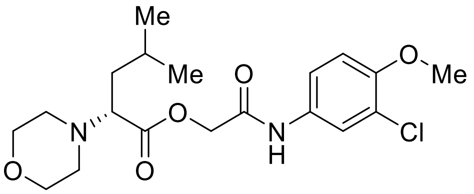

Acquisition Time (sec)	0.6816	***Comment***	Rafael "RAF-128" cdcl3/ Av400MHz out19rafH1 - 13C			***Date***	20 Oct 2015 06:38:26
File Name	E:\RMN Dissert\400 MHz\Out 15\out19rafH1 RAF 128_002001r			***Frequency (MHz)***	100.63	***Nucleus***	13C
Number of Transients	1024	***Original Points Count***	16384	***Points Count***	65536	***Pulse Sequence***	zgpg30
Solvent	CHLOROFORM-D	***Sweep Width (Hz)***	24038.46	***Temperature (degree C)***	25.160		

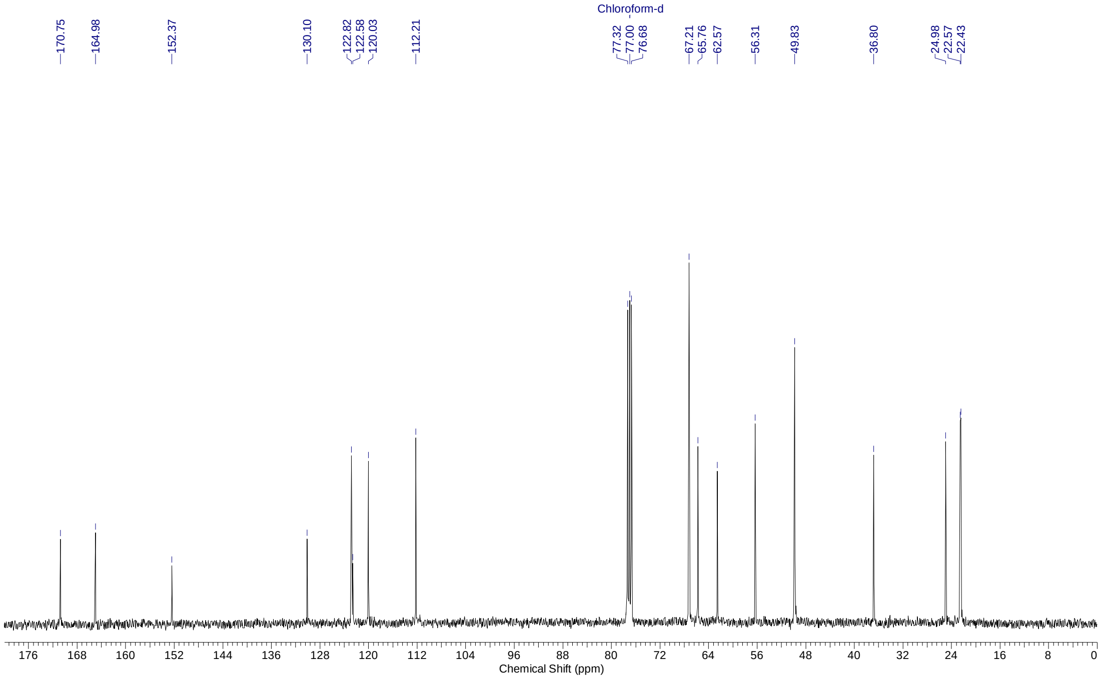

Figure S56 ^{13}C NMR spectrum of compound **10m** (100 MHz, $CDCl_3$).

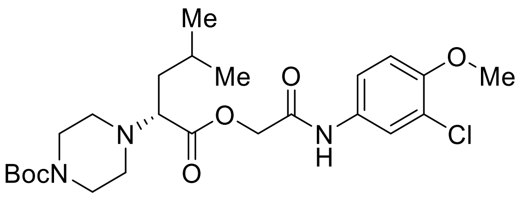

Acquisition Time (sec)	1.5903	***Comment***	Rafael - RAF-132 -CDCl3 - Av.500MHz - out23rafH1			***Date***	15 Jul 2015 09:33:14
File Name	E:\RMN Dissert\500 MHz\Out 15\out23rafH1 RAF 132_001001r			***Frequency (MHz)***	499.87	***Nucleus***	1H
Number of Transients	16	***Original Points Count***	16384	***Points Count***	65536	***Pulse Sequence***	zg30
Solvent	CHLOROFORM-D	***Sweep Width (Hz)***	10302.20	***Temperature (degree C)***	25.145		

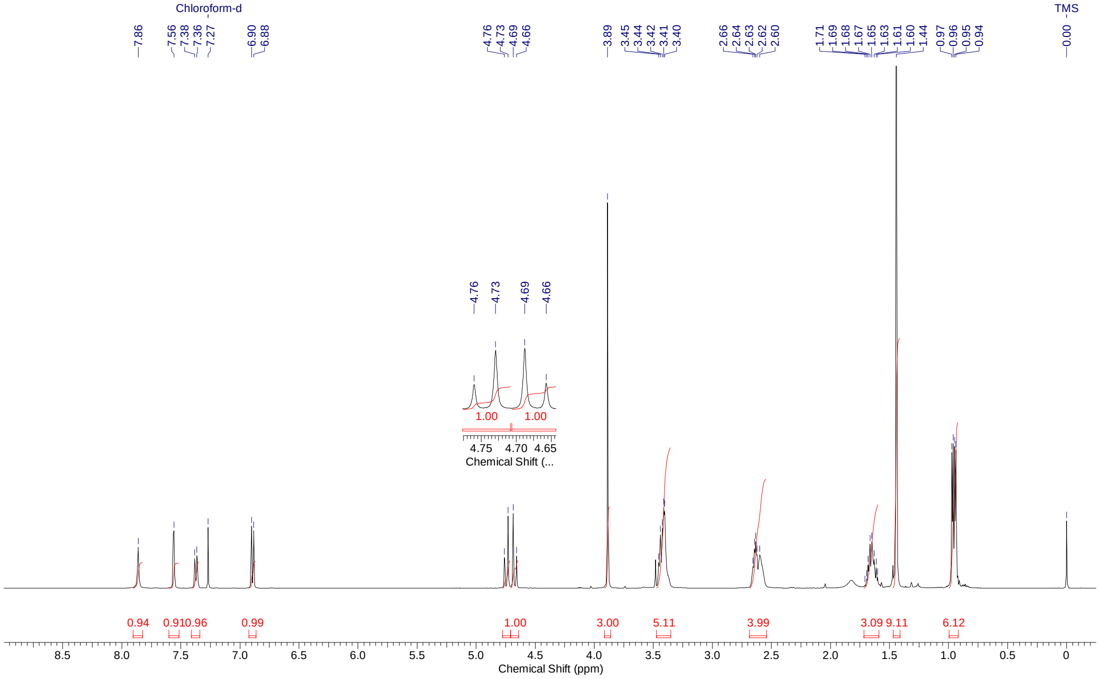

Figure S57 ^{1}H NMR spectrum of compound **10n** (500 MHz, $CDCl_3$).

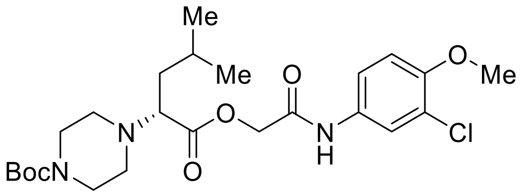

Acquisition Time (sec)	0.4981	Comment	Rafael - RAF-132 -CDCl3 - Av.500MHz - out23rafC1 - 13C			Date	15 Jul 2015 16:03:30
File Name	E:\RMN Dissert\500 MHz\Out 15\out23rafH1 RAF 132_002001r			Frequency (MHz)	125.69	Nucleus	13C
Number of Transients	512	Original Points Count	16384	Points Count	32768	Pulse Sequence	zgpg30
Solvent	CHLOROFORM-D	Sweep Width (Hz)	32894.74	Temperature (degree C)	25.150		

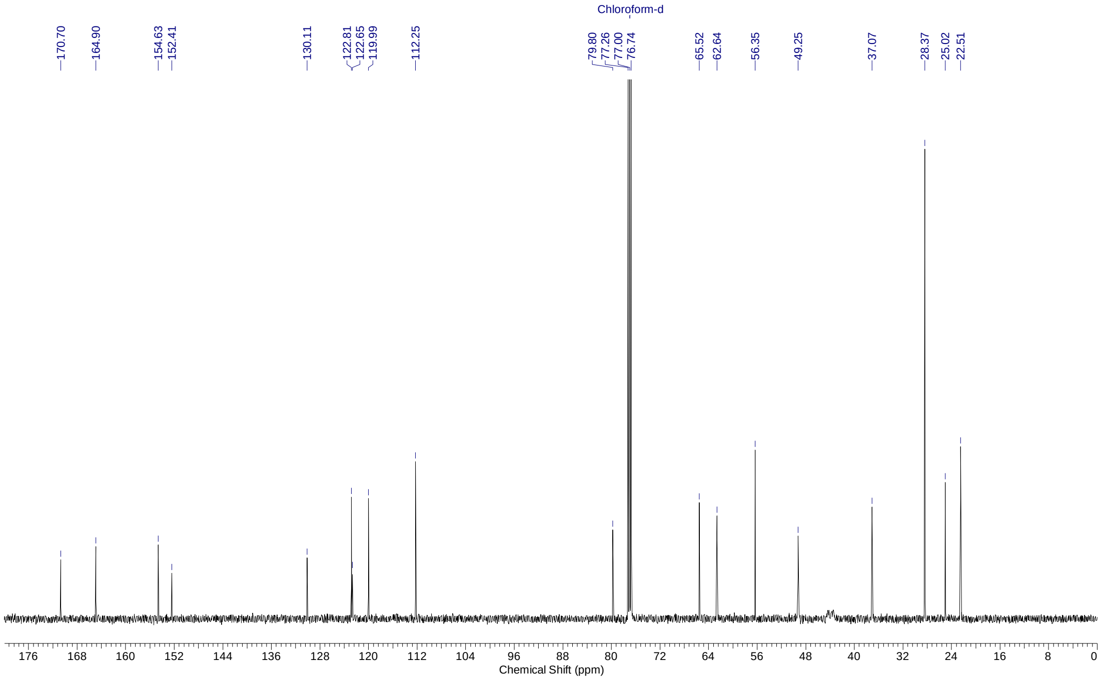

Figure S58 ^{13}C NMR spectrum of compound **10n** (125 MHz, $CDCl_3$).

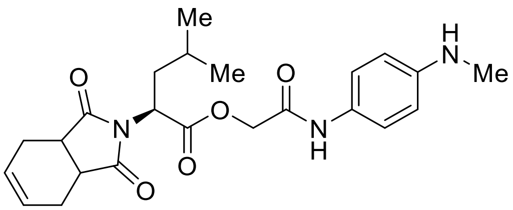

Acquisition Time (sec)	1.6384	Comment	Rafael - RAF-26 - CDCl3 - Av 500MHz - dez18rafH1			Date	19 Dec 2014 06:05:06
File Name	C:\UNICAMP\Mestrado\RMN\500 MHz\2014\Dez\dez18rafH1_001001r					Frequency (MHz)	499.87
Nucleus	1H	Number of Transients	16	Original Points Count	16384	Points Count	65536
Pulse Sequence	zg30	Solvent	CHLOROFORM-D	Sweep Width (Hz)	10000.00	Temperature (degree C)	25.146

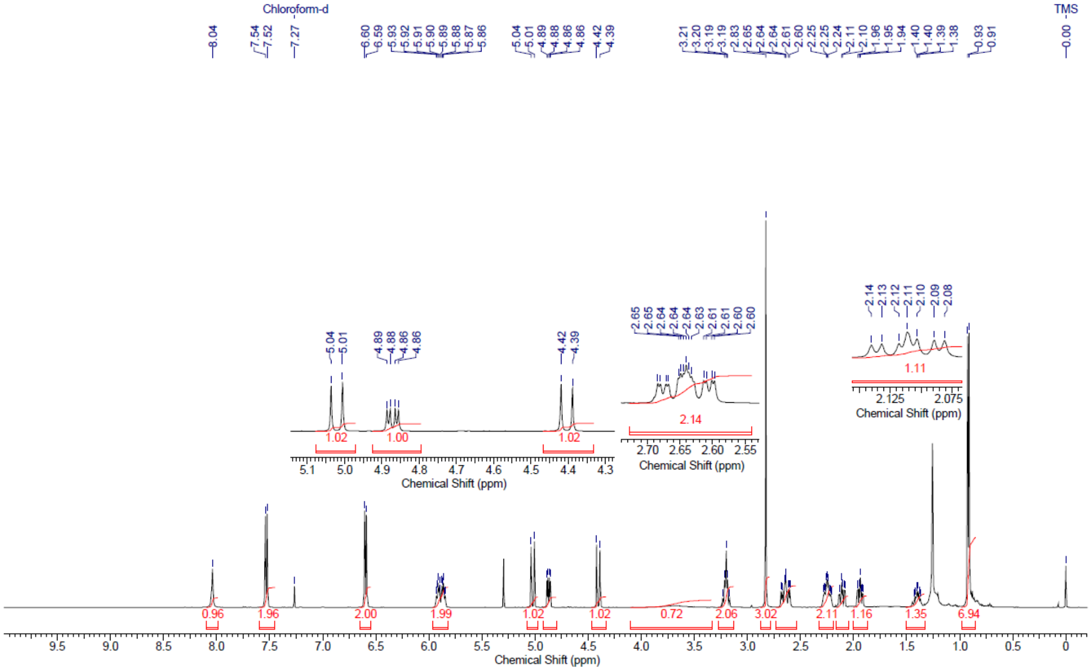

Figure S59 ^{1}H NMR spectrum of compound **3k'** (500 MHz, $CDCl_3$).

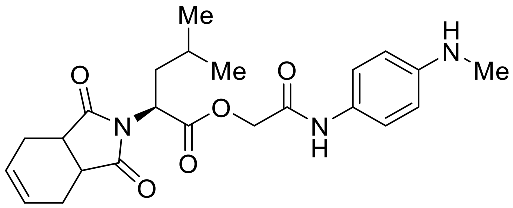

Acquisition Time (sec)	0.5505	*Comment*	Rafael - RAF-26 - CDCl3 - Av 500MHz - dez18rafH1 13C			*Date*	19 Dec 2014 06:05:20
File Name	C:\UNICAMP\Mestrado\RMN\500 MHz\2014\Dez\dez18rafH1_002001			*Frequency (MHz)*	125.69	*Nucleus*	13C
Number of Transients	3072	*Original Points Count*	16384	*Points Count*	32768	*Pulse Sequence*	zgpg30
Solvent	CHLOROFORM-D	*Sweep Width (Hz)*	29761.90	*Temperature (degree C)*	25.150		

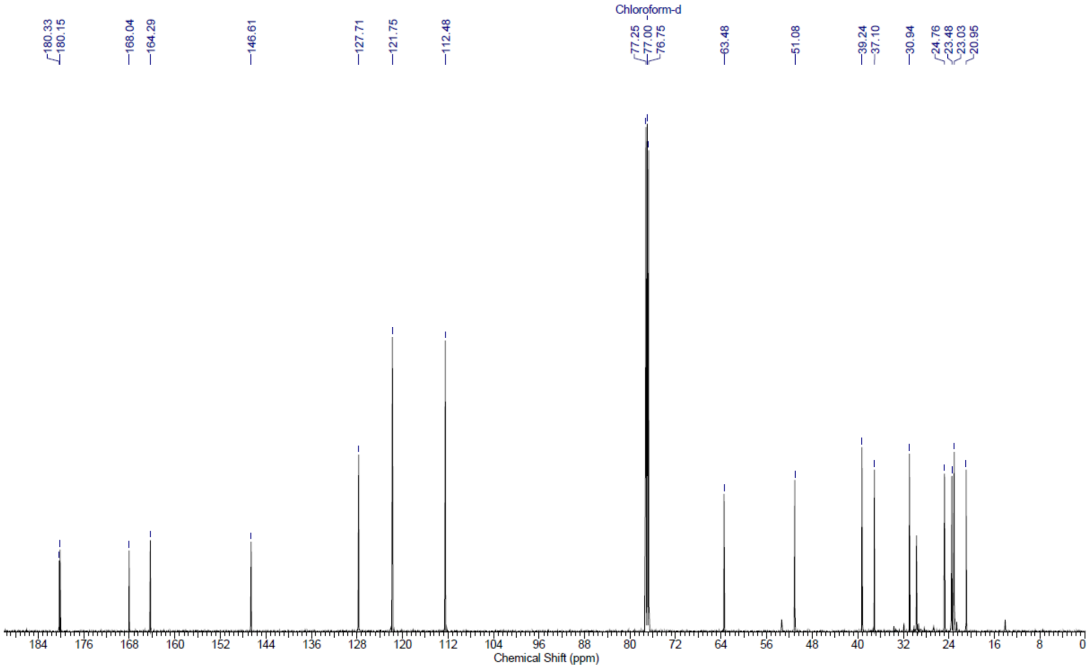

Figure S60 ^{13}C NMR spectrum of compound **3k’** (125 MHz, $CDCl_3$).

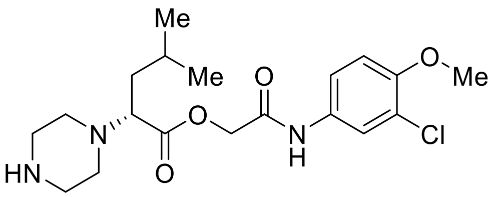

Acquisition Time (sec)	1.5903	***Comment***	Rafael - RAF 133 - MeOD - Avance 500 MHz - out26rafH2 - 1H			***Date***	22 Aug 2015 19:29:56
File Name	E:\RMN Dissert\500 MHz\Out 15\out26rafH2 RAF 133_001001r			***Frequency (MHz)***	499.87	***Nucleus***	1H
Number of Transients	16	***Original Points Count***	16384	***Points Count***	65536	***Pulse Sequence***	zg30
Solvent	METHANOL-d4	***Spectrum Offset (Hz)***	3067.9211	***Sweep Width (Hz)***	10302.20	***Temperature (degree C)***	50.712

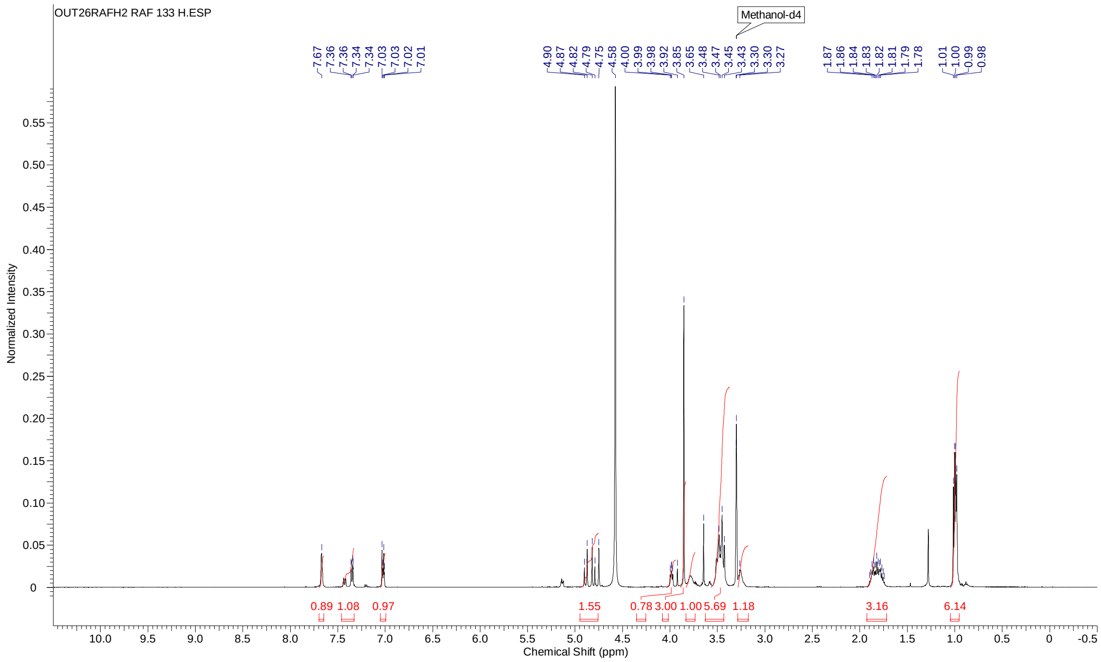

Figure S61 ^{1}H NMR spectrum of compound **10o** (500 MHz, MeOD).

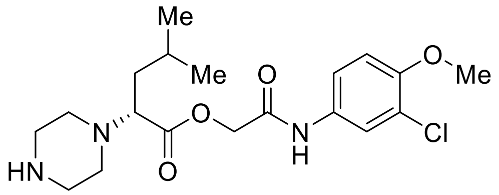

Acquisition Time (sec)	0.4981	***Comment***	Rafael - RAF 133 - MeOD - Avance 500 MHz - out26rafH2 - 13C 50 ºC			***Date***	26 Oct 2015 14:29:52
Date Stamp	26 Oct 2015 14:29:52	***File Name***	C:\USERS\RAFAEL\DROPBOX\MESTRADO\DISSERTAçãO\RMN DISSERT\500 MHZ\OUT 15\OUT26RAFH2 RAF 133\6\PDATA\1\1R				
Frequency (MHz)	125.69	***Nucleus***	13C	***Number of Transients***	3072	***Origin***	spect
Original Points Count	16384	***Owner***	nmrsu	***Points Count***	32768	***Pulse Sequence***	zgpg30
Receiver Gain	2050.00	***SW(cyclical) (Hz)***	32894.74	***Solvent***	METHANOL-d4	***Spectrum Offset (Hz)***	14019.4951
Spectrum Type	STANDARD	***Sweep Width (Hz)***	32893.73	***Temperature (degree C)***	50.152		

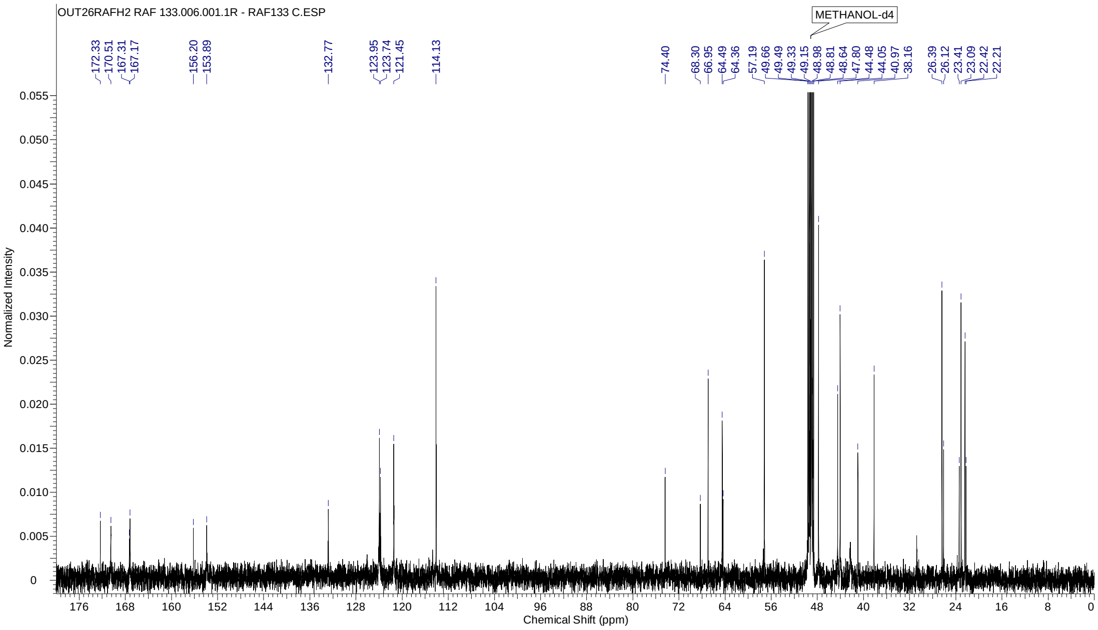

Figure S62 ^{13}C NMR spectrum of compound **10o** (125 MHz, MeOD).

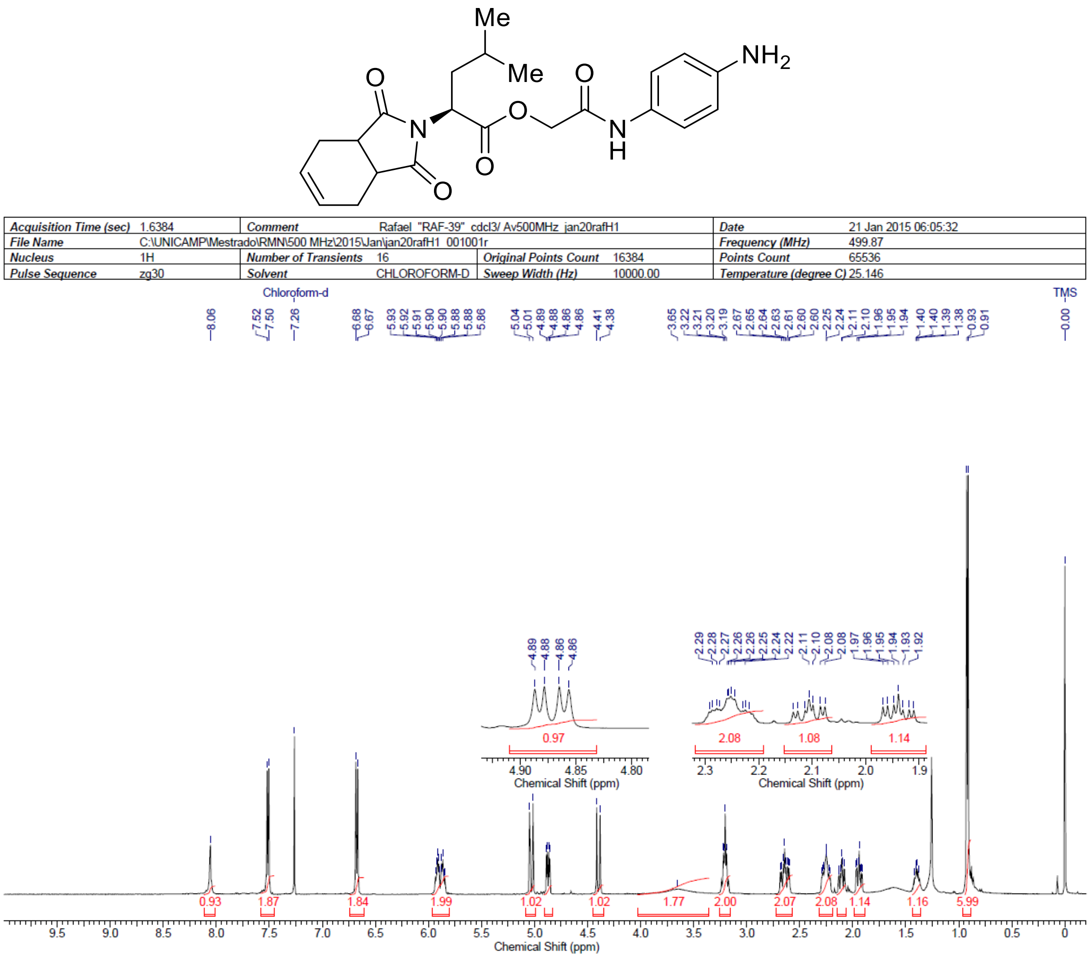

Figure S63 ^{1}H NMR spectrum of compound **3l'** (500 MHz, $CDCl_3$).

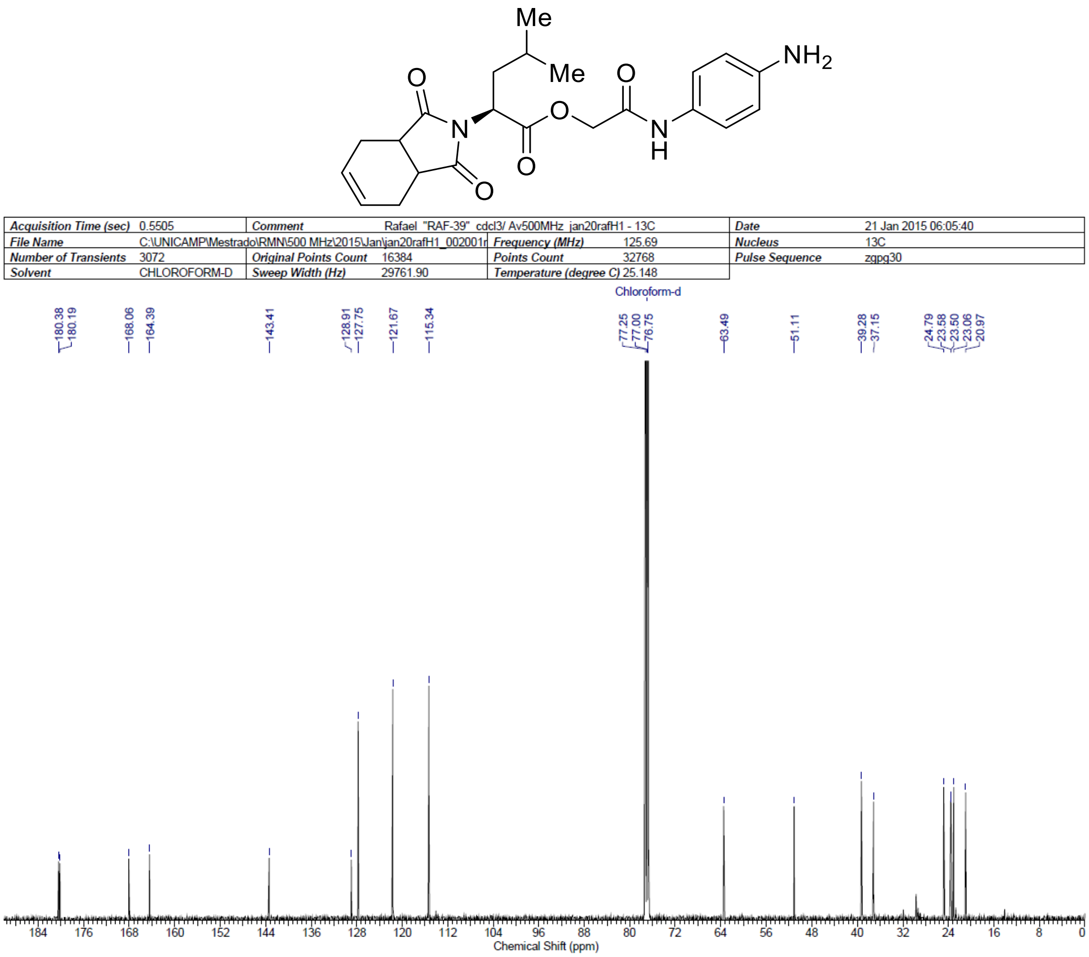

Figure S64 ^{13}C NMR spectrum of compound **3l’** (125 MHz, $CDCl_3$).

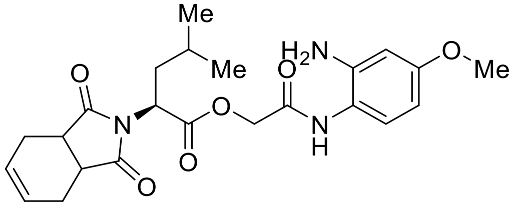

Acquisition Time (sec)	1.6384	Comment	Rafael - RAF-53b - CDCl3 - Avance 500 MHz - fev27rafH3 - 1H	Date	27 Feb 2015 13:03:10
File Name	C:\UNICAMP\Mestrado\RMN\500 MHz\2015\Fev\fev27rafH3_001001r	Frequency (MHz)	499.87	Nucleus	1H
Number of Transients	16	Original Points Count	16384	Points Count	65536	Pulse Sequence	zg30
Solvent	CHLOROFORM-D	Sweep Width (Hz)	10000.00	Temperature (degree C)	25.151

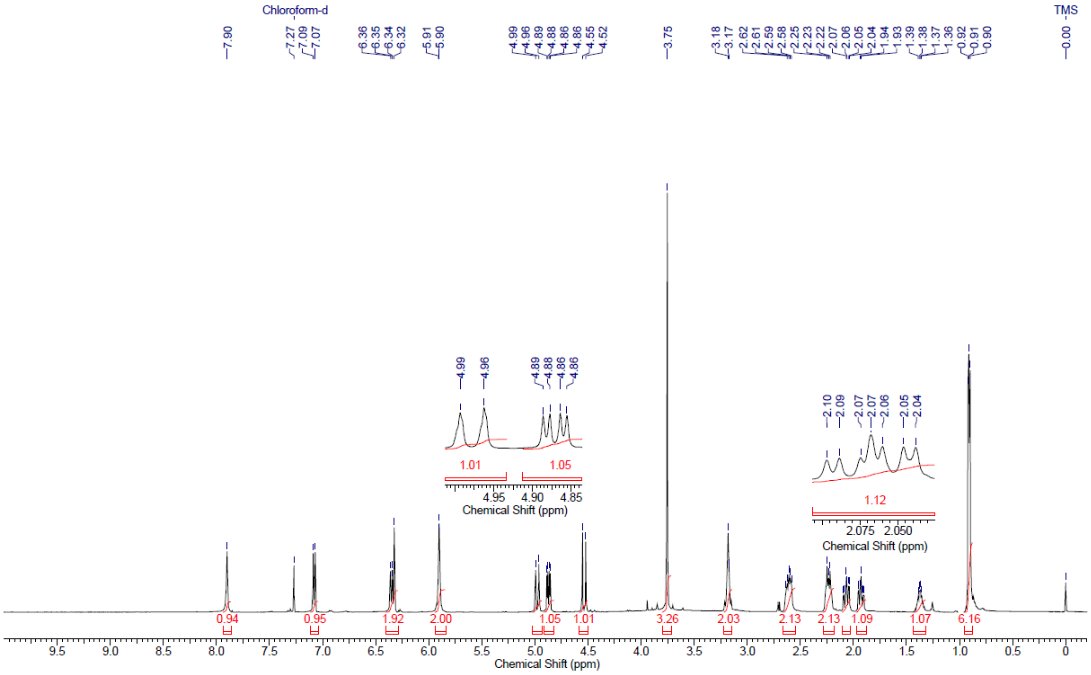

Figure S65 ^{1}H NMR spectrum of compound **3m'** (500 MHz, $CDCl_3$).

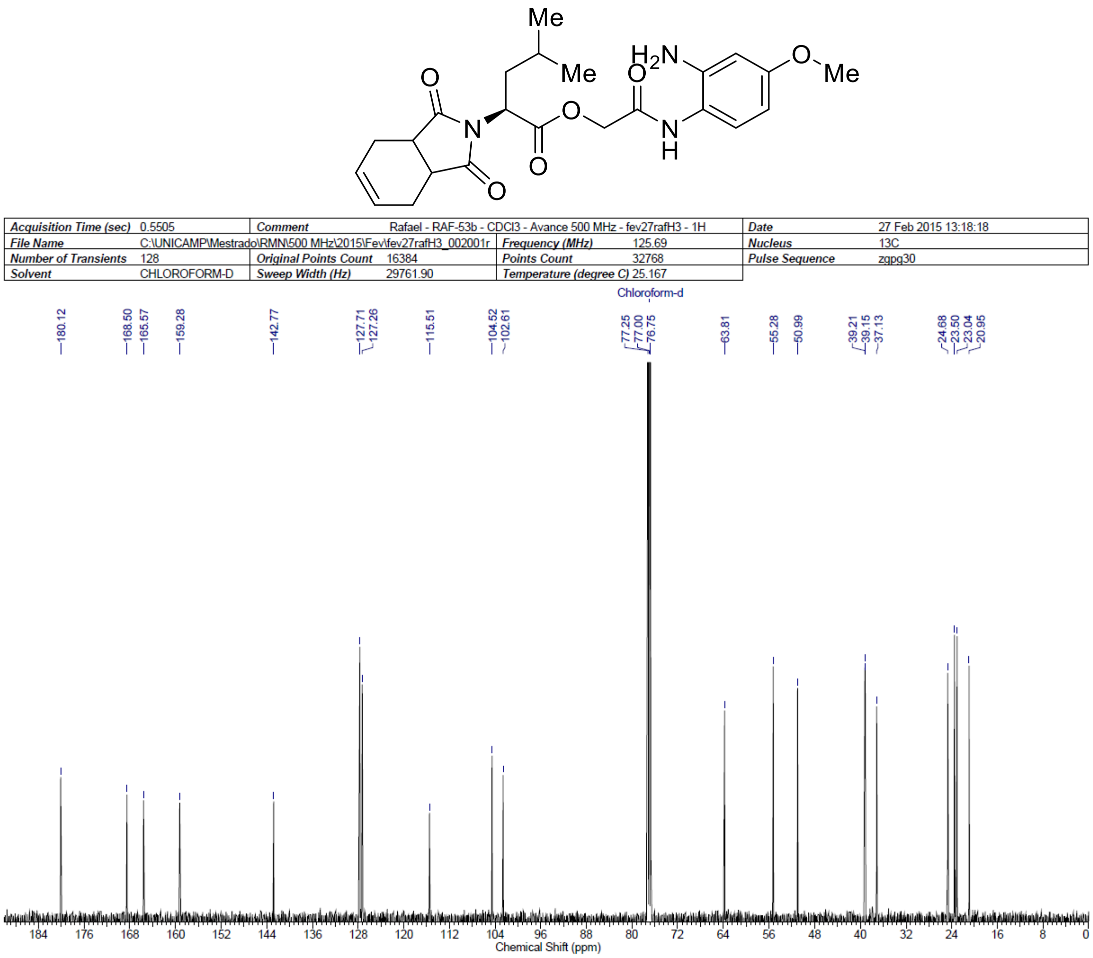

Figure S66 ^{13}C NMR spectrum of compound **3m’** (125 MHz, $CDCl_3$).

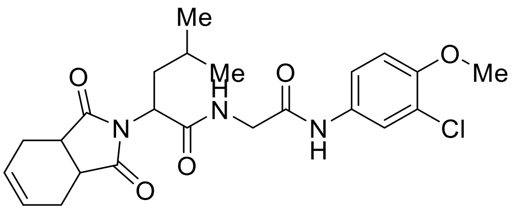

Acquisition Time (sec)	3.1654	*Comment*	Thiago_DMSO_250mHZ_TSS-037_set25tssH3			*Date*	25 Sep 2012 15:50:40
Date Stamp	25 Sep 2012 15:50:40			*File Name*	C:\USERS\RAFAEL\DESKTOP\ESPECTROS TSS\250 - 2012\SET25TSSH3\1\PDATA\1\1R		
Frequency (MHz)	250.13	*Nucleus*	1H	*Number of Transients*	16	*Origin*	spect
Original Points Count	16384	*Owner*	root	*Points Count*	32768	*Pulse Sequence*	zg30
Receiver Gain	406.40	*SW(cyclical) (Hz)*	5175.98	*Solvent*	DMSO-d6	*Spectrum Offset (Hz)*	1755.9128
Spectrum Type	STANDARD	*Sweep Width (Hz)*	5175.83	*Temperature (degree C)*	25.160		

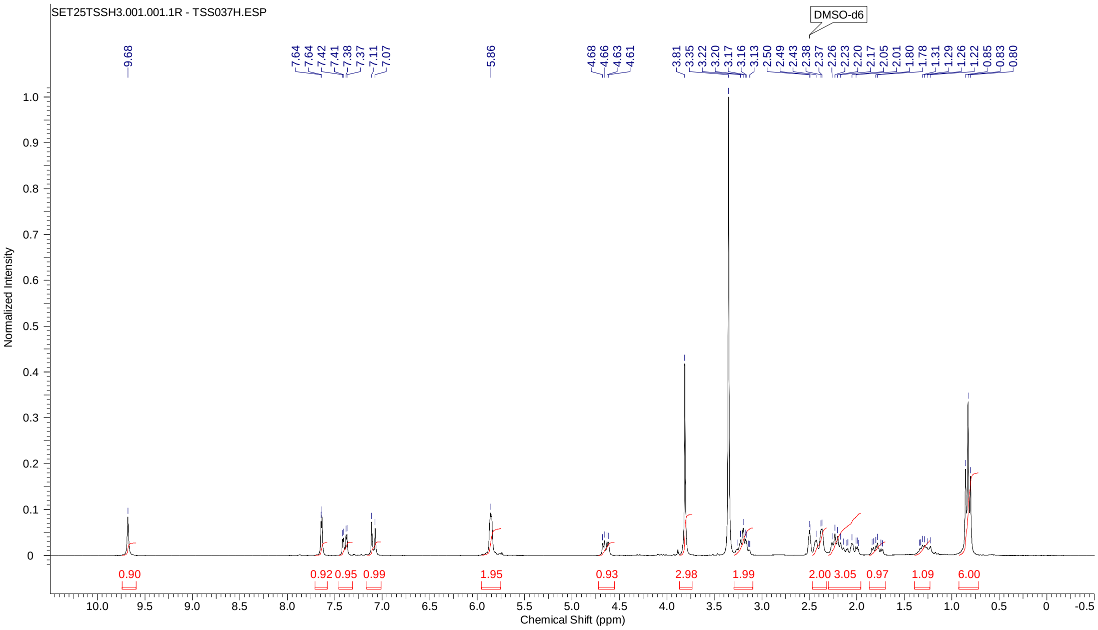

Figure S67 ^{1}H NMR spectrum of compound **5** (250 MHz, DMSO).

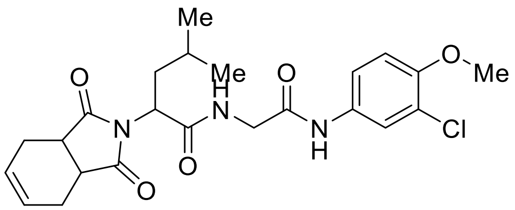

Acquisition Time (sec)	0.5439	***Comment***	Thiago_DMSO_250mHZ_TSS-037_set25tssC3			***Date***	25 Sep 2012 16:07:44
Date Stamp	25 Sep 2012 16:07:44			***File Name***	C:\USERS\RAFAEL\DESKTOP\ESPECTROS TSS\250 - 2012\SET25TSSC3\1\PDATA\1\1R		
Frequency (MHz)	62.90	***Nucleus***	13C	***Number of Transients***	1024	***Origin***	spect
Original Points Count	8192	***Owner***	root	***Points Count***	32768	***Pulse Sequence***	zgpg30
Receiver Gain	724.10	***SW(cyclical) (Hz)***	15060.24	***Solvent***	DMSO-d6	***Spectrum Offset (Hz)***	6262.6899
Spectrum Type	STANDARD	***Sweep Width (Hz)***	15059.78	***Temperature (degree C)***	25.160		

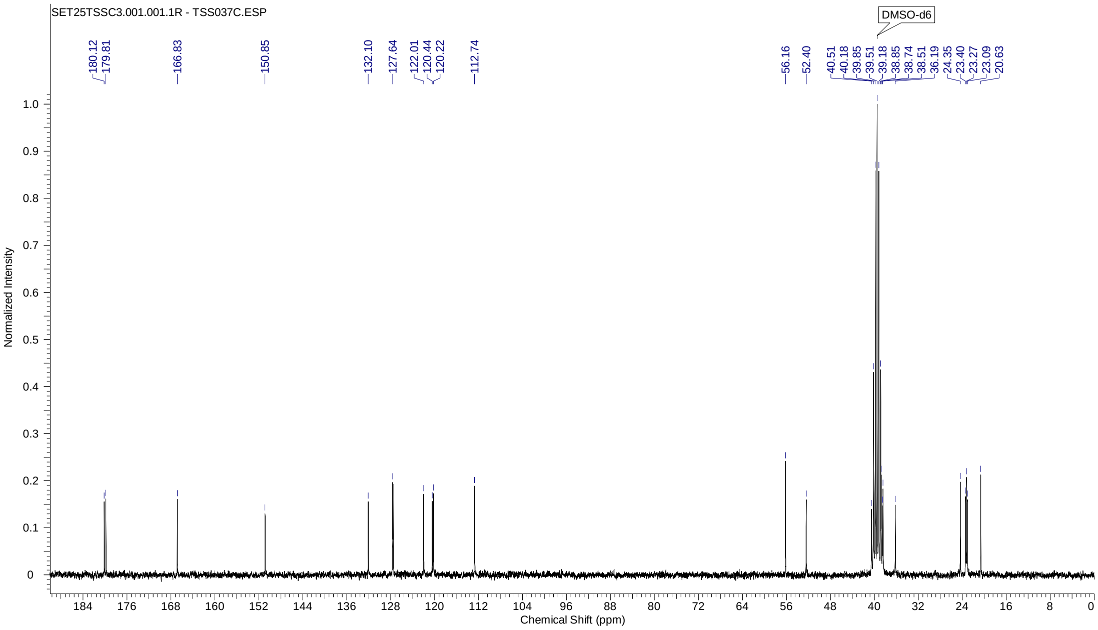

Figure S68 ^{13}C NMR spectrum of compound **5** (62.5 MHz, DMSO).

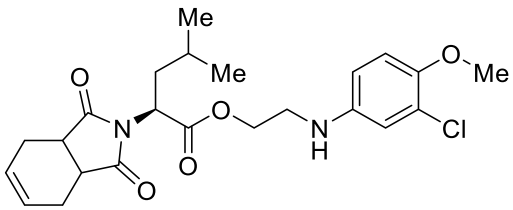

Acquisition Time (sec)	1.3282	***Comment***	Rafael "RAF-151" cdcl3/ Av600MHz dez03rafH1			***Date***	03 Dec 2015 16:33:46
File Name	E:\RMN Dissert\600 MHz\Dez 15\dez03rafH1 RAF 151_001001r			***Frequency (MHz)***	600.17	***Nucleus***	1H
Number of Transients	16	***Original Points Count***	16384	***Points Count***	65536	***Pulse Sequence***	zg30
Solvent	CHLOROFORM-D	***Sweep Width (Hz)***	12335.53	***Temperature (degree C)***	25.155		

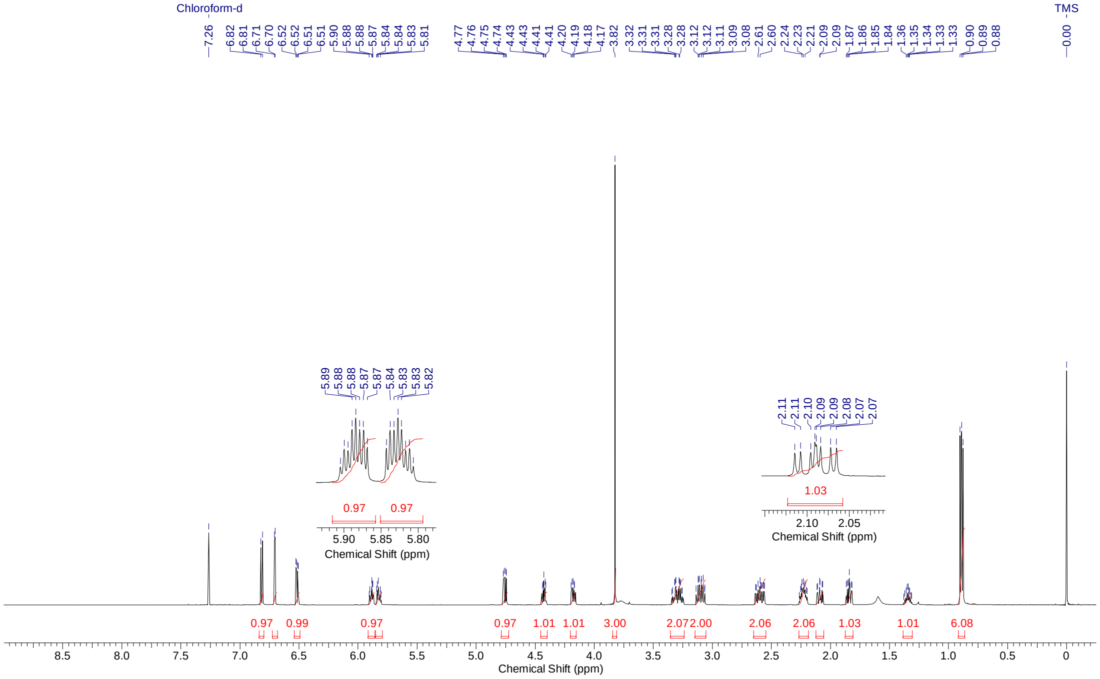

Figure S69 ^{1}H NMR spectrum of compound **7** (600 MHz, $CDCl_3$).

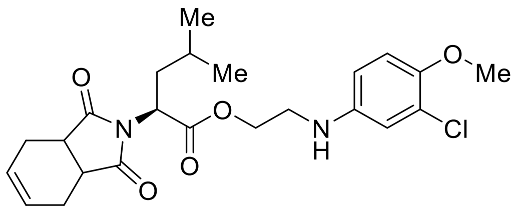

Acquisition Time (sec)	0.4544	Comment	Rafael "RAF-151" cdcl3/ Av600MHz dez03rafH1 - 13C			Date	03 Dec 2015 16:53:20
File Name	E:\RMN Dissert\600 MHz\Dez 15\dez03rafH1 RAF 151_002001r			Frequency (MHz)	150.91	Nucleus	13C
Number of Transients	320	Original Points Count	16384	Points Count	32768	Pulse Sequence	zgpg30
Solvent	CHLOROFORM-D	Sweep Width (Hz)	36057.69	Temperature (degree C)	25.145		

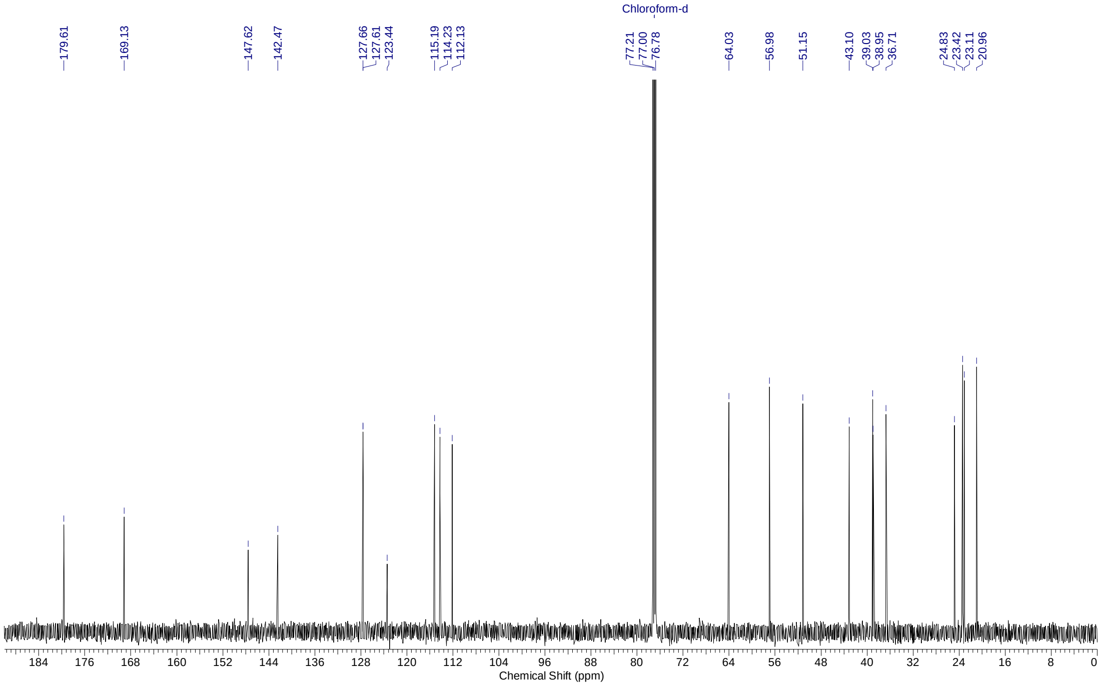

Figure S70 ^{13}C NMR spectrum of compound **7** (150 MHz, $CDCl_3$).
